# Improving farm-level antimicrobial stewardship benchmarks by reporting antimicrobial use within the context of both the magnitude of disease pressure and the outcome of therapy

**DOI:** 10.3389/fvets.2022.1022557

**Published:** 2022-10-05

**Authors:** Nora F. D. Schrag, Sandra M. Godden, Randall S. Singer, Jason E. Lombard, John R. Wenz, David E. Amrine, Brian V. Lubbers, Michael D. Apley

**Affiliations:** ^1^College of Veterinary Medicine, Department of Clinical Sciences Kansas State University, Manhattan, KS, United States; ^2^Livestock Veterinary Resources, LLC, Manhattan, KS, United States; ^3^Department of Veterinary Population Medicine, University of Minnesota, Saint Paul, MN, United States; ^4^Department of Veterinary and Biomedical Sciences, University of Minnesota, Saint Paul, MN, United States; ^5^Mindwalk Consulting Group, Falcon Heights, MN, United States; ^6^USDA Animal and Plant Health Inspection Service, Field Epidemiological Investigation Services, Fort Collins, CO, United States; ^7^Department of Veterinary Clinical Science, College of Veterinary Medicine, Washington State University, Pullman, WA, United States; ^8^The HEALTHSUM Syndicate, LLC, Sunnyside, WA, United States

**Keywords:** antimicrobial use, livestock, benchmarking, monitoring, antimicrobial stewardship, dairy, pharmacoepidemiology

## Abstract

This manuscript explores a method of benchmarking antimicrobial use within the context of farm level therapeutic incidence (a proxy for disease incidence), and the outcome of that therapy. This is reported both within the same farm over time (2016–2019), as well as evaluated across participating farms. Reporting antimicrobial use in this format addresses multiple primary questions necessary for evaluating on farm antimicrobial stewardship: How much disease is recorded? How much antimicrobial use is recorded? How often are antimicrobials included in therapy for each disease? What is the outcome of therapy? The three primary metrics reported are: therapeutic events per 100 cow years (TE/100CY), antimicrobial regimens per 100 cow years (REG/100CY), and the percent therapeutic success (% Success). Success was defined as: the cow remained in the herd and had no further TE recorded within 30 days of the end of the TE being evaluated. These measures identify opportunities for change on an individual farm, such as improvement in disease prevention, or a change in choices about when to include an antimicrobial in the treatment protocol. Therapeutic outcomes provide additional context, in some instances demonstrating differences in recording practices and case definitions, while in other cases serving to safeguard animal welfare as efforts are made to decrease antimicrobial use in the future. Although developed for farm level reporting, the metrics may also be more broadly summarized to meet future reporting requirements for marketing chain or national level antimicrobial use reports. The process outlined here serves as a prototype to be considered when developing antimicrobial use reporting systems where farm level antimicrobial stewardship is the primary objective.

## Introduction

Benchmarking farm antimicrobial use has been promoted as a mechanism for improving antimicrobial stewardship (1). Although numerous methods for quantifying antimicrobial use have been described and implemented, metrics vary greatly in their level of granularity and the amount of farm level context available to improve interpretation (2–5). If benchmarking at the farm level is to be used as a tool for improved stewardship, metrics should be sufficiently detailed at the level of the farm where use occurred (5). Creating actionable change is a challenge, but it has been suggested that improvement in both veterinarian and farmer confidence in making treatment decisions will likely improve antimicrobial use on farms (6). This manuscript explores a method of benchmarking antimicrobial use within the context of farm level therapeutic incidence (a proxy for disease incidence), and the outcome of that therapy. This is reported both within the same farm over time (2016–2019), as well as across participating farms.

Numerous parameters related to cow health in dairy systems have been benchmarked, including cow longevity, mastitis, lameness, milk production, milk quality, reproductive efficiency, and metabolic disease (7–11). It is common to benchmark dairies across multiple measures to provide as accurate and complete picture of these complex processes as possible. It has been stated that “it is advisable to use different benchmarks in combination for monitoring health, as well as for deciding on strategies to improve overall herd health management” (9); these authors, in a paper utilizing the Austrian dairy data collection system, concluded that “single parameters are not sufficient to evaluate complex parameters, such as fertility, udder health, or metabolic health.” Due to the multitude of factors that can contribute to antimicrobial effectiveness and resistance development, we argue that single parameters are not sufficient for evaluating antimicrobial use.

Our main objective was to develop antimicrobial use benchmark reports using metrics that provide veterinarians and animal caretakers with indicators of antimicrobial use within the context of recorded disease therapies as well as the outcome of these therapies within and across farms. This allows for a more nuanced interpretation of measures rather than indicating a simple increase or decrease of a single value, providing a more accurate and actionable tool to be used at the farm level to drive antimicrobial stewardship decisions.

## Materials and methods

### Data collection and analysis

Data for the calendar years 2016–2019 were collected from a convenience sample of 27 dairy farms in the United States. Herd size based on inventory of adult cows (>0 lactations) ranged from 211 to 6,676 cows, with a farm mean of 1,195 and a median of 952 (lactating and dry cows combined). All herds were Grade A farms (farms meet quality standards to market fluid milk in the United States), and none marketed organic product. Breed was predominantly Holstein, but two farms had 100% Jersey, and 5 others had <100% Holstein with considerable variation in non-Holstein percentage. All dairies used parlors for milk harvesting. Dairies were recruited through their veterinarians with whom the investigators had a previously established relationship. Efforts were made to include dairies in multiple regions of the United States (West, Midwest, Northeast); however, there were no restrictions for participation other than provision of data, a willingness to work with the investigators, and allowing publication of summarized data, while providing for confidentiality of individual farm-level data. Collection of data was accomplished at yearly farm visits by the investigators where a farm software data backup and collection of any non-electronic treatment records were conducted. These data were then submitted to standardization and quality assurance protocols as previously described (12), by standardizing record format (condensed vs. long), disease, treatment, dose.

In this report 4 farms were selected as examples which are representative of variation encountered in both cow health management and record keeping practices. Referred to as “Red Dairy,” “Cyan Dairy,” “Blue Farm,” and “Yellow Dairy,” the identity of these real farms has been masked. The farms selected as examples demonstrate a variety of different antimicrobial use patterns and context surrounding their antimicrobial use measures.

### Fundamental constructs: Therapeutic events and standardized treatment regimens

There are 2 constructs fundamental to the values reported: therapeutic events (TE) and standardized treatment regimens (REG). They are hierarchical, with REG nested within TE.

As previously described, REGs were defined by grouping treatment records by individual cow, disease syndrome, and active drug substance, where the time between product administrations was not >7 days (12).

Therapeutic events were identified by grouping regimens only by individual cow and date with the same 7-day maximum between one regimen and another; neither disease nor active substance was utilized a basis for grouping. Therefore, a single therapeutic event may contain multiple REG, both antimicrobial-containing regimens and regimens without antimicrobials, and be associated with a single disease or multiple diseases. When there are multiple standardized treatment regimens within one therapeutic event, the time frame of each can overlap in any manner, or they can be consecutive provided that there is no gap in therapy (no regimen being administered) where final administration of one regimen is separated from the first administration of another by more than 7 days. In order to accurately report documented disease, non-antimicrobial regimens were included as part of therapeutic events. Examples of non-antimicrobial regimens include documentation of “no treat,” documentation of disease without documentation of treatment administered (“unknown”), and documentation of non-antimicrobial treatments (“non-antimicrobial”) such as flunixin meglumine and calcium. In order to efficiently identify unique sequences in the original data that belonged to the same REG or TE, a function was written in R to assign unique identifiers to the original data rows within the same REG or TE. Details of this function are available in Supplementary material 3.1.

This approach facilitated analysis in two ways. First, it allowed counting the number of unique REG and TE while grouping the data by desired variables such as dairy, disease syndrome, antimicrobial class, or calendar year. These counts could then be put over any desired denominator, such as counts of cow years. Secondly, a description (rather than a total count) of standardized treatment regimens [as published in Schrag et al. (12)] or therapeutic events can be produced. These summaries describe the distribution of different characteristics of each construct. Descriptions of REG may be of importance to those interested in defining doses to apply to antimicrobial sales data, or who have research questions about dose or duration. Descriptions of TE may be of particular interest at the farm level to identify differences in recording methods, protocol adherence, or further details about which drugs are included in therapies. This is particularly important when attributing antimicrobial use to common diseases. These descriptions are provided in the Supplementary material 1.

One additional metric, antimicrobial regimen to therapy ratio (RT-ratio), was calculated as an indicator of the frequency with which antimicrobial regimens were included in therapeutic events. Calculation is performed by dividing the number of antimicrobial regimens by the number of therapeutic events: (REG/TE) = RT-ratio.

### Therapeutic outcomes

Therapeutic outcomes were calculated for each therapeutic event. The outcome was evaluated at a maximum of 30 days after the final administration date in the sequence. For example, if the final administration date of ceftiofur HCl was January 1, the outcome was evaluated on January 31. There were 4 possible outcomes: Relapse, Died, Sold, and Success. An outcome of “Relapse” was assigned if the cow received another TE prior to day 30. “Success” was defined as the cow remaining in the herd at the time of outcome evaluation (day 30) without relapse. An outcome of “Sold” or “Died” was assigned if a “Sold” or “Died” event was detected for that cow prior to day 30. Reasons for the cow dying or being sold were not consistently recorded. Individual therapeutic events could only have a single outcome. Assignment of outcomes was accomplished by using a similar R function (details in Supplementary material 3.1) to the one which created unique groups for each therapeutic event, but where the time gap was defined as 30 days rather than 7.

### Therapeutic event sequences and disease syndromes

By definition, a TE can include multiple regimens and therefore, may have multiple disease syndromes tied to it in the original treatment record. When only one disease syndrome was associated with a therapeutic event, that disease syndrome was assigned. If multiple diseases were identified as belonging to a single TE, then the disease syndrome associated with that sequence was defined as “complex disease.” An exception to this was made if there were two disease syndromes and one was “unknown.” In this case the known disease syndrome was assigned as the identified disease syndrome. More details about which disease combinations were common in complex disease can be found in the Supplementary material 1.3.

### Denominator of cow years

The denominator of cow years (CY) was calculated from the average inventory of adult cows present on each dairy during a calendar year. In the dairy record systems, CY is an average count of cows who have a lactation number greater than zero measured at multiple time points throughout the year. The method of obtaining this count varied slightly based on the type of production records available. For farms that utilized Dairy Comp 305 systems (26/29 herds; Valley Agricultural Software, Tulare, CA), CY was calculated as a weighted weekly average of the farm inventory of adult cows (LACT>0) for the given year. For all other farms, it was calculated as a weighted average of the farm inventory on Dairy Herd Improvement Association (DHIA) test days which varied from 6 to 12 times per year.

### Reporting format—Main questions addressed

All outputs from these analyses were formatted with the goal of providing information important for the evaluation and improvement of farm level antimicrobial stewardship. All visualizations were created using the ggplot2 package in R (13).

Seven specific questions that could be addressed with these data include:

A) How much disease is recorded? (TE/100CY)
B) How much antimicrobial use is recorded? (REG/100CY)
C) What are the outcomes of therapy? (%Success, %Relapse, %Sold, %Died)
D) How often are antimicrobials included as part of therapy? (RT-ratio)
E) How might recording practices be influencing results?
F) What variation exists across farms, or within a farm over time?
  In addition to these farm level questions, broader reporting at the national or commodity level was briefly explored:
G) What variation exists across years when data are summarized?

#### Scatter plots

**Figure 1:**
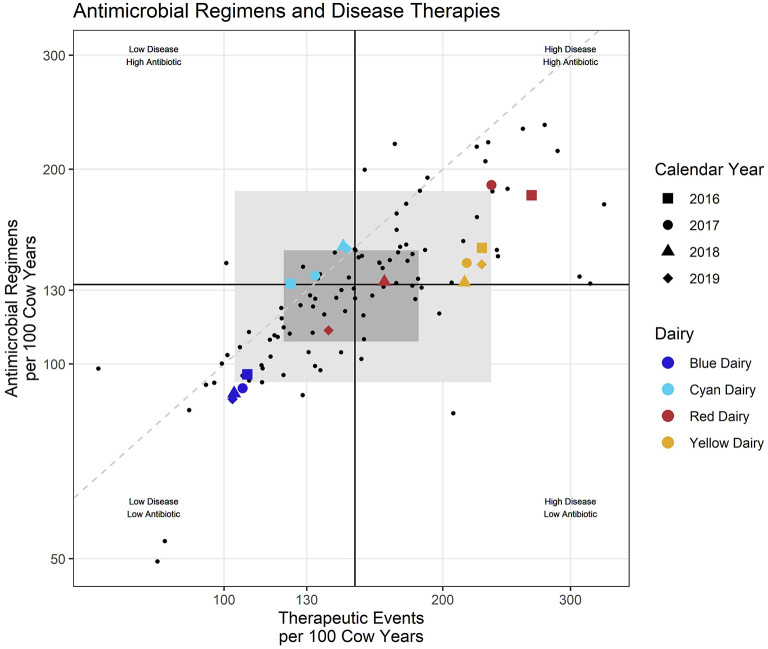
Each small dot represents an individual dairy during one calendar year/Colored shapes represent selected example daries with shape indicating calendar year. Black lines represent the intersect of the medians (middle value) for the entire dataset. Dashed diagonal line provides visual reference of a 1:1 relationship between the x and y axis. Shaded rectangles represent the middle 50% (dark) and 80% (lighter) of participating dairies all years combined.

Scatter plots of the rates of Antimicrobial Regimens (REG/100CY) by Therapeutic Events (TE/100CY) were created using the data from all farms. The axis scales were log transformed to facilitate visualization of points. Each small black dot in Figure 1 represents one calendar year from 2016 to 2019 for each dairy in the study. Each colored dot represents one calendar year from each example farm. The solid black lines creating crosshairs near the center of the graph, represent the median value (middle dairy) on each axis for all years and all dairies combined. This creates 4 quadrants each representing a different combination of values on each axis, with “high” and “low” not representing a judgment on appropriateness of use, but rather being above or below the median:

• Upper Left
  Low Disease (Therapeutic Event Rate),
  High Antimicrobial Use (Antimicrobial Regimen Rate)
• Upper Right
  High Disease (Therapeutic Event Rate)
  High Antimicrobial Use (Antimicrobial Regimen Rate)
• Lower Left
  Low Disease (Therapeutic Event Rate)
  Low Antimicrobial Use (Antimicrobial Regimen Rate)
• Lower Right
  High Disease (Therapeutic Event Rate)
  Low Antimicrobial Use (Antimicrobial Regimen Rate)

The dark gray shaded region in the background represents the area of the middle 50% (25–75th percentile) of the values on each axis. The light gray shaded region represents the area of the middle 80% on each axis. A dashed line was added for visual reference only and represents a 1:1 ratio between the x and y axis (RT-ratio = 1).

**Figure 2:**
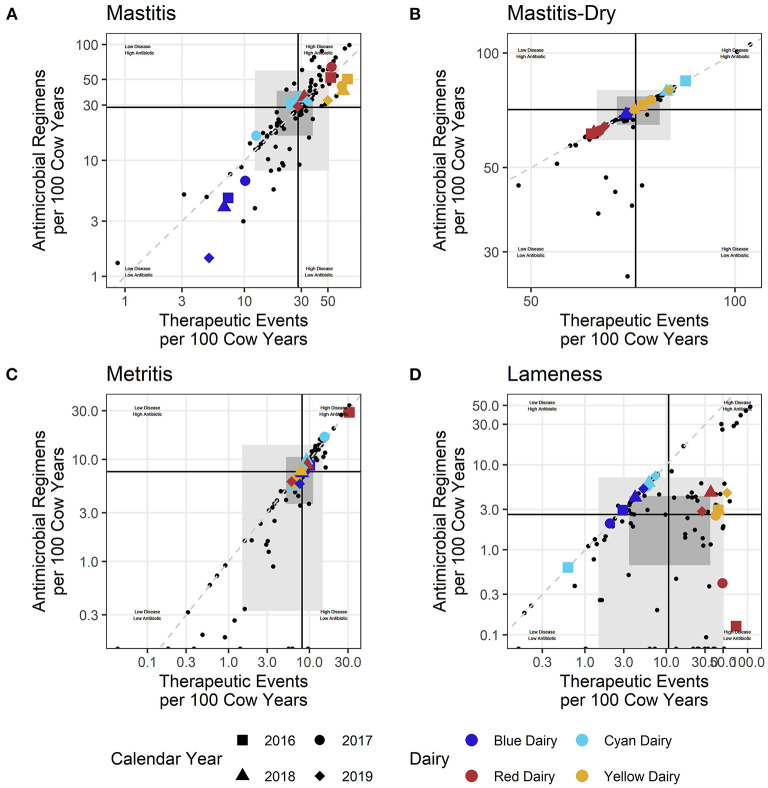
**(A–D)** Each small dot represents an individual dairy during one calander year/Shaded rectangles represent the middle 50% (dark) and 80% (lighter) of participating dairies. Black lines represent the intersect of the medians (middle value) for the entire dataset. Colored shapes indicate the value of example dairies and respective calendar years.

When individual farm reports were created, the reported farm's points were enlarged, shape was mapped to year, and color was mapped to the percent success for that year. Examples of individual farm benchmarks are presented in Supplementary material 5. This manuscript focuses on reporting formats generated for a veterinarian, or veterinary clinic, where multiple farms are presented in the same report. In this case rather than mapping color to percent success, color is mapped to a unique farm identifier (Red Dairy, Blue Dairy, Cyan Dairy, Yellow Dairy as examples of 4 farms for a report to a veterinary practice), and percent success is only reported in the tabular format rather than the scatter plot. In addition to a summary plot where all diseases are combined (Figure 1), scatter plots are also created for each disease individually (Figure 2). A more graphical explanation of scatter plots can be found in Supplementary material 4.1.

**Figure 3:**
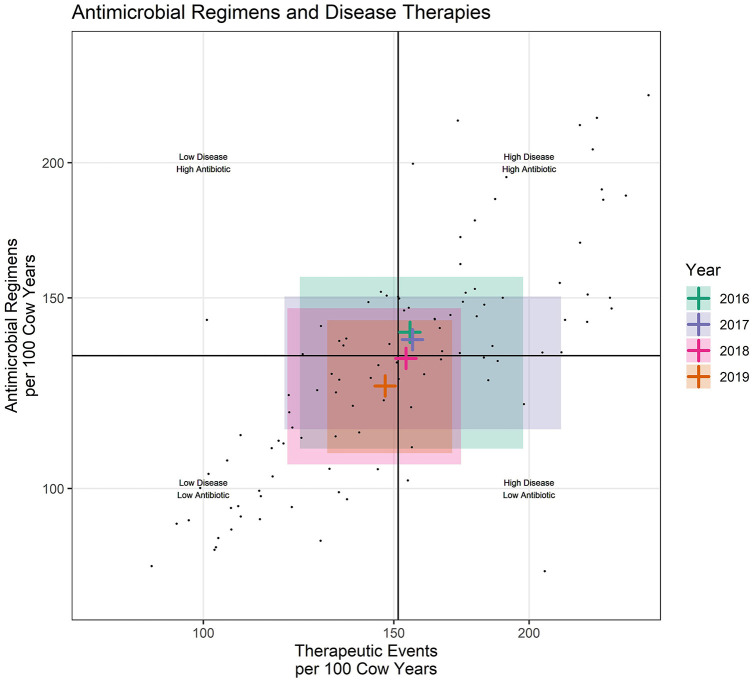
Each small dot represents an individual dairy during one calendar year. Shaded rectangles represent the middle 50% of participating dairies for each calendar year. Black lines represent the intersect of the medians (middle value) for the entire dataset. Colored crosshairs represent the intersect of the median (middle value) for each year.

When used for multi-farm reporting, scatter plots inform main questions A, B, and F. When used for individual farm reporting, they additionally inform main question C. Scatter plots can also be used to summarize data across broader categories such as for use across all dairies by year (Figure 3). When utilized in this manner they inform main question G, and the shaded areas are generated by summarizing at the year level and mapping the color of the shaded region and median cross hairs to year.

#### Tabular format and recording details

All 11 metrics calculated are presented in 3 sets of tables reporting how much disease and antimicrobial use is reported, the outcome of therapy, and what data are recorded. Each table is subdivided by disease and calendar year. The numerical values within the tables are the value for the individual farm reported. The background color of each square is shaded according to where that value ranks across the rest of the farms in the data set for each metric. This ‘rank' is referred to as “Percentile Rank” throughout the reports. It is calculated by grouping values by metric, and then utilizing the percent_rank()^1^ function in base R to assign a percentile rank to each value in the data set. Classifications were then defined as “very low” (0-≤20th percentile), “low” (>20-40^th^ percentile), “average” (>40-60^th^ percentile), “high” (>60-80^th^ percentile), or “very high” (>80^th^ percentile). Each time these percentile rank classifications are utilized in the benchmarks they are each tied to a corresponding background color in the tabular format, or point color in the individual farm scatterplots.

**Figure 4:**
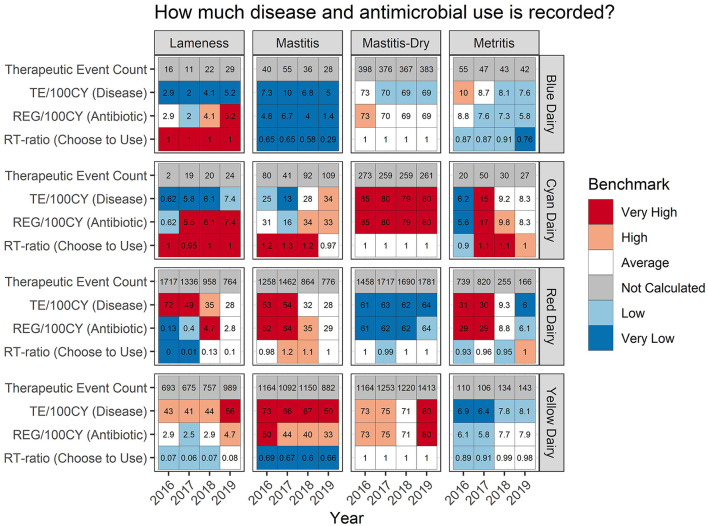
For each measure listed on left the value is reported numerically within the cell. The background color indicates how that value ranks compared to other participating dairies.

**Figure 5:**
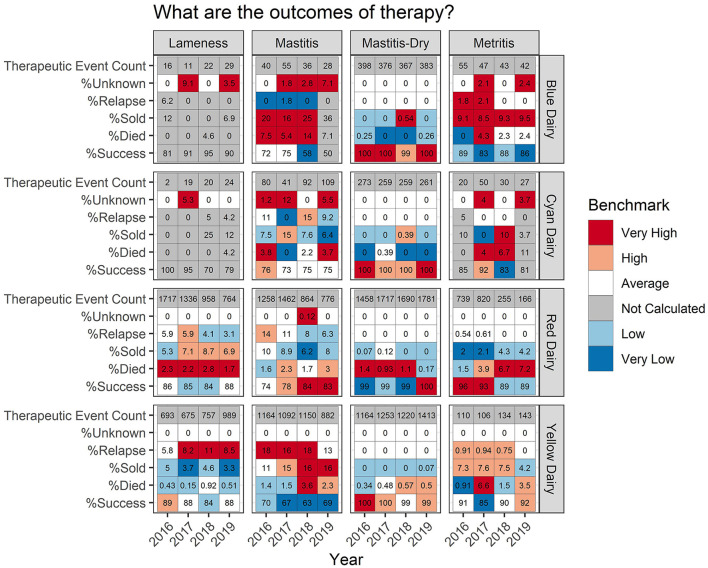
For each measure listed on left the value is reported numerically within the cell. The background color indicates how that value ranks compared to other participating dairies. Occasionally outcomes could not be accurately determined due to/data quality issues associated with cow identity. This occurred more frequently for dairies with hand written treatment records i.e., Blue Dairy and Cyan Dairy.

**Figure 6:**
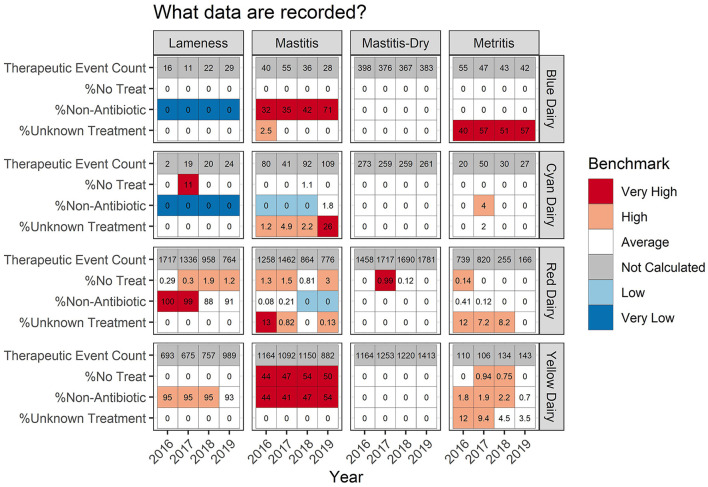
For each measure listed on left the value is reported numerically within the cell. The background color indicates how that value ranks compared to other participating dairies. The measures listed in this table are not mutually exclusive and therefore do not sum to 100% within dairy/year.

Some farms had extremely low incidence (<30 total TE) within a disease/year category. In these cases, calculating a percent success and a percentile rank was not interpretable due to the low counts. This is indicated by a gray color in the background (listed as “not calculated” in the legend) of the tabular output (Figures 4–**6**) and warns that the count was low and the value should be interpreted carefully.

## Results

The figures in this manuscript represent examples of the final report format generated iteratively over the 4 years of the study. This final format was arrived at after revising the benchmark reports each year based on discussions with the participating farm's veterinarians and animal health teams. The report format presented in the main manuscript is aimed at veterinarians or animal health teams with the need to efficiently evaluate multiple farms. The example farms presented are: Red Dairy, Blue Dairy, Cyan Dairy, and Yellow Dairy. These example farms are each an actual farm in the study, renamed to protect anonymity. Section Discussion of the supplemental materials contains additional example reports for these farms where the individual farm is the primary audience.

### Scatter plots

Scatter plots allow efficient evaluation of REG/100CY within the context of TE/100CY. The visual aids in the graph can be used to rapidly categorize and compare farms according to both their magnitude of recorded disease pressure and antimicrobial regimens used. For example, in Figure 1, both Red Dairy and Cyan Dairy fall within the middle 50% of dairies (dark gray shaded region) for some or all study years. Yellow Dairy falls within the middle 80% (light gray) region for all years, as does Blue Dairy for 2016. For years 2017–2019 Blue Dairy falls outside (below and left of) all gray shading indicating that they are below the 10^th^ percentile for values on both axes. Likewise Red Dairy falls outside (above and right of) the gray shading indicating that compared to other study dairies for 2016 and 2017 their values were above the 90^th^ percentile.

Dairies and their veterinarians can also observe the magnitude of variation in their metrics across years. For example, both Blue Dairy and Yellow Dairy show little variation between years, while Cyan Dairy shows somewhat more variation across calendar years. Red Dairy shows the most variation with 2016 and 2017 far above the gray middle 80% region, 2018 is still in this quadrant but well within the shaded middle 50% region, and 2019 enters the low disease—-low antimicrobial use quadrant but remains within the middle 50%. However, this is very difficult to interpret with all diseases combined.

Similar observations can be made for each disease individually (Figure 2). For each disease, questions can be asked about why there are changes within a dairy over time, why a dairy falls within a particular quadrant, or why its values are on the edges of the distribution of farms (outside the gray shading). To simplify discussion, only 4 diseases were selected for inclusion in this manuscript: mastitis, dry cow treatment (mastitis-dry), lameness, and uterine disease (metritis). However, 9 disease syndromes were reported in the individual dairy benchmarks which can be found in the Supplementary material 5.

### Broad data summaries

While this study primarily focused on farm level reporting, the same measures useful for farm level reporting might also be utilized for fulfilling reporting requirements at the market chain or national level. For example, scatter plots can be created by summarizing values from all dairies within year (Figure 3). When data are summarized by year, there are now 4 cross hairs created by the median values on each axis calculated for each calendar year. These cross hairs indicate the midpoint of the distribution of values within each calendar year. The shaded area represents the middle 50% of values for each year, creating a reporting format where antimicrobial use can be broadly reported across years within the context of all diseases. This informs a fundamental goal of stewardship, reduction in the need for antimicrobials (as indicated by TE/100CY), to be presented along with the antimicrobial use (REG/100CY). Figure 3 is included in this manuscript only to demonstrate possible methods for broad level reporting. No attempt should be made to interpret trends over time in U.S. dairies from this small convenience sample.

### Frequency of antimicrobial inclusion

There are two ways to evaluate how often antimicrobials are included in a therapeutic event. The first is by observation of where a point falls in relation to the dashed line in the scatter plots. For example, in the mastitis plot (Figure 2A) Blue Dairy and Yellow Dairy fall below the dashed line, indicating that on average not all mastitis TE include an antimicrobial. Red Dairy and Cyan Dairy fall above this line, indicating that for those farms, on average, every mastitis therapeutic event includes at least one antimicrobial, and some include more than one. Farms falling far below the dashed line (a 1:1 RT-ratio) compared to farms falling very near to it are sometimes making different decisions about when or how they include antimicrobials in their treatment protocols. For example, some farms are using pathogen-based treatment protocols (Blue Dairy and Yellow Dairy) where only certain mastitis cases receive antimicrobial therapy and the farms fall well below the dashed line in Figure 2A. For each disease in Figure 2, the location of the cross hairs in relation to the dashed line indicates the median RT-ratio for each disease. For mastitis, mastitis-dry, and metritis the cross hairs fall very near 1. For lameness the crosshairs fall well below the dashed line indicating that not all therapies for lameness include an antimicrobial. Here Red Dairy and Yellow Dairy record all lameness events, while Blue Dairy and Cyan Dairy record only those animals treated with an antimicrobial. When interpreting the scatter plots, care must be taken to recognize the influence that styles of record keeping might have on observed differences across farms.

### RT-ratio

A second way to evaluate how often antimicrobials are included is indicated by the RT-ratio in the tabular format (Figure 4, row 4 for each dairy). It indicates the frequency with which antimicrobials were chosen for inclusion in a therapeutic sequence for each disease syndrome. For mastitis both Blue Dairy and Yellow Dairy have very low RT-ratios ranging from 0.29 to 0.69, as indicated by the blue shading in the background of their tabular format. Both of these dairies were confirmed to be using pathogen-based treatment strategies. For mastitis, Red Dairy and Cyan Dairy had RT-ratios ranging from 0.98 to 1.32. Neither of these farms were utilizing pathogen-based treatment protocols.

### Therapeutic outcomes

Figure 5 shows the percent therapeutic success, and reasons a therapeutic event failed, the cow died (% Died), the cow relapsed (% Relapse), or the cow was culled (% Sold). Focusing on the % success for mastitis, Red Dairy has a success of 74, 78, 84, and 83% for years 2016–19, respectively. The background color indicates how Red Dairy ranks compared to other study farms. Background values range from white indicating average rank in 2016, to light red indicating high rank in 2017, to dark red indicating a very high rank in 2018 and 2019. The background color can be used to rapidly and comparatively access the performance of the other example farms for mastitis % success (the bottom row for each dairy under the mastitis heading). Yellow Dairy has the lowest mastitis % success across all years: light blue 70% in 2016, and dark blue 67%, 63%, 69% in 2017–19, respectively. When examining the details of therapy failures on Yellow Dairy, it can be observed that the low % success can be attributed to both relapses (% Relapse) and culling decisions (% Sold). This is in contrast to Blue Dairy where relapses are very uncommon (dark blue background for % Relapse), but the % Died ranks very high compared to other dairies for 2016–2018.

An example of a pattern which may be worth investigating further can be observed in the metritis % Success for Red Dairy. In 2016 and 2017 it was very high, 96% and 93%, respectively. However, in 2017 and 2018 it was lower, at 89%. While these seem like small changes numerically, they are relatively large shifts (top quintile vs. second to bottom quintile) in where this dairy lies within the distribution of success for metritis across all farms and all years. There are many potential drivers of this, both within this farm and related to changes that may have happened on other farms contributing to the distribution. Figure 4 adds more context for interpretation, indicating that the disease incidence was very high in 2016-17 (31 and 30 TE/100CY, red background), average in 2018 (9.3 TE/100CY, white background), and very low in 2019 (6 TE/100CY, dark blue background). However, interpretation requires extensive farm level knowledge.

Occasionally an outcome could not be calculated for a therapeutic event. These are reported as “Unknown” outcomes. This occurred more frequently for the dairies with handwritten treatment records (Blue Dairy and Yellow Dairy) where data quality did not always allow for reliable matching of a therapeutic event to an outcome.

### Recording practices

Presenting data in tabular format provides several other details (Figure 6) which aid in interpretation of measures or trends. Continuing with the example of Red Dairy's metritis therapy, Figure 6 indicates that in 2016–2018, 8–10% of metritis therapies were documented as unknown, but in 2019, 0% of therapies were documented as unknown. If this shift occurred because record keeping practices became more complete, and those therapies which previously failed to document the specific therapy provided now specify it, then this change would not influence the overall TE/100CY, but it might influence the REG/100CY. However, if instead this farm had a serious labor shortage and recording practices were revised to reduce the amount of employee time devoted to recording causing these once documented but incomplete records to simply be never documented, this could decrease the TE/100CY in the records artificially. Alternatively, if a farm previously never documented retained placenta events that received no therapy, and then revised their protocol to begin recording them, this might increase their apparent TE/100CY even though the farm level incidence remained stable.

Lameness recording practices are variable across dairies (Figure 6). For Blue Dairy and Cyan Dairy, zero percent of recorded lameness therapies included a non-antimicrobial. However, on Red Dairy and Yellow Dairy >88% of lameness therapies indicated that a non-antimicrobial was used. This information should be related back to the disease specific scatter plots (Figure 2D) where there are 2 distinct populations of dairies, those who fall very near to the dashed line (like Blue Dairy and Cyan Dairy) and those who fall below and to the right of it (Red Dairy and Yellow Dairy). Proper interpretation of changes within a farm over time, or comparisons across farms must acknowledge the context of recording practices and its influence on a farm's values.

## Discussion

Several studies have compared antimicrobial use metrics specifically within dairy systems. While there are some nuances across metrics which might relate to different stewardship program priorities, the differences are slight with the exception of measuring mass-based grams compared to a dose-based measure of treatment incidence (14–16). In a study looking at benchmarking both large and small animal clinicians in the United States it was found that “Prescribing frequency, durations of therapy, and ranking of antimicrobial classes appear to be the metrics most well-received by veterinary clinicians, while dose-based metrics associated with the ADD [animal daily dose] are less intuitive.” (17); even in a hospital setting with clinicians interpreting the benchmarks, dose-based metrics were perceived as “less intuitive.”

The selection of antimicrobial use metrics in this manuscript was driven by consideration of 5 basic steps in antimicrobial stewardship: (1) characterization of disease pressure through appropriate case definitions and diagnostics, (2) consideration of alternatives to antimicrobials for prevention, control, and treatment of disease, (3) when necessary, selection of the appropriate antimicrobial regimen for each disease, (4) continuous monitoring of antimicrobial use and therapeutic outcomes, and (5) continuous re-evaluation of the need for the instituted antimicrobial regimens. The reporting format was guided by a quest to find efficient methods for generating more specific and actionable questions about antimicrobial stewardship on an individual farm.

The consideration of antimicrobial stewardship step 1 drove selection of REG/100CY and TE/100CY based on their direct and intuitive relationship to the component of detected disease (12). In this context, detected disease may lead to either therapy of acute disease or the use of an antimicrobial for prevention or control. The link between recorded disease pressure and recorded antimicrobial use also allowed the reporting of the RT-ratio, which provides information about the decision that an antimicrobial intervention is necessary, the third step in antimicrobial stewardship.

In a recent and comprehensive review of behavior associated with dairy antimicrobial use, one of the recommendations was to improve “ability and confidence to implement prudent AMU practices while maintaining animal-welfare standards” (18). Reporting therapy outcomes provides at least some context of animal welfare implications for changes in antimicrobial use. The simultaneous monitoring of outcomes along with use could provide needed confidence for stakeholders to make changes in use patterns by demonstrating that outcomes are important and are being tracked along with antimicrobial use. Additionally, it sets a precedent that true stewardship means use optimization first by disease incidence reduction, combined with optimizing case definitions for antimicrobial treatment. This should all occur within the context of therapeutic outcomes. Consideration of outcomes supports antimicrobial stewardship step 5. However, the authors wish to make it clear that outcomes determined in this manner should not be construed as representing the effect of the interventions in place. Rather, they may be considered in light of other inputs such as clinical trial outcomes.

Additionally, outcomes provide context for interpreting TE/100CY by offering some additional information which may drive questions about case definitions or diagnosis. For example, consider that mastitis on Red Dairy falls high into the upper right quadrant in years 2016 and 2017, indicating that both TE/100CY and REG/100CY were high compared to other study dairies (Figure 1, and dark red background in Figure 4). However, they fall well into the central gray zone in 2018 and 2019. Did they decrease both antimicrobial use and disease incidence by simply failing to detect or record disease? Their treatment outcomes in Figure 5 indicate that this is not likely to be the case. Their % success actually improved in 2018 and 2019 compared to previous years. This would decrease the likelihood that previously detected disease is now being ignored, and indicate that some other variable changed, such as improved disease prevention with better overall cow immune status, or decreased disease pathogenicity of endemic pathogens (e.g., eradication of *Prototheca*?).

Blue Dairy falls on the opposite extreme with all years falling into the lower left quadrant of the scatter plot (Figure 1). Their % Success for mastitis therapy is average in 2016-17, very low in 2018 and not ranked in 2019. Do they have low disease and low use because they are ignoring or misclassifying disease? From these benchmarks alone it is impossible to determine whether this low TE/100CY and low REG/100CY are the result of very good management or very poor management. It is good management if the farm is doing a great job at disease prevention, and simply has average to low % Success because of high culling pressure for mastitis cows on this farm. It is poor management if they are simply ignoring or misclassifying disease, and therefore treating few animals very late. This would give them low TE/100CY and low REG/100CY, but poor outcomes because only the most severely affected animals are documented in the record. This is yet another example of appropriate and useful interpretation of comparative metrics between farms only being possible by investigation of reasons for the differences at the farm level.

One of the primary limitations of the benchmark metrics proposed here is that the 30-day outcomes are relatively crude measurements. Ideally, reports should offer more granular outcomes such as outcomes related to milk production and/or quality (e.g., somatic cell counts), or 120-day pregnancy rates. Although such outcomes are more complex to calculate and were well-beyond the scope of this project, their inclusion as context for antimicrobial use should be further explored. The authors strongly recommend that production efficiency measures are included in any large-scale program which benchmarks antimicrobial use, and that evolutions of existing benchmarking programs include this context.

Although others have evaluated the role treatment threshold (case definition) might play in antimicrobial stewardship (19), to the author's knowledge this study is the first to utilize detected disease as a denominator for antimicrobial use in dairy cattle. It was not described as a denominator in any of the 12 benchmarking programs described within the AACTING review paper (5). In the work reported in this manuscript, it was quickly discovered that dairies who utilized pathogen-based treatment programs could not be easily differentiated using only the standard denominators such as Cow Years or kilograms of animal treated. This challenge drove the development of the “dashed line” in the scatter plots, and the RT-ratio. Both comparison of antimicrobial REG to total TE as the RT-ratio (REG/TE) and the distance a dairy is from the dashed line in a scatter plot represent the compilation of multiple therapeutic decisions to use or not use an antimicrobial for a particular disease therapy (TE).

For example, the scatter plot of dry cow treatment (mastitis-dry, Figure 2B) demonstrates cross hairs (intersect of x and y axis medians) very near the diagonal dashed line (representing an RT-ratio of 1:1). When considering all study farms, most are very tightly grouped on this line since most farms use “blanket” dry cow therapy where an antimicrobial is included for every therapeutic event. However, there are a few (small black dots) falling far below the dashed line. Most of the dairies with very low RT-ratios were confirmed to be utilizing selective dry cow therapy rather than blanket dry cow strategies. For selective dry cow therapies, a portion of the therapeutic event included only a non-antimicrobial teat sealant rather than both a sealant and an antimicrobial or just an antimicrobial.

Large shifts in variation of a metric should raise questions about potential changes in case definitions, case management, recording practices, or disease prevention strategies. Presenting all metrics on one page in tabular format (Figures 4–6) is an attempt to provide as much context as possible for all metrics to highlight differences in case definitions or recording practices. When all information is interpreted within its context the tabular outputs are helpful to answer some initial questions generated by the scatter plots such as:

- Was low success due to a higher cull rate or more relapses?
- Is disease level classified as high just because records are more complete (i.e., “are no treats” recorded)?
- How might case definition be affecting the amount of disease reported?

Although many details are included in these tabular outputs, “boots on the ground” knowledge of farm activities is still necessary for accurate interpretations.

Figure 1 presents an example of a large shift in values for Red Dairy. This was investigated and confirmed that a significant management change occurred between years 2017 and 2018. In 2016 and 2017, there were 3 locations, each managed separately. In 2018, these locations were combined so that all fresh cows were managed at a single location. Therefore, the observed change across years was due to changes in disease incidence driven by management change, change in case definitions driven by personnel change, therapeutic decisions driven by a change in how responsibilities were allocated, and only minor changes in record keeping practices. Recognition that interpretation of each farms data requires farm level knowledge of recording practices is essential to gaining accurate incites about antimicrobial stewardship.

As demonstrated in the lameness plot (Figure 2C), sometimes observed differences are caused by differences in recording practices (i.e., recording all lameness events vs. only those treated with an antimicrobial) rather than fundamental differences in treatment protocols. Pursuing a goal of completely standardized recording practices across all farms is unrealistic. However, efforts to achieve some basic health record keeping standards are needed for farms to gain actionable insights from their health data. Examples of basic standardization include recording all disease events not just those treated with antimicrobials, and associating treatments with a disease or condition (applying a case definition) rather than only listing the drug used for treatment.

In addition to differences in recording practices, there is also farm-to-farm variation in case definitions. For some farms, the case definition of mastitis is observation of a few “flakes” in the milk or the cow tests positive on a California Mastitis Test, while the case definition of mastitis on other farms is not met until milk is significantly abnormal for multiple consecutive days. This can easily lead to long detailed debates on the most appropriate case definitions. However, that is not the goal. The main goal is that the reporting format allows for detection of changes over time, reported across multiple metrics which allows evaluation of an individual farm's progress by those familiar with what changes may have occurred.

While some might interpret the above-mentioned differences in case definitions and recording practices as detrimental, the authors contend that they are simply representative of current dairy systems and accurately describe current practices. It is unlikely that a single recording method and/or single case definition would be appropriate on every farm. Each farm has a unique set of personnel with unique skill sets, and a unique population of bacteria present on their dairy. Identification of these differences is one of the opportunities associated with this style of reporting. Reports intended to support antimicrobial stewardship applications should elicit questions of “why is…?” rather than produce statements pronouncing a particular dairy falling above or below a particular (arbitrary) target value. Interpretation of measures with as much context as possible provides more information to those actively involved in defining and providing therapy to sick animals. This offers the potential to identify multiple opportunities which can lead to a more holistic approach to antimicrobial stewardship rather than simple reduction of the use metric without the context of the associated disease incidence or therapy outcome.

Some have suggested that farm level sales data should be the gold standard for antimicrobial use measurements, as use measured by farm records has shown to underestimate use measured by sales data (20). While sales data may more accurately account for the sale of each mg of drug distributed to a farm, the authors of this manuscript believe that the farm treatment records provide more utility in identifying actionable opportunities for changes in disease diagnosis or treatment regimens. The context provided by the treatment record is necessary to direct investigations as to the appropriateness of the antimicrobial use, to identify actionable changes to improve use, and to keep track of therapeutic outcomes subsequent to alterations in antimicrobial use. Ignoring this context makes it more difficult to identify actionable changes and fails to provide for monitoring of both antimicrobial use and treatment outcomes subsequent to changes in antimicrobial use. Although documented changes in REG/100CY, TE/100CY, and RT-ratios do not directly indicate why the change occurred, efficient reporting mechanisms to observe these changes can drive reasonable and in citeful questions leading to meaningful investigations of cause.

An absolute requirement for the appropriate use of antimicrobial use metrics and benchmarks is that the interpreter is acutely aware that selected metrics describe “what happened” (with varying degrees of accuracy) and not “why it happened.” Even when there is intimate knowledge of farms being evaluated by veterinarians and their clients, there are unknown factors affecting peer farms being used for comparison which are equally as important to understand, highlighting the need for a central resource which is able to further investigate when unexpected patterns or distributions of farm values occur. Developing regulatory policy, legislative initiatives, or marketing programs based on poor understanding of inadequate metrics has the potential to cause harm to animal welfare and efficiency of production with minimal to no improvement in antimicrobial resistance selection pressure.

In an effort to remain consistent with benchmarking other complex processes within the dairy industry and to accurately represent reality, this benchmarking system utilizes multiple measures of antimicrobial use. When reporting benchmarks back to the participating farms and veterinarians, the early reporting format with simple metrics required little explanation, but participants struggled to know what to do with the measures and were quick to mention that they might have more disease or question if they were more successful with their therapies. The more complex reports, as presented here, required some amount of training for interpretation but generated more engagement and excitement about having access to the benchmarks. It should be noted that some antimicrobial use monitoring systems disagree with the approach and recommend that “It might be advisable not to benchmark too many aspects, as multiple benchmarking results for a single species (category) might become confusing and end up being counterproductive, especially if the results appear contradictory.” While recognizing that no single metric was sufficient, Craig et al. suggested that a single metric should be chosen to simplify the communication of the measure (21). We disagree with this approach, instead recommending that when results appear contradictory studies should be conducted to evaluate which actions might be most effective at improving antimicrobial stewardship. There is valuable information in determining why two metrics give different pictures of the same systems. Forcing a simplistic single numerical metric for antimicrobial use is an approach which may increase the simplicity of detached decision-making, but also increases the potential that the detached decisions are precisely wrong.

## Conclusion

This study demonstrates that existing treatment records can be utilized to provide antimicrobial use reports containing multiple measures presented within the context of disease pressure and therapeutic outcomes. The authors are optimistic that if further advances are made in data interoperability, a similar system of reporting could be scaled up to include a larger population of dairies. Because the benchmark system outlined in this manuscript is detailed and relates directly to farm level disease management practices, there is hope that the described data structure might provide a starting point for identification of future opportunities related to the development of antimicrobial stewardship tools. If broad level antimicrobial use summaries are created at a commodity or national level, adding a description of disease pressure (despite the challenges) could allow a more contextual interpretation of antimicrobial use data.

## Nomenclature

### Construct definitions

**Standardized treatment regimen (REG)** – a standardized regimen refers to a treatment or group of treatments administered consecutively to an individual animal where the gap between administrations is never >7 days. A REG is drug (active substance) and route specific. A detailed definition of a REG can be found in Schrag et al. (12) (see Supplementary material 1.1 for more details).

**Therapeutic event** (**TE**) – A group of REGs where the time gap between the last administration of one REG and the first administration of another is never >7 days. These regimens may address a single disease event. A TE may also span multiple disease events (e.g., mastitis followed by metritis) if these diseases are identified in the same cow with a 7 day or less gap between regimens associated with each disease. When a TE contains multiple types of diseases it is classified as a “complex disease” therapeutic event (see Supplementary materials 1.2, 1.3 for more details).

**Outcome** – The outcome (Success, Sold, Died, Relapse) associated with each therapeutic event, evaluated at a maximum of 30 days after the final administration of the last REG. If used in a numeric context it is the percent of Therapeutic Events resulting in a particular outcome, i.e., % Success.

### Mathematical summaries of construct frequencies

**REG/100CY –** numeric count of the number of *antimicrobial* regimens (REG) divided by the average yearly cow inventory (CY), then expressed in relation to 100 cow years.

**TE/100CY** – a numeric count of therapeutic events (TE) divided by the average yearly cow inventory (CY), then expressed in relation to 100 cow years. It is important to note that *all* therapeutic events count here, including ones which do not include an antimicrobial in any of the regimens.

**RT-ratio** – the ratio of count of antimicrobial regimens (REG) to count of therapeutic events (TE). This measure is an indicator of the frequency with which antimicrobials are included in therapy.

**% Success** – the number of therapeutic events resulting in an outcome of success divided by the total number of therapeutic events. This measure can be grouped by any number of categories but is usually reported by disease syndrome.

## Data availability statement

The datasets presented in this article are not readily available because the data used in this study belongs to the farms, and sharing the data in a non-summarized format was not part of the original agreement for the study. Requests to access the datasets should be directed to mapley@k-state.edu.

## Author contributions

NS, SG, and MA contributed to conception and design of the data collection. NS and DA organized the database and performed the analysis. NS wrote the first draft of the manuscript but relied heavily on all other authors to provide guidance within their areas of expertise. All authors contributed to the methods of data analysis, contributed to manuscript revision, read, and approved the submitted version.

## Funding

Funding for this project was made possible, in part, by the U.S. Food and Drug Administration through grant number U01FD005868 and U01FD005878. Views expressed do not necessarily reflect the official policies of the Department of Health and Human Services; nor does any mention of trade names, commercial practices, or organization imply endorsement by the United States Government.

## Conflict of interest

The authors declare that the research was conducted in the absence of any commercial or financial relationships that could be construed as a potential conflict of interest.

## Publisher's note

All claims expressed in this article are solely those of the authors and do not necessarily represent those of their affiliated organizations, or those of the publisher, the editors and the reviewers. Any product that may be evaluated in this article, or claim that may be made by its manufacturer, is not guaranteed or endorsed by the publisher.

## Author disclaimer

Views expressed do not necessarily reflect the official policies of the Department of Health and Human Services; nor does any mention of trade names, commercial practices, or organization imply endorsement by the United States Government.

## Footnotes

^1^Hadley Wickham, Romain François, Lionel Henry and Kirill Müller (2021). dplyr: A Grammar of Data Manipulation. R package version 1.0.6. https://CRAN.R-project.org/package=dplyr.
