# Supplementary material for: Improving farm-level antimicrobial stewardship benchmarks by reporting antimicrobial use within the context of both the magnitude of disease pressure and the outcome of therapy
Source: Front Vet Sci. 2022 Oct 5;9:1022557. doi: 10.3389/fvets.2022.1022557 (PMC9581275; doi:10.3389/fvets.2022.1022557)
Supplement: Supplementary file 1 [file Table_1.DOCX]

**Supplemental information**

Contents

[1. Construct descriptions 2](#_Toc95890457)

[1.1 Standardized treatment regimen descriptions 2](#_Toc95890458)

[1.2 Therapeutic event descriptions 9](#_Toc95890459)

[1.3 Complex disease details 17](#_Toc95890460)

[2. Relative contributions by disease as a scatter plot. 19](#_Toc95890461)

[3. R code to identify therapeutic events and treatment regimens 20](#_Toc95890462)

[4. Training figures for graphics 21](#_Toc95890463)

[4.1 Scatter plots – Figure description 22](#_Toc95890464)

[4.2 Scatter plots – Individual farm reports 29](#_Toc95890465)

[4.3 Scatter plots – Multiple farms 32](#_Toc95890466)

[4.3 Scatter plots – Commodity group or national summaries 34](#_Toc95890467)

[4.4 Tabular Output – Individual farm 35](#_Toc95890468)

[5. Example Farms - Original benchmark reports 37](#_Toc95890469)

[6. Summary of study antimicrobial use 46](#_Toc95890470)

[6.1 Use by disease 47](#_Toc95890471)

[6.2 Use by antimicrobial class 66](#_Toc95890472)

# 1. Construct descriptions

## 1.1 Standardized treatment regimen descriptions

Descriptions of standardized treatment regimens are presented below. Each descriptive figure describes the drug product administered within each route of administration (intramammary, intramammary-dry, other, and parenteral) with the percentage figure to the right representing percent (%) of total regimens described in the figure. The figures describe, in order, number of calendar days of product administration per regimen (CDoA), regimen time frame, total grams per regimen, and the mean grams of antimicrobial per CDoA per regimen. Time frame (TF) was calculated as the number of calendar days between the first and last administration. For drugs administered daily (or more frequently) the TF is always one day less than the CDoA for any given regimen. For drugs administered at intervals longer than 24 hours, the TF may be longer than the CDoA; i.e., if two doses of ceftiofur CFA are administered 72 hours apart the CDoA is 2 (one day for each calendar day the drug was administered), and the time frame is 3 (date of first administration to date of second administration).

Regimens of the same combination of route and drug product contributing less than 0.1% of the total regimens are not described here. However, if the drug product and route combination was used more frequently than this, all regimens for that route and drug product were reported. Therefore, there are several outlier regimens which are described. For example, there is a single intramammary ceftiofur HCL regimen which has 48 CDoA, but a time frame of only 5 days. This is impossible, but it is accurate according to the original data. Because the original data for this entry was listed in regimen format (i.e., Remark = LFSP6, see (Schrag et al., 2020)), the automated cleanup function calculated the final treatment date for each row as the original treatment date plus the number of administrations. In this case, because the cow was documented as being treated on 8 original rows of data, there are 48 CDoA, but the time frame is only 5 as the final date of the last administration was calculated by adding 5 days to the regimen start date. Data such as these are reported as is, without assumptions about validity because the rule in this analysis is to report all data rather than filtering out data based on assumptions of what was an entry error. Future work should be done specifying the tolerance for this type of outlier in the data clean up algorithm.

Another outlier in these data was the use of Ceftiofur HCL listed as “other” for route of administration. This was reported on 8 farms, where most instances were documented as intraocular pinkeye. Only 2 farms had more than 3 regimens over 4 years matching this description. On one farm there were 39 total regimens, documented to be ocular route of administration for pinkeye. The other farm had 261 administrations documented as use for metritis. This use occurred on only 20 individual calendar days spanning a 5 month time period. This use was very likely due to inaccurate documentation (accidental selection of a METR [metritis] event rather than MAST [mastitis] event). However, due to available time resources within this study we were not able to trace back all potentially inaccurate regimen descriptions such as these. It is appropriate to acknowledge that farm level data will have this type of variation present, and this should be considered in the design of monitoring programs.

For a description of how to read the violin plots below please refer to previously published figure S1 found in the supporting information of Schrag et al. <https://onlinelibrary.wiley.com/action/downloadSupplement?doi=10.1111%2Fzph.12766&file=zph12766-sup-0001-FigS1.jpg>

Schrag, N.F.D., Apley, M.D., Godden, S.M., Lubbers, B.V., and Singer, R.S. (2020). Antimicrobial use quantification in adult dairy cows - Part 1 - Standardized regimens as a method for describing antimicrobial use. *Zoonoses Public Health* 67 Suppl 1**,** 51-68. doi: 10.1111/zph.12766.

**Figure S1.1**


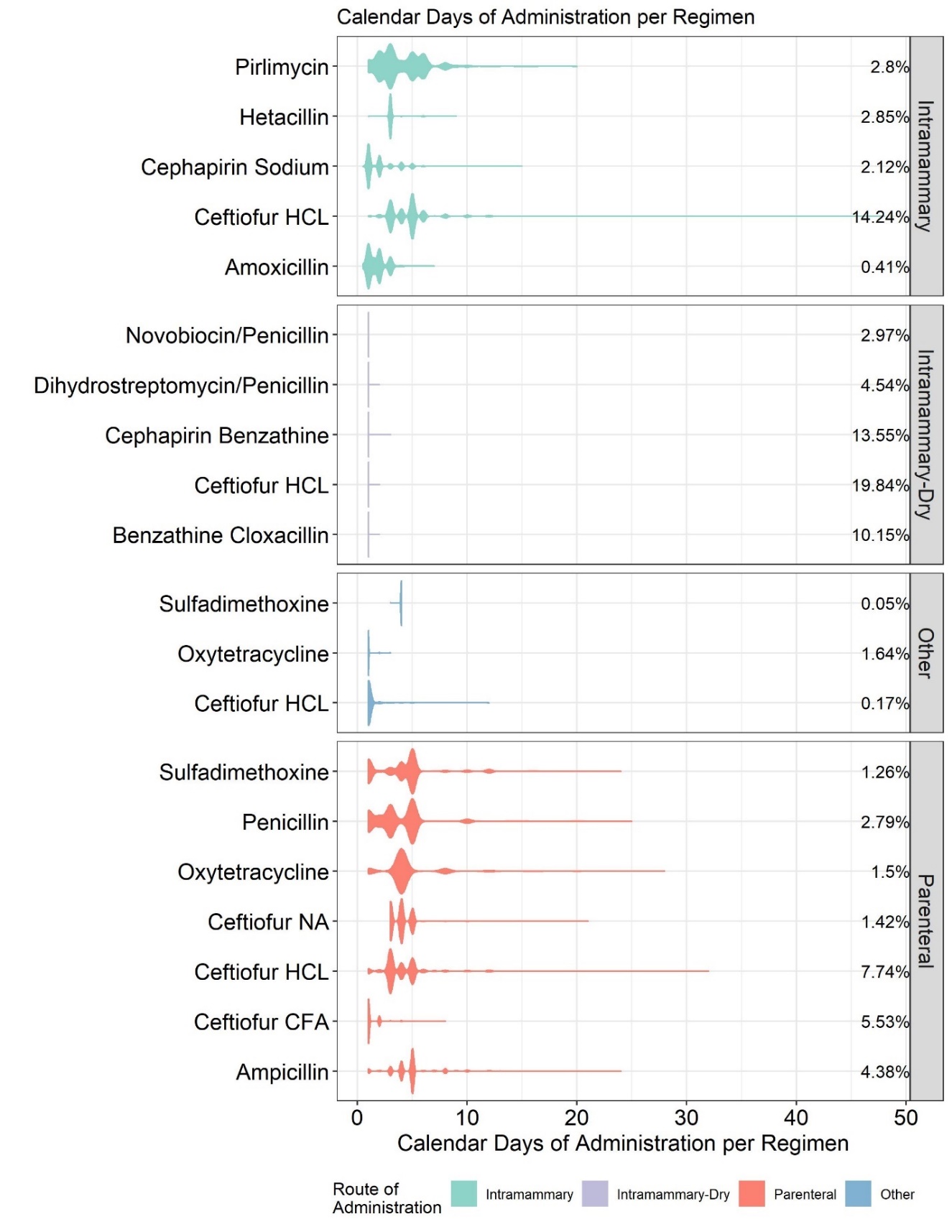


**Figure S1.2**


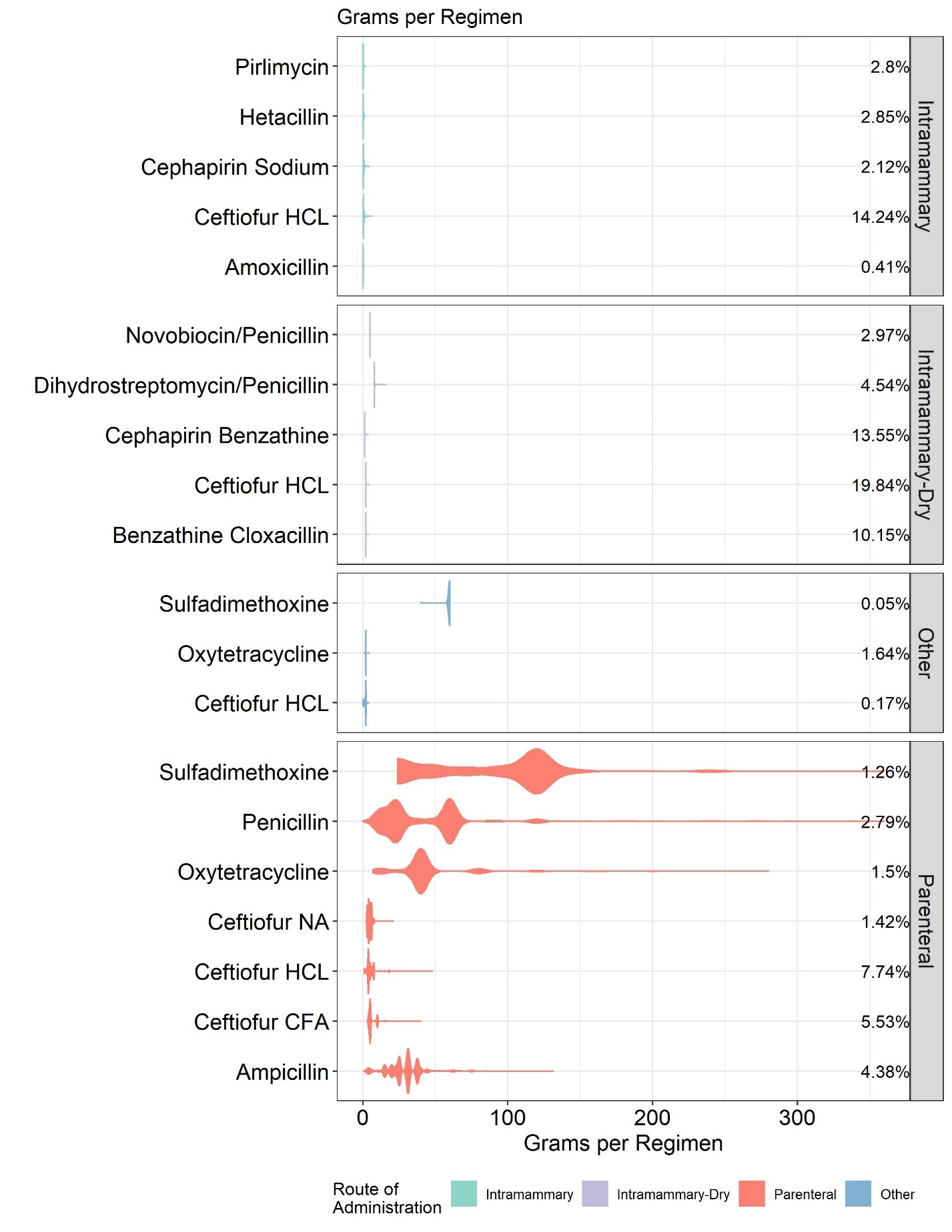


**Figure S1.3**


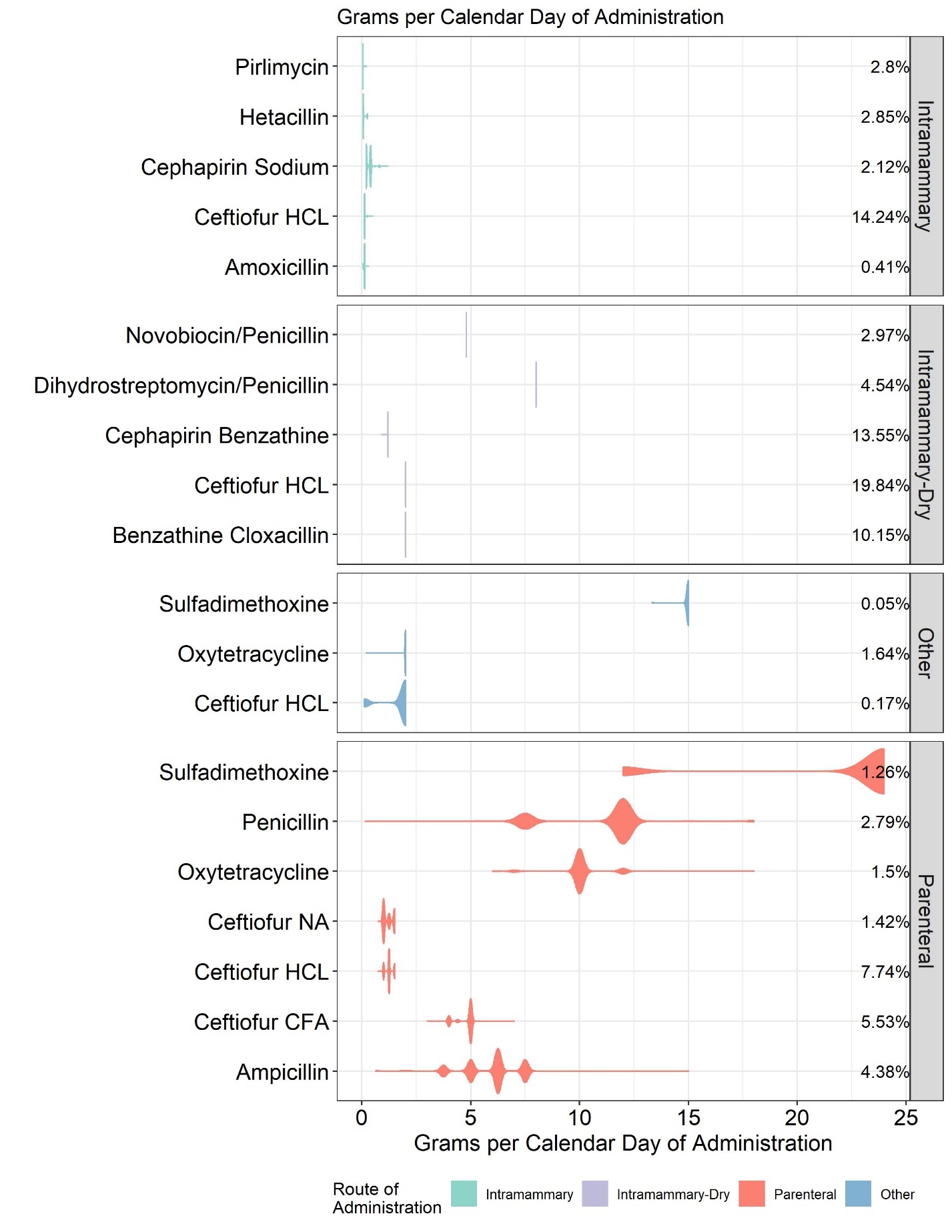


**Figure S1.4**


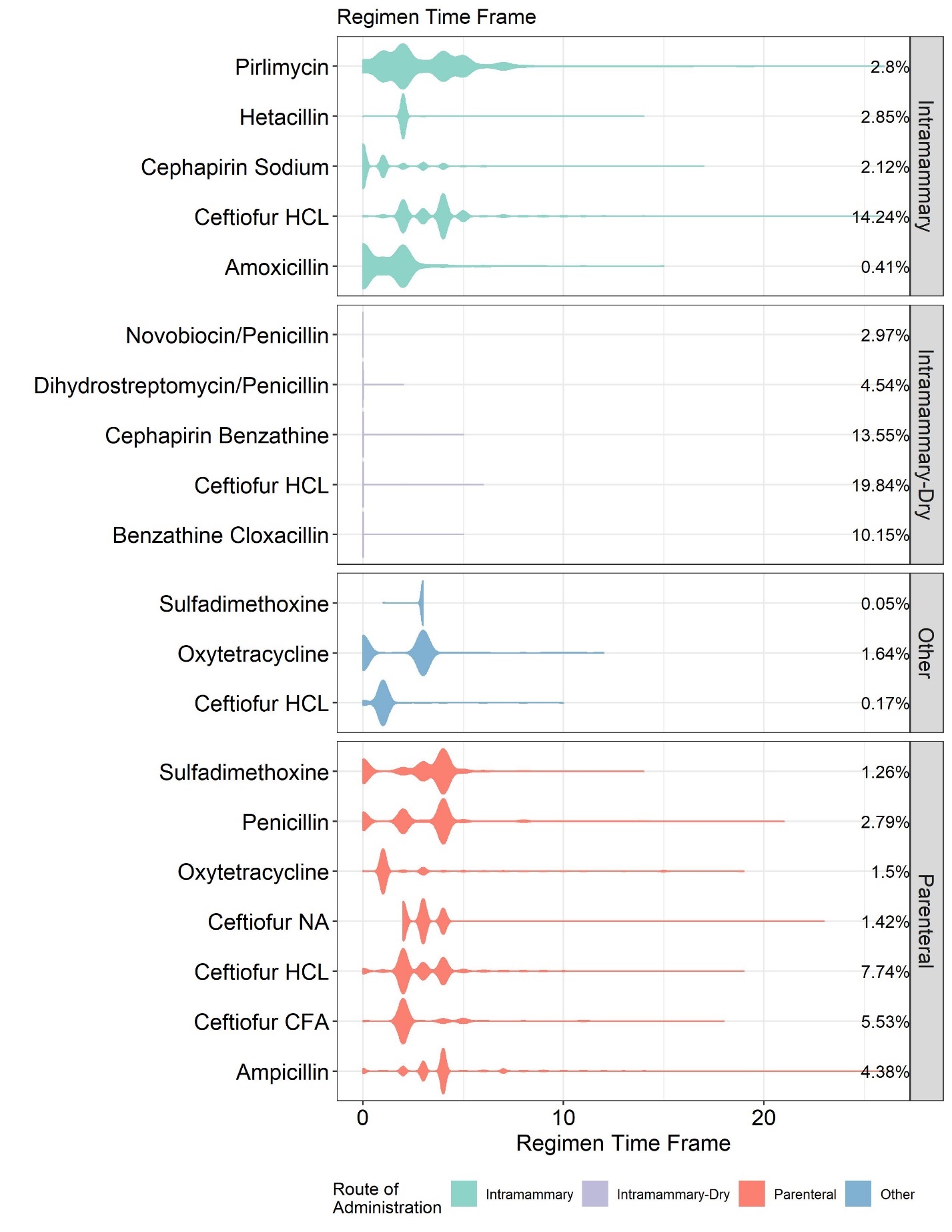


This table is also available as an excel file: “Benchmarking Supplemental Tables_028.xlsx

**Table S1**

## 1.2 Therapeutic event descriptions

Simply stated, a therapeutic event (TE) represents a detected, recorded disease event in an individual animal. Extracting this from a treatment record requires specifications to be made about which rows of original data fit into the same therapeutic event based on the following requirements:

- A TE is cow specific (single animal)
- A TE is NOT drug or medication specific
- A TE may have an unlimited number of drug regimens encompassing an unlimited number of administrations, provided the gap between drug administrations of any product is never >7 days.
- Within a TE, drug regimens may overlap (i.e., administration of 2 different drugs simultaneously)
- A TE is NOT specific to a single disease syndrome, although one is assigned for reporting (see text below), and when multiple disease syndromes are present it is assigned as “complex disease”
- Documentation of “no treat” is included in the TE, and is considered a “no-treat” regimen.
- Disease documentation without explicitly listing a treatment is included in the TE as regimen of an “unknown” product
- Non-antimicrobial drugs (i.e. flunixin, calcium, Keto-Gel) are included in the TE as “Non-Antimicrobial” regimens.
- A TE may have only one outcome, defined on or before day 30 following the final administration in the final regimen of the TE.

Description of therapeutic events was performed using the documented function in R (details in section 3 of this supplemental document.). The result of this function produces rows of data each representing one therapeutic event, while maintaining the information that was in the original data. For example, if more than one disease was documented during the therapeutic event, then all diseases are listed. For this reason, some diseases related to a TE are reported as “complex disease” indicating that multiple disease syndromes were documented within the therapeutic event. This is true for other classification parameters such as drug class, etc.

Numerous aspects of therapeutic events can be described. Distributions of continuous variables such as the total days of therapy can be reported for therapeutic events. The following figures illustrate the distribution of all regimens per TE, antimicrobial regimens per TE, and TE time frame.

**Figure S1.5**


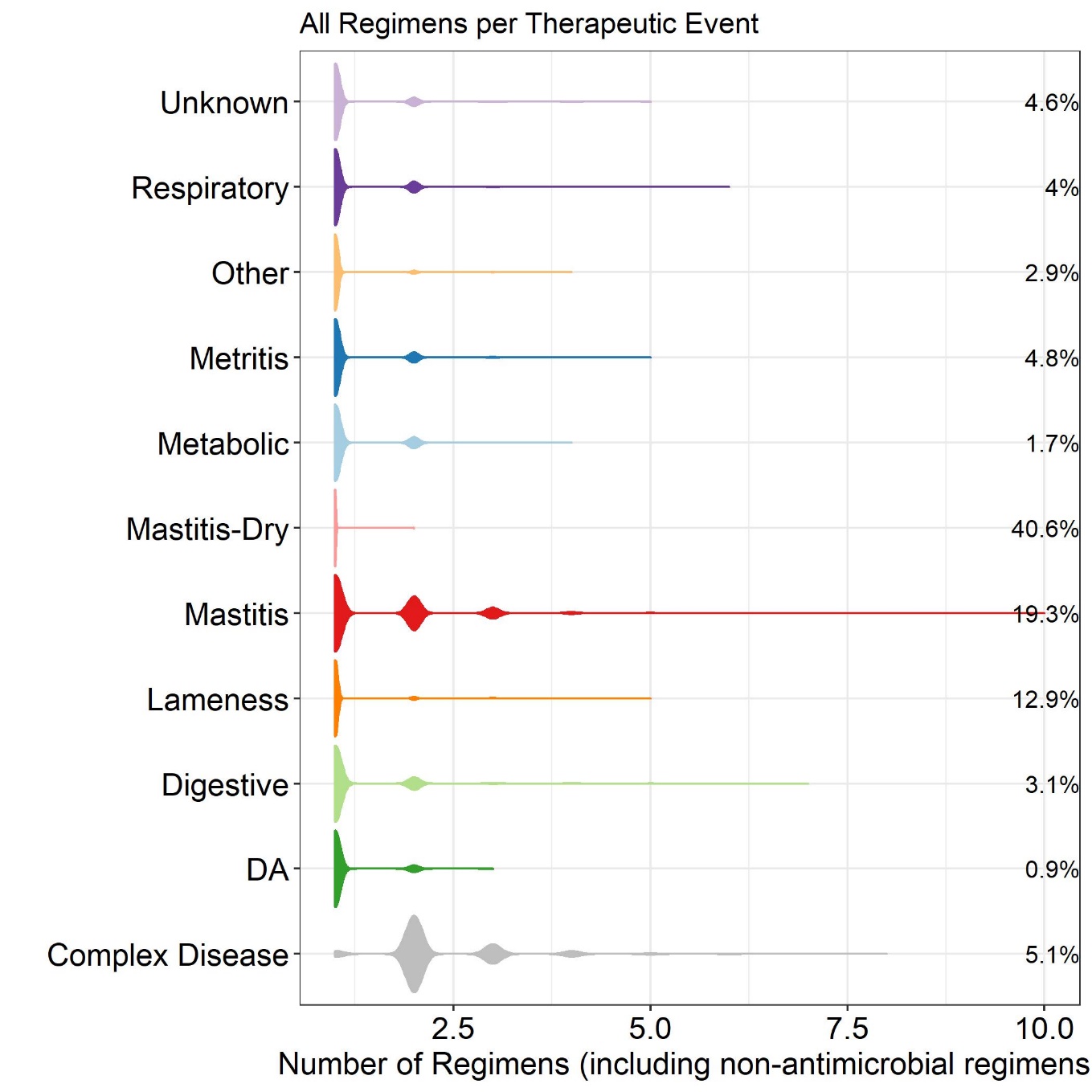


**Figure S1.6**


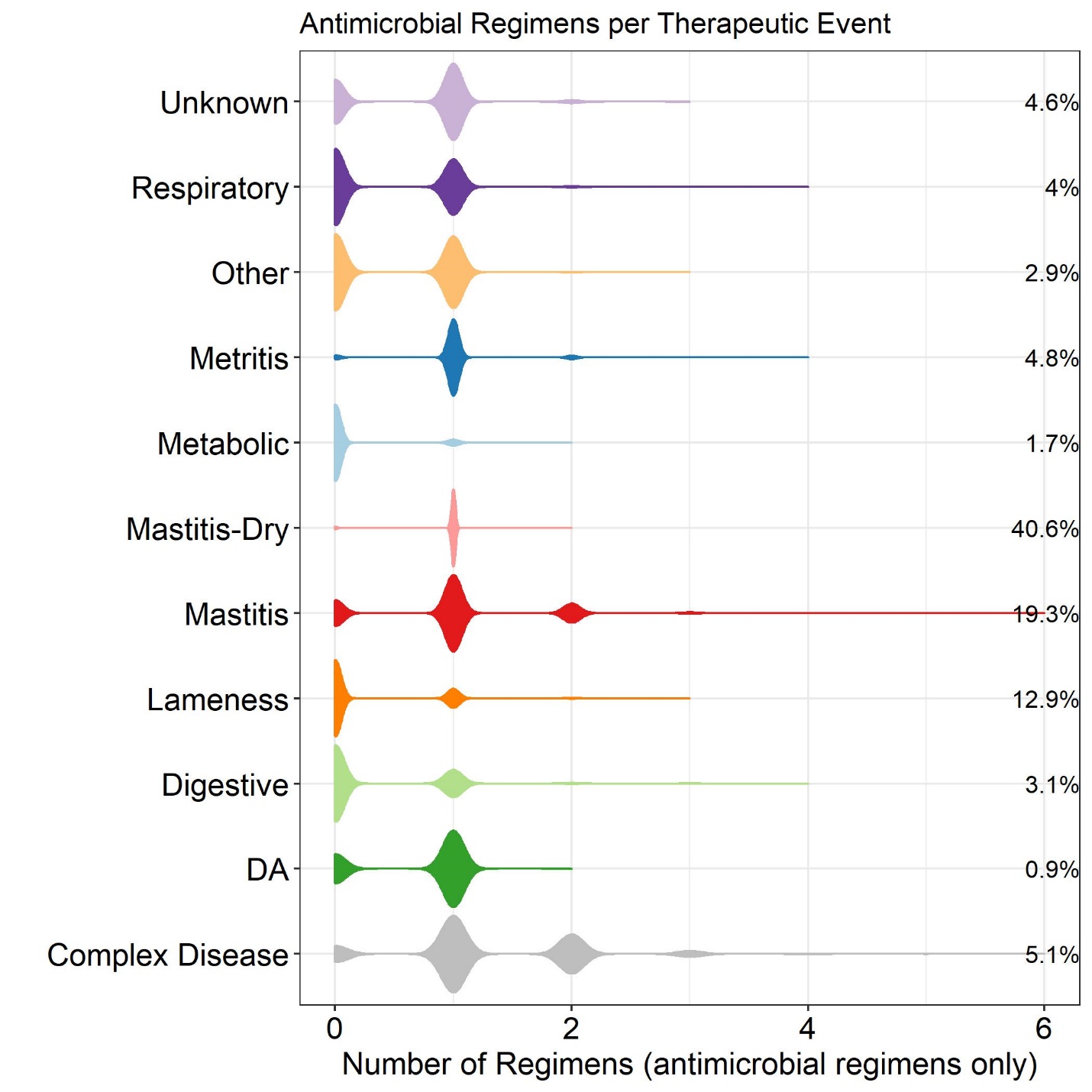


**Figure S1.7**
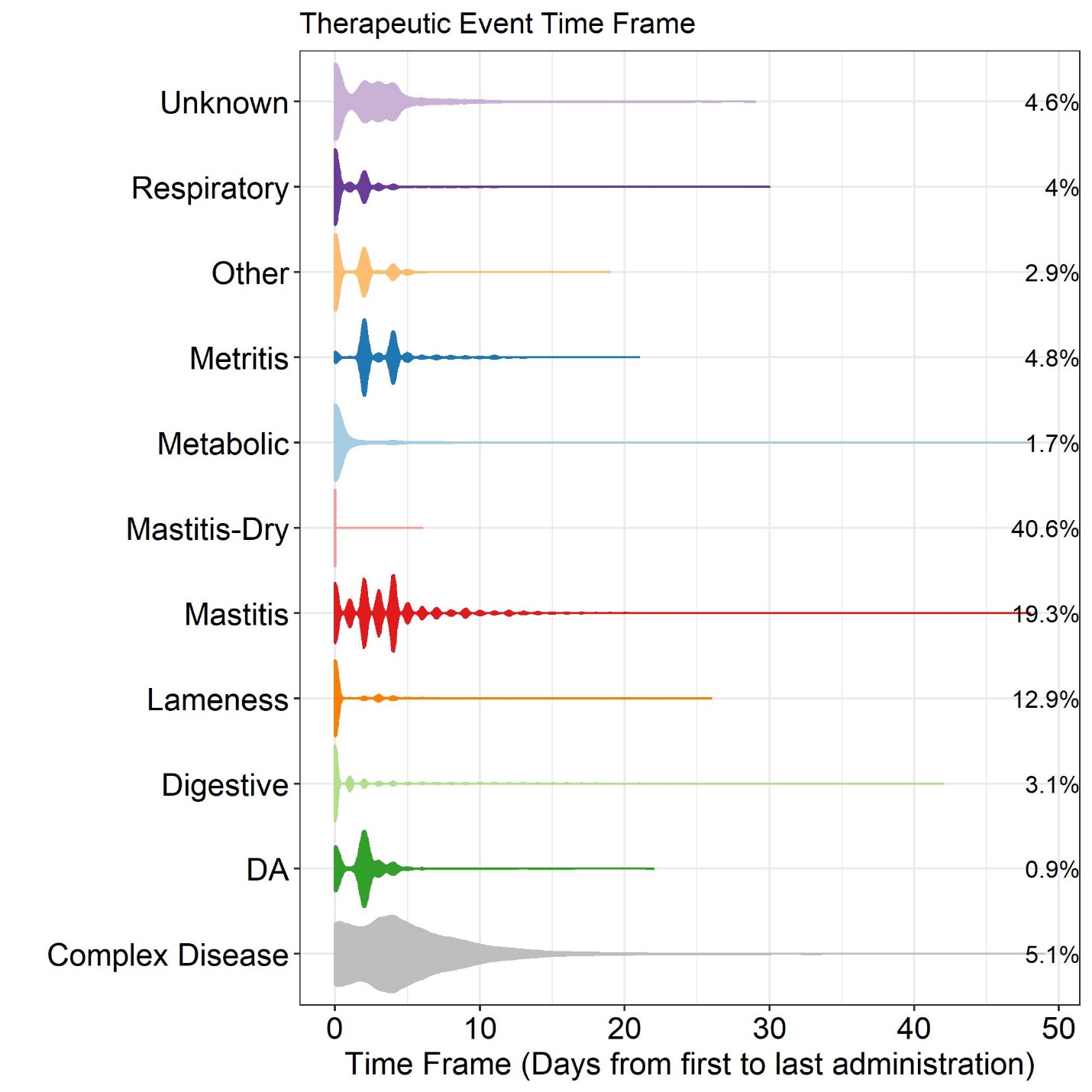


Categorical variables may also be reported by categorizing therapeutic events by any desired parameter. For example, the figure below shows the percentage of all therapeutic events that fell into each disease syndrome category.

**Figure S1.8**


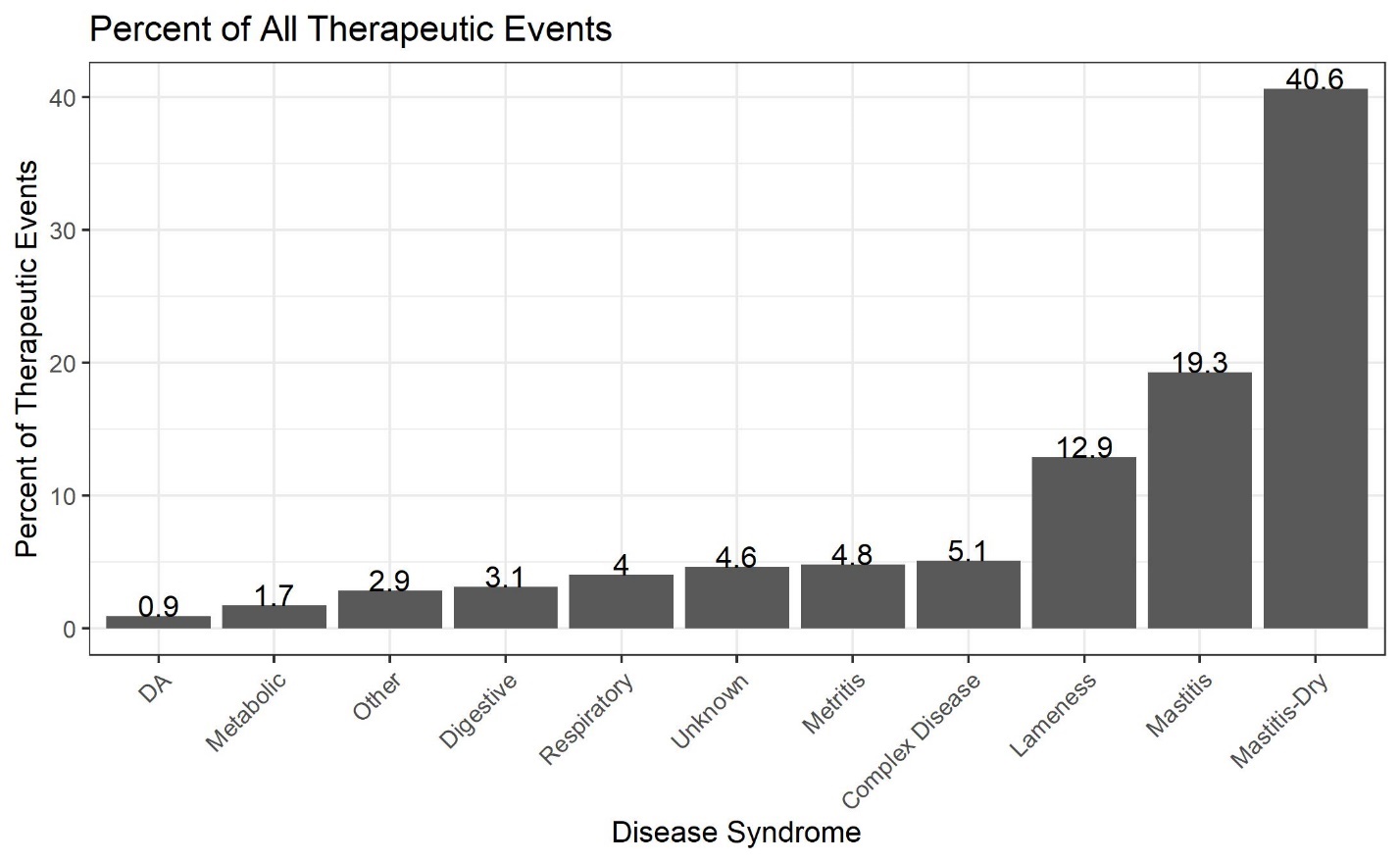


The following figure describes therapeutic events within each disease syndrome by various parameters such as outcomes on day 30, number of antimicrobial regimens included, and what percent of all therapies indicated a “no treat”, ‘non-antimicrobial”, or “unknown” treatment.

**Figure S1.9**


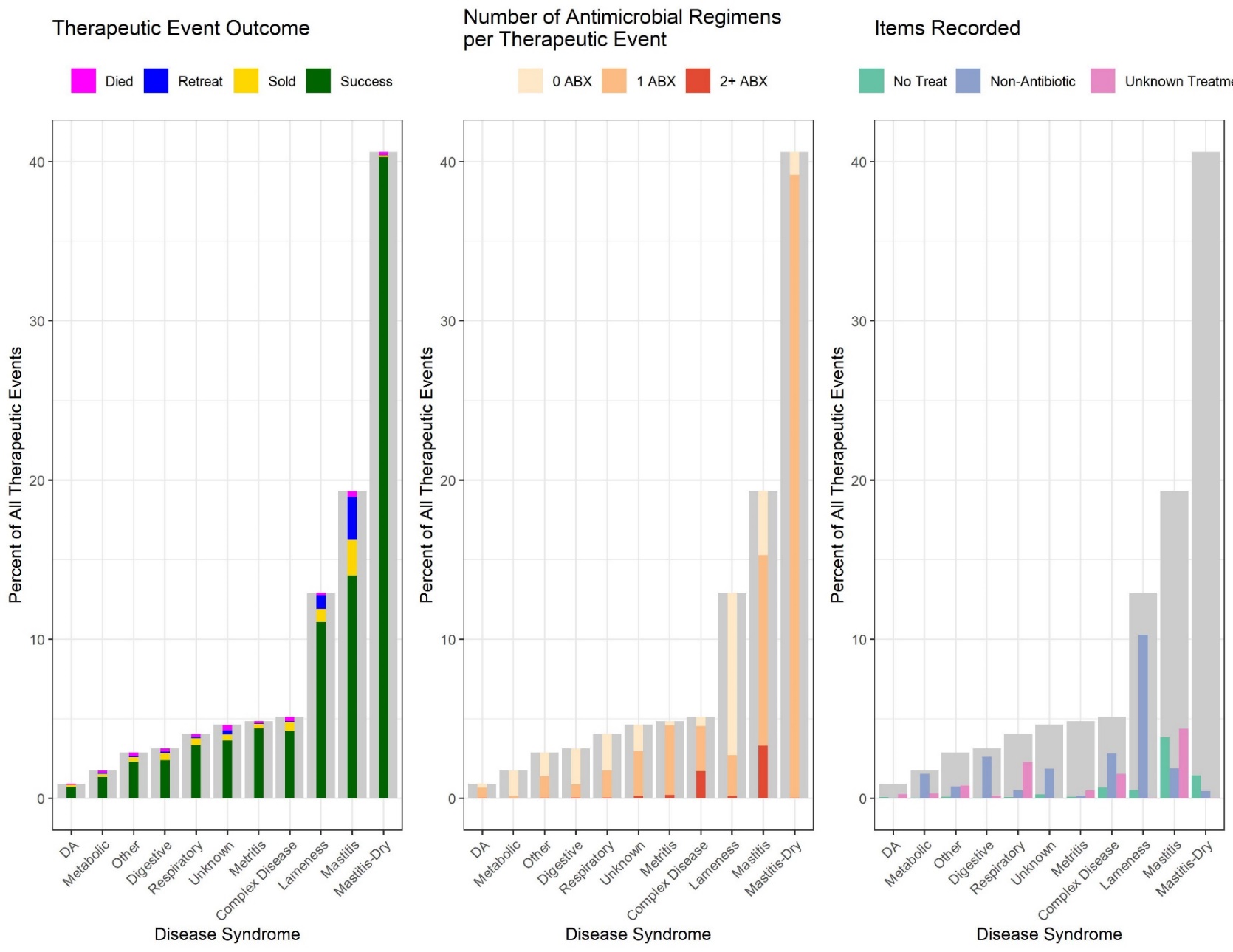


The first column reports the percentage of all TE in each Disease category (the column sum is 100%). The Outcomes and Antimicrobial Regimens sections report the percentage of all therapies within that disease which had the specific outcome or number of antimicrobial regimens in the column heading. This is also true in the Other items recorded section, except that the 3 column headings within this section are not mutually exclusive. Therefore, all values within a particular row and section of the Other items recorded section will not sum to 100% as they will in the Antimicrobial Regimens and Outcome sections.

**Table S1.2**

| **Percent of all Therapies (%)** | **Disease** | **Outcomes** | | | | | **Antimicrobial Regimens** | | | **Other items recorded** | | |
| --- | --- | --- | --- | --- | --- | --- | --- | --- | --- | --- | --- | --- |
|  |  | **Died (%)** | **Sold (%)** | **Retreat (%)** | **Success (%)** | **Unknown (%)** | **Zero (%)** | **One (%)** | **Two or more (%)** | **Unknown Treatment (%)** | **No Treat (%)** | **Non-Antimicrobial Treatment (%)** |
| 5.1 | Complex Disease | 5.47 | 11.03 | 1.11 | 82.4 | 0.017 | 11.57 | 55.05 | 33.38 | 29.7 | 13.0 | 55.1 |
| 0.9 | DA | 8.02 | 14.07 | 0.20 | 77.7 | 0.049 | 27.46 | 71.85 | 0.69 | 27.8 | 7.3 | 1.4 |
| 3.1 | Digestive | 6.14 | 13.99 | 3.31 | 76.5 | 0.014 | 72.54 | 26.05 | 1.41 | 4.8 | 0.3 | 83.1 |
| 12.9 | Lameness | 1.12 | 6.46 | 6.64 | 85.8 | 0.017 | 79.17 | 19.86 | 0.96 | 0.2 | 3.9 | 79.7 |
| 19.3 | Mastitis | 1.84 | 11.64 | 14.01 | 72.4 | 0.069 | 20.89 | 62.00 | 17.11 | 22.6 | 20.0 | 9.7 |
| 40.6 | Mastitis-Dry | 0.47 | 0.32 | 0.02 | 99.2 | 0.001 | 3.50 | 96.48 | 0.02 | 0.0 | 3.5 | 1.1 |
| 1.7 | Metabolic | 7.49 | 9.88 | 5.26 | 77.3 | 0.077 | 92.38 | 7.44 | 0.18 | 17.7 | 0.5 | 87.7 |
| 4.8 | Metritis | 2.41 | 5.43 | 1.01 | 91.1 | 0.046 | 4.99 | 90.56 | 4.45 | 10.1 | 1.7 | 3.1 |
| 2.9 | Other | 7.65 | 10.48 | 1.79 | 80.1 | 0.016 | 51.46 | 48.23 | 0.31 | 27.5 | 3.3 | 24.8 |
| 4.0 | Respiratory | 3.98 | 11.35 | 2.53 | 82.1 | 0.044 | 57.39 | 41.52 | 1.09 | 56.3 | 1.6 | 12.0 |
| 4.6 | Unknown | 7.09 | 8.27 | 5.65 | 78.9 | 0.096 | 35.73 | 61.59 | 2.68 | 0.0 | 5.1 | 40.1 |

## 1.3 Complex Disease Details

The combinations of diseases included in therapeutic events classified as complex disease were variable. Shown in the graph below is the percent of therapeutic events classified as complex disease in which the named disease on the x axis was detected in the therapeutic event. These do not sum to 100% because any number of diseases can be included in each complex disease therapeutic event. In the graph below, detection of one disease doesn’t exclude others.

**Figure S1.10**


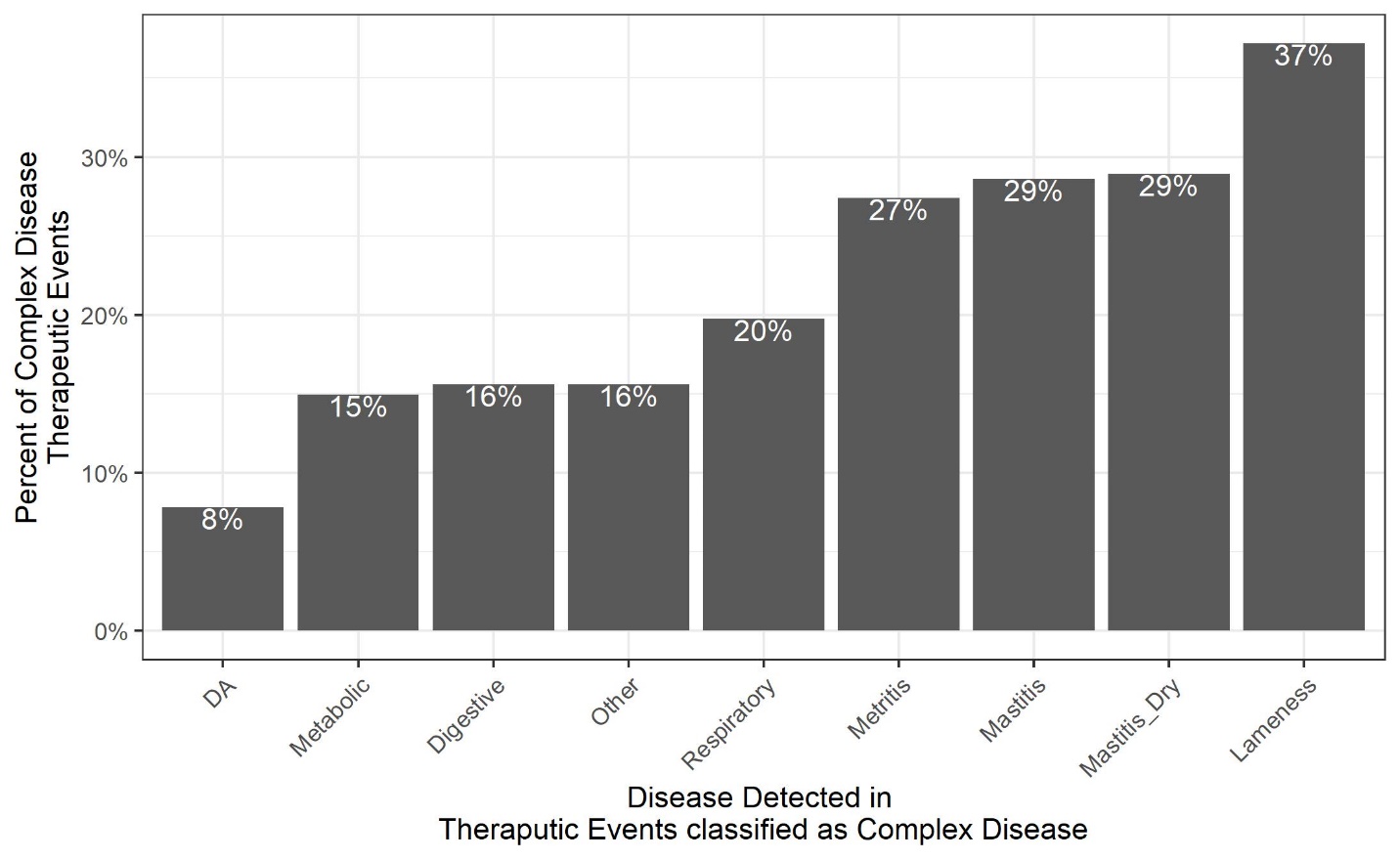


The graph below shows specifically which diseases were included in the most common disease combinations identified as “Complex Disease”. As demonstrated by the relatively large proportion in the “Other combination” category, the combinations were highly variable. Each specific disease combination within this “Other Combination” category represented less than 2% of complex disease therapeutic events.

**Figure S1.11**


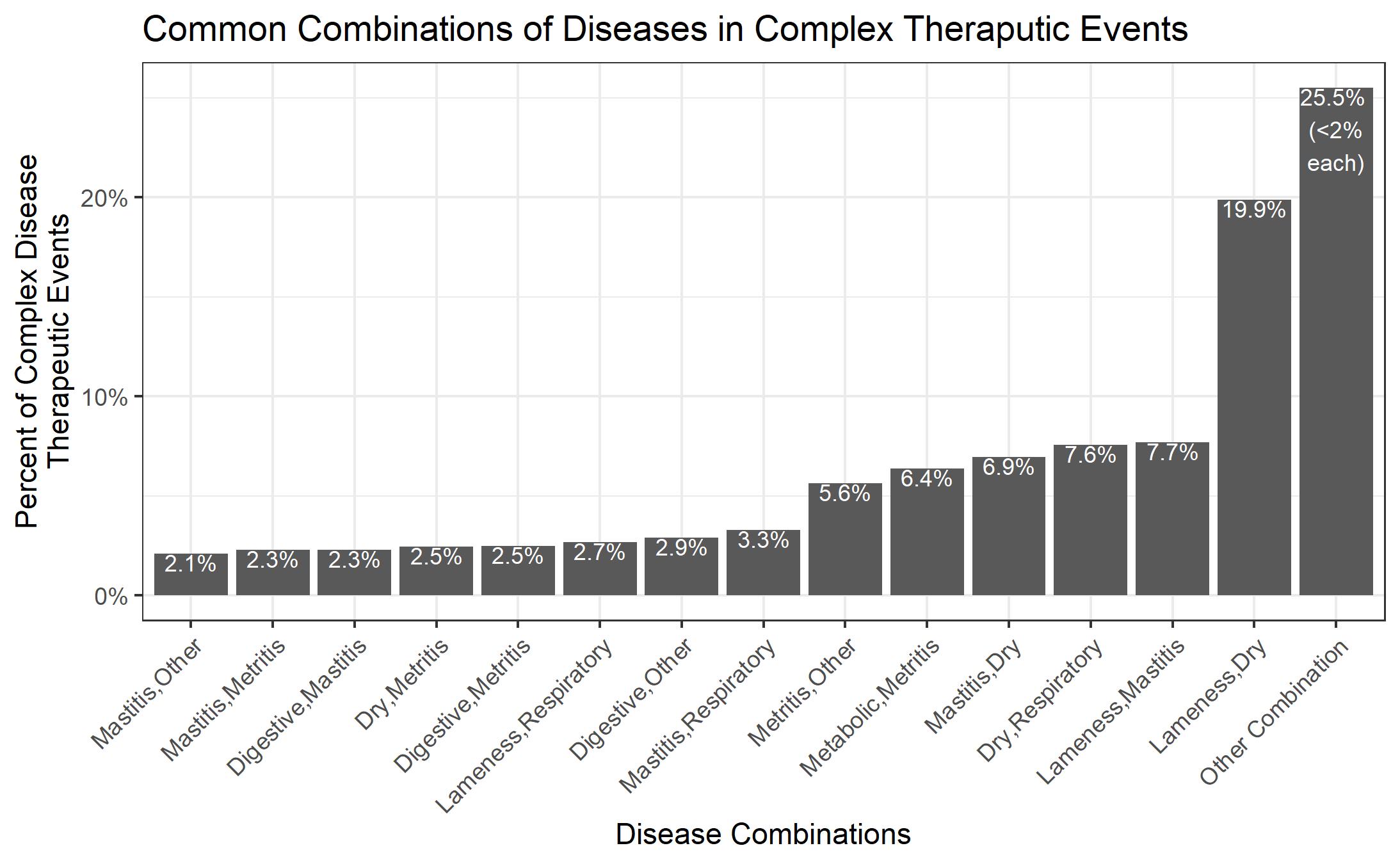


# 2. Relative contributions by disease as a scatter plot.

The relative contributions of each disease can be summarized as a scatter plot, as illustrated below, with therapies for dry cow contributing the highest percentage of antimicrobial regimens, followed by clinical mastitis treatment, and metritis. Respiratory, Lameness, and Unknown disease syndromes all contribute similarly to antimicrobial regimens, however their variability at the farm level is noticeably more than for the first 3 diseases. This variability increases even more for the diseases treated less frequently with antimicrobials such as complex disease (more than 2 disease syndromes associated with a therapeutic event), other (i.e., hardware disease, injury, etc.), and displaced abomasum (“DA”). Not shown in this figure is use for metabolic and digestive disease syndromes. While the frequency of these diseases was often relatively high, the percentage of therapies including an antimicrobial was exceptionally low for these disease syndromes. When antimicrobials were included, this often bumped these therapy events into the complex disease category. For example, if a cow was treated for metabolic disease and subsequently developed metritis which required antimicrobial therapy, the disease syndrome associated with that therapy was complex disease.

**Figure S2.1**


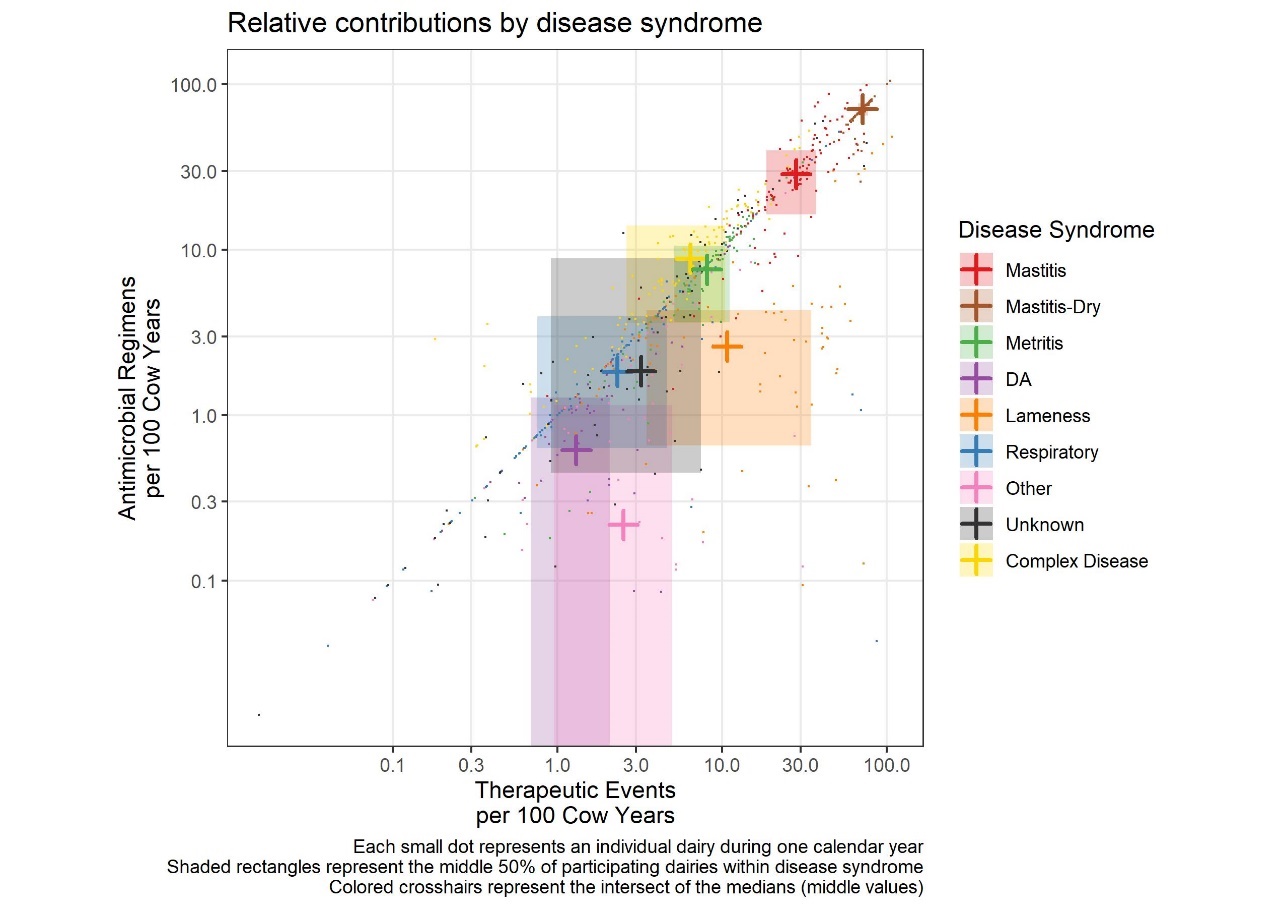


# 3. R code to identify therapeutic events and treatment regimens

For a detailed explanation and the R code used to create therapeutic event grouping variables go to:

[https://](https://drive.google.com/drive/folders/1vpuj2TqqZMAq3RQ5D6RI5doyKeI3CIP1?usp=sharing)drive.google.com/drive/folders/1vpuj2TqqZMAq3RQ5D6RI5doyKeI3CIP1?usp=sharing The link contains an HTML file (Lag-Function_Documentation---Dairy.html) that shows both the code and the output for a sample data set.

If you prefer to run the code yourself, download the markdown file with the same name and the sample data set, test_data_frame_for_lag.csv.

# 4. Training figures for graphics

The goal of reporting was to provide a format that would allow a user to evaluate multiple metrics very efficiently. Because it is recognized that individuals often have strong preferences for the format by which data are presented, multiple types of graphics are utilized for reporting: scatter plots, tabular output. Ideally all of these graphics would be presented in a dynamic format such as a shiny app or other interactive tool which would allow the user to turn graphical layers on and off, filter by specific diseases, and choose the format that is most relevant for them.

Because the scatter plots can be complex with their many layers, the following sequence of graphs builds them in a stepwise fashion. This allows those unfamiliar with the plots to rapidly understand what is represented by each layer.

## 4.1 Scatter plots – Figure Description

**Figure S4.1**


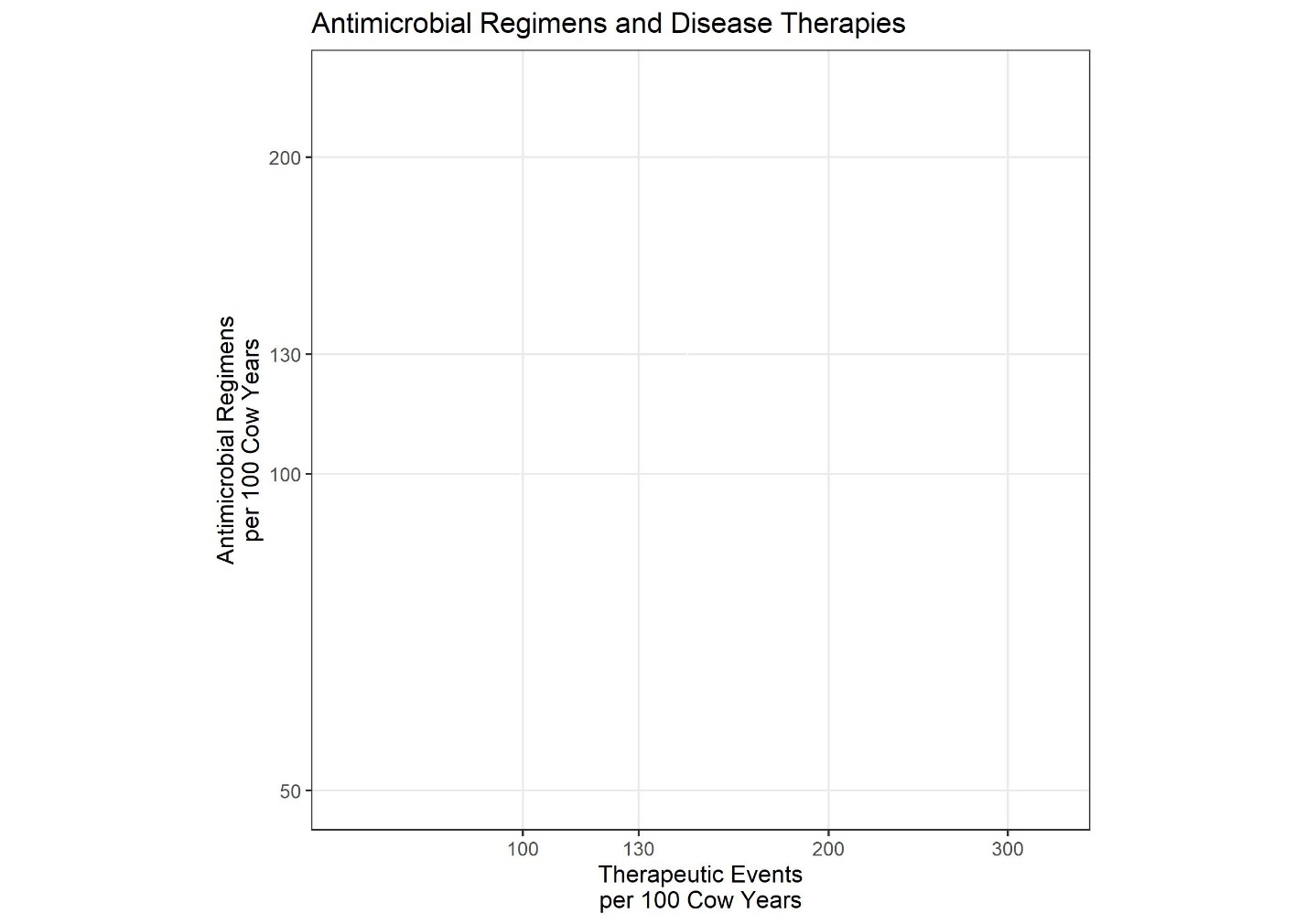


Graph is intentionally blank

X axis – Represents Disease Incidence per 100 Cow Years

Y axis – Antimicrobial Use per 100 Cow Years

**Figure S4.2**


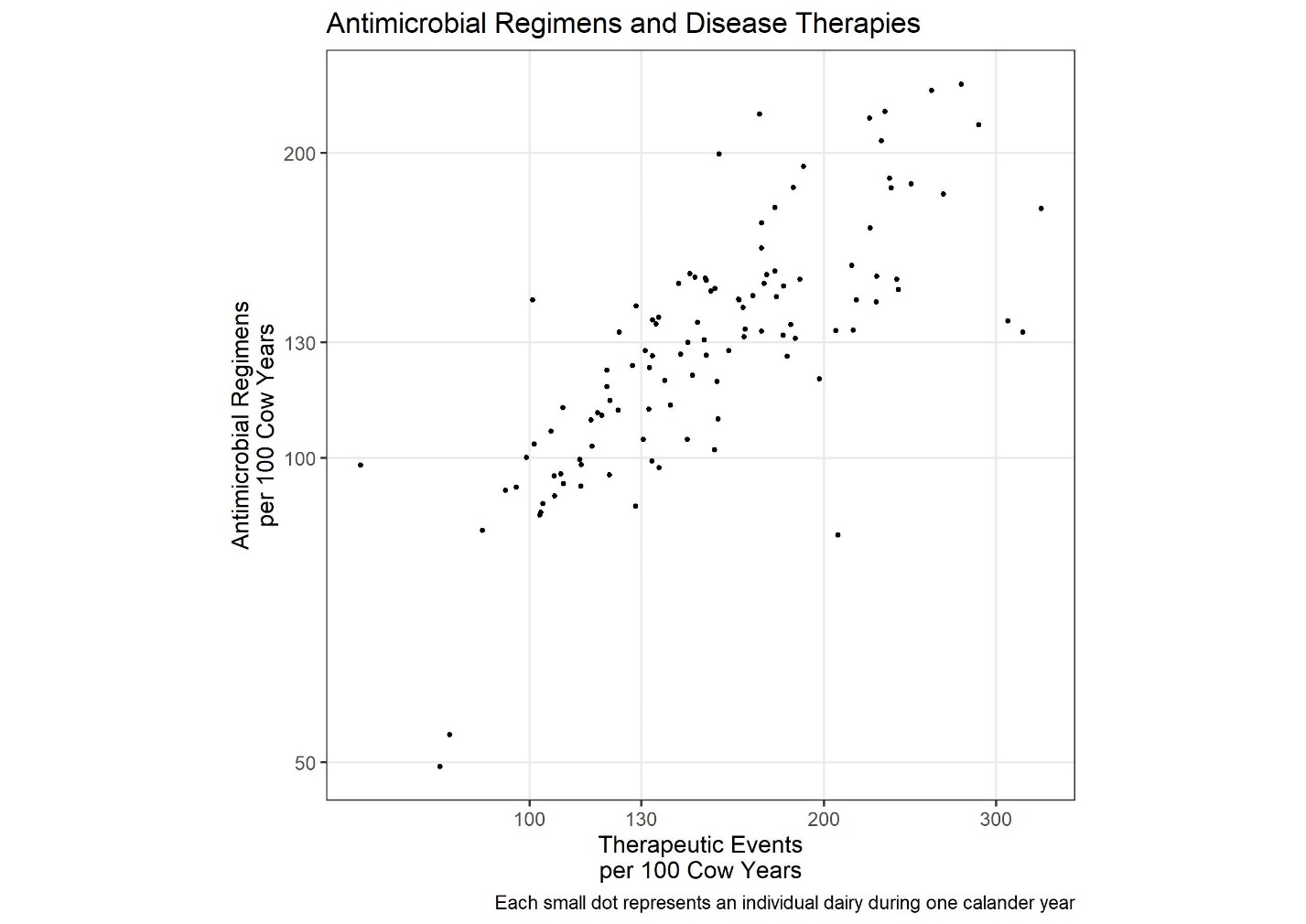


Scatter plot of all dairies – each dot is one calendar year from 2016-2019 for each dairy in the study

**Figure S4.3**


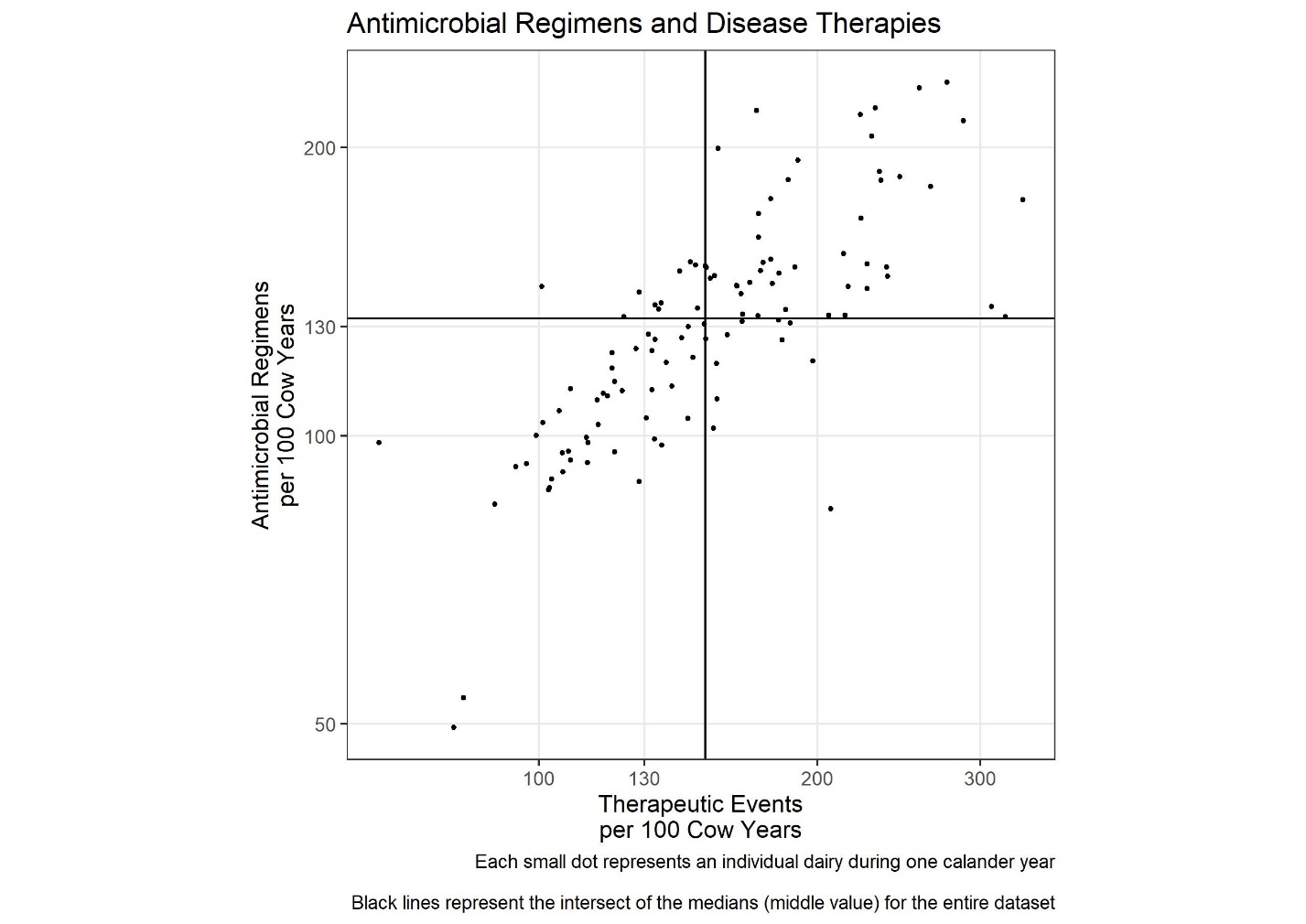


Black crosshairs represent the median value (middle dairy) on each axis for all years and all dairies combined

**Figure S4.4**


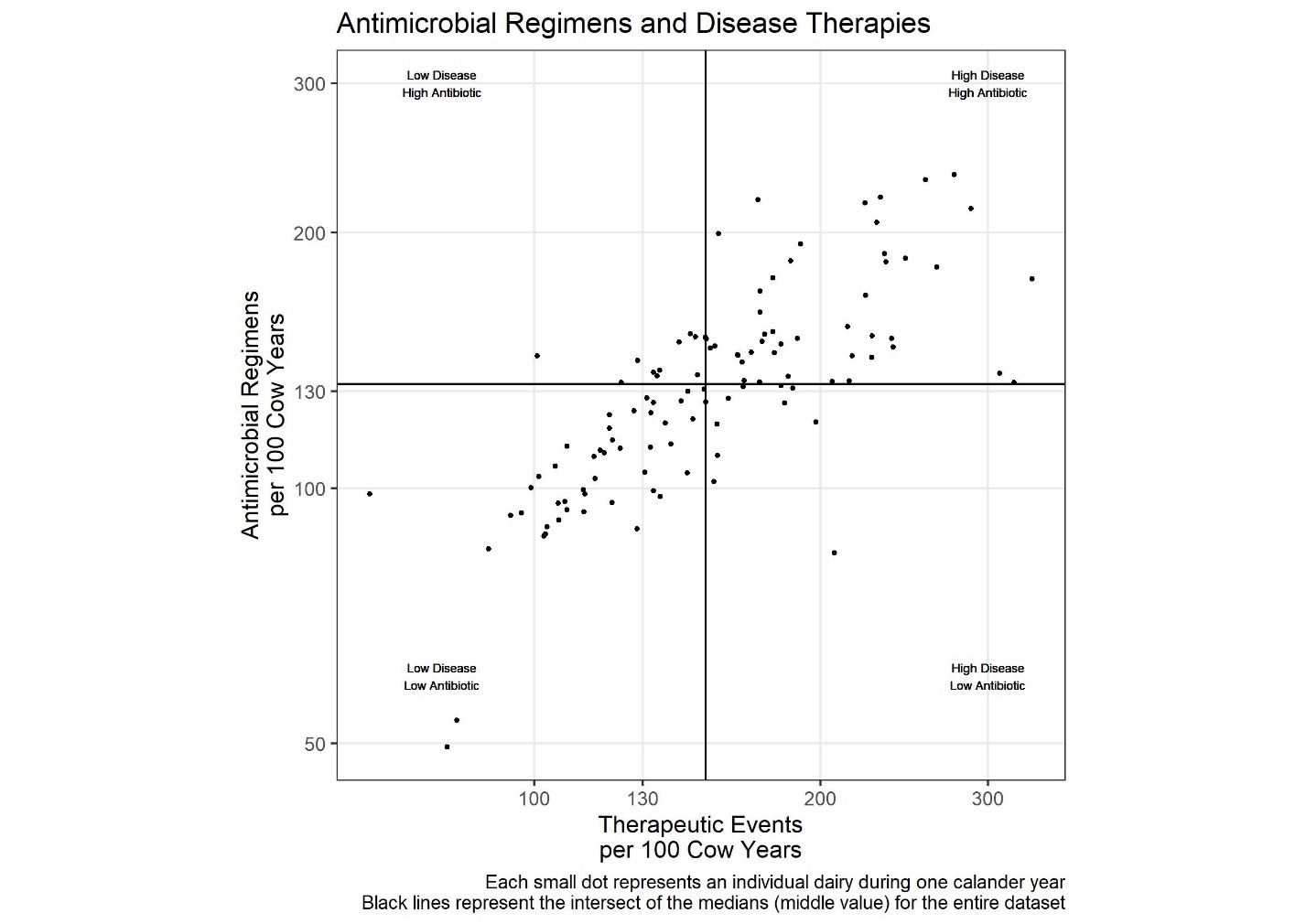


The cross hairs create 4 quadrants each representing a different combination of values on each axis.

- Upper Left – Low Disease, High Antibiotic Use
- Upper Right – High Disease, High Antibiotic Use
- Lower Left – Low Disease, Low Antibiotic Use
- Lower Right – High Disease, Low Antibiotic Use

**Figure S4.5**


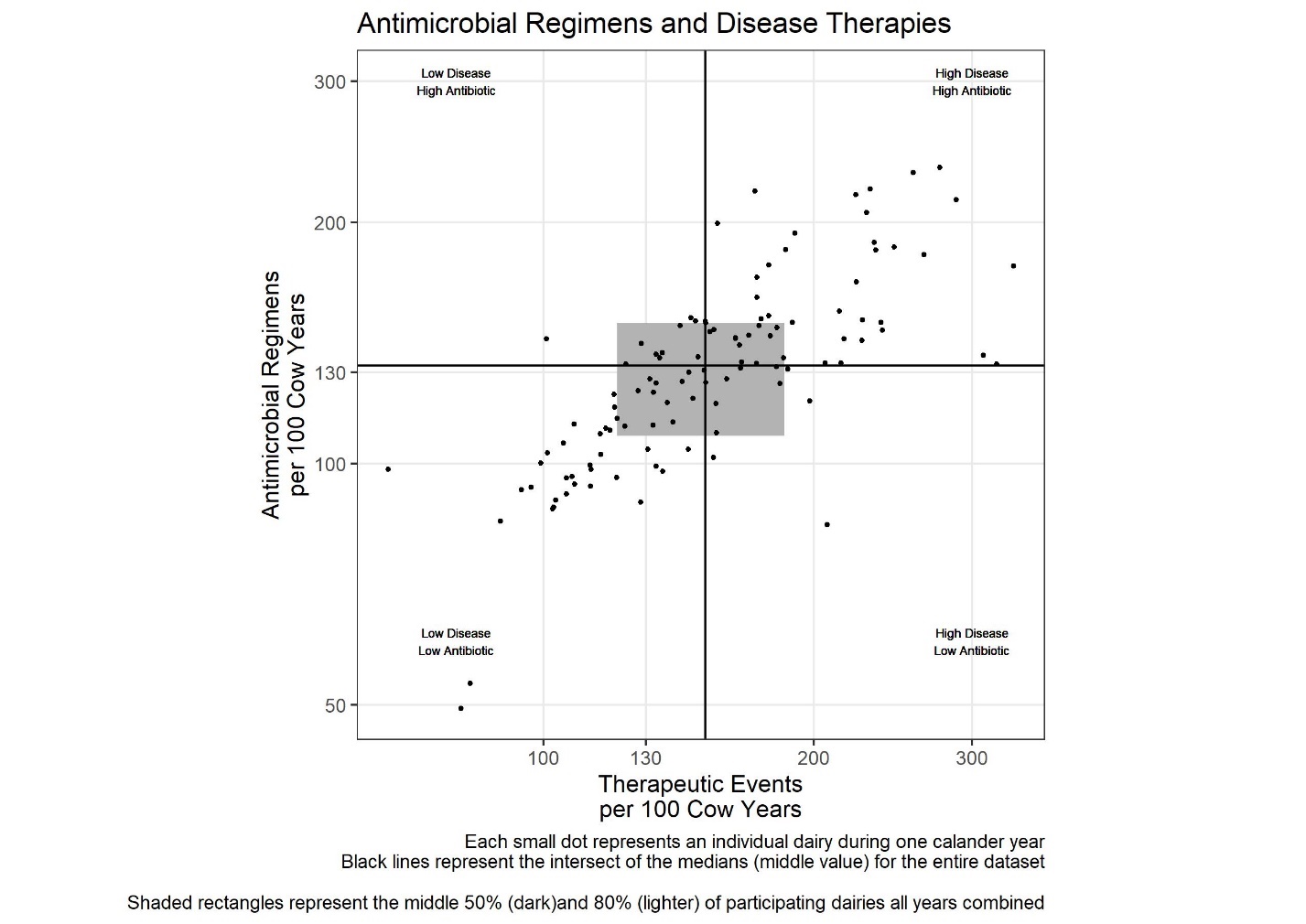


The dark grey shaded region represents the area containing the middle 50% (2^nd^ and 3^rd^ quantiles) on each axis.

**Figure S4.6**


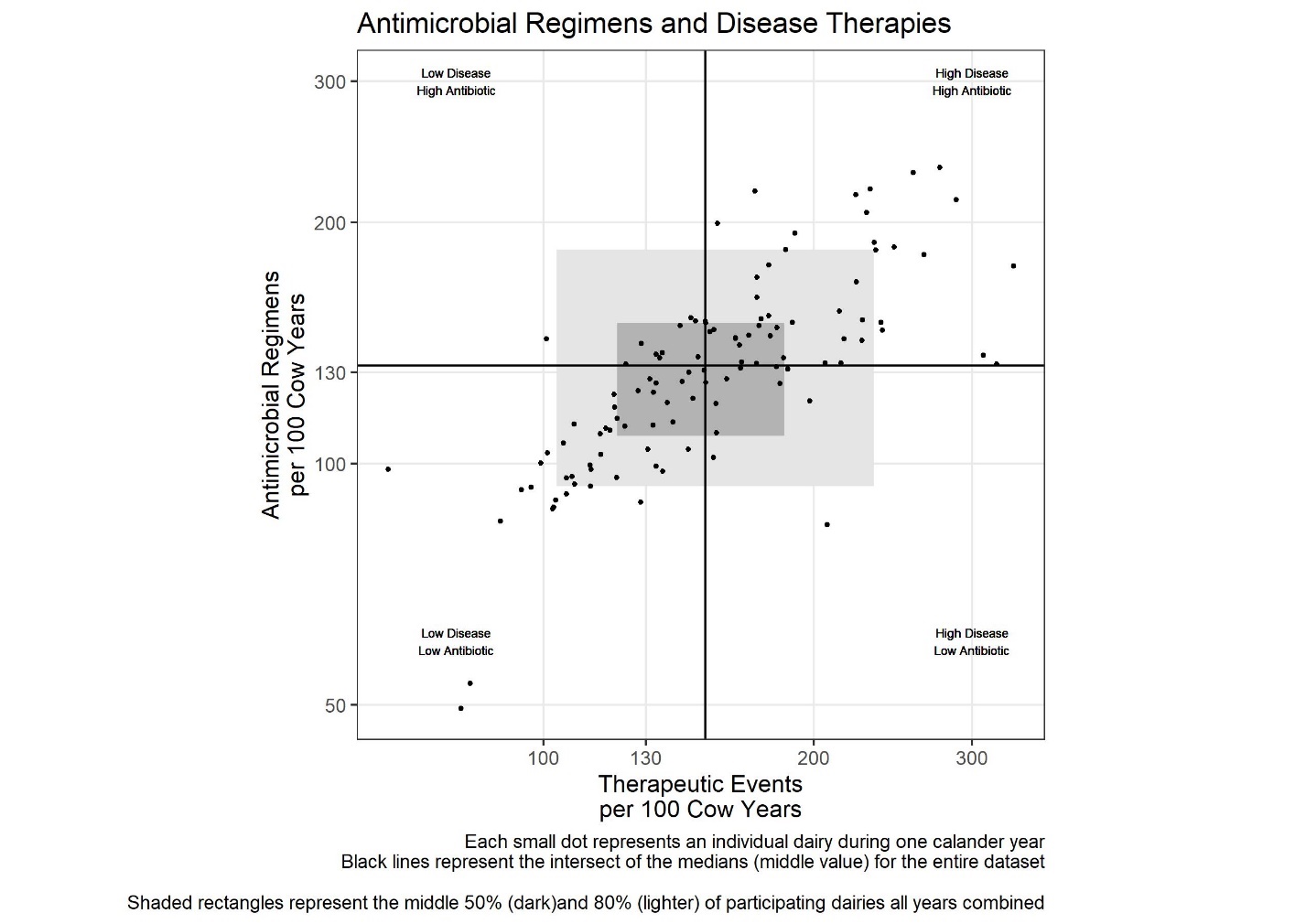


Light grey shaded region represents the area between outside the middle 50% and within the middle 80% on each axis.

**Figure S4.7**


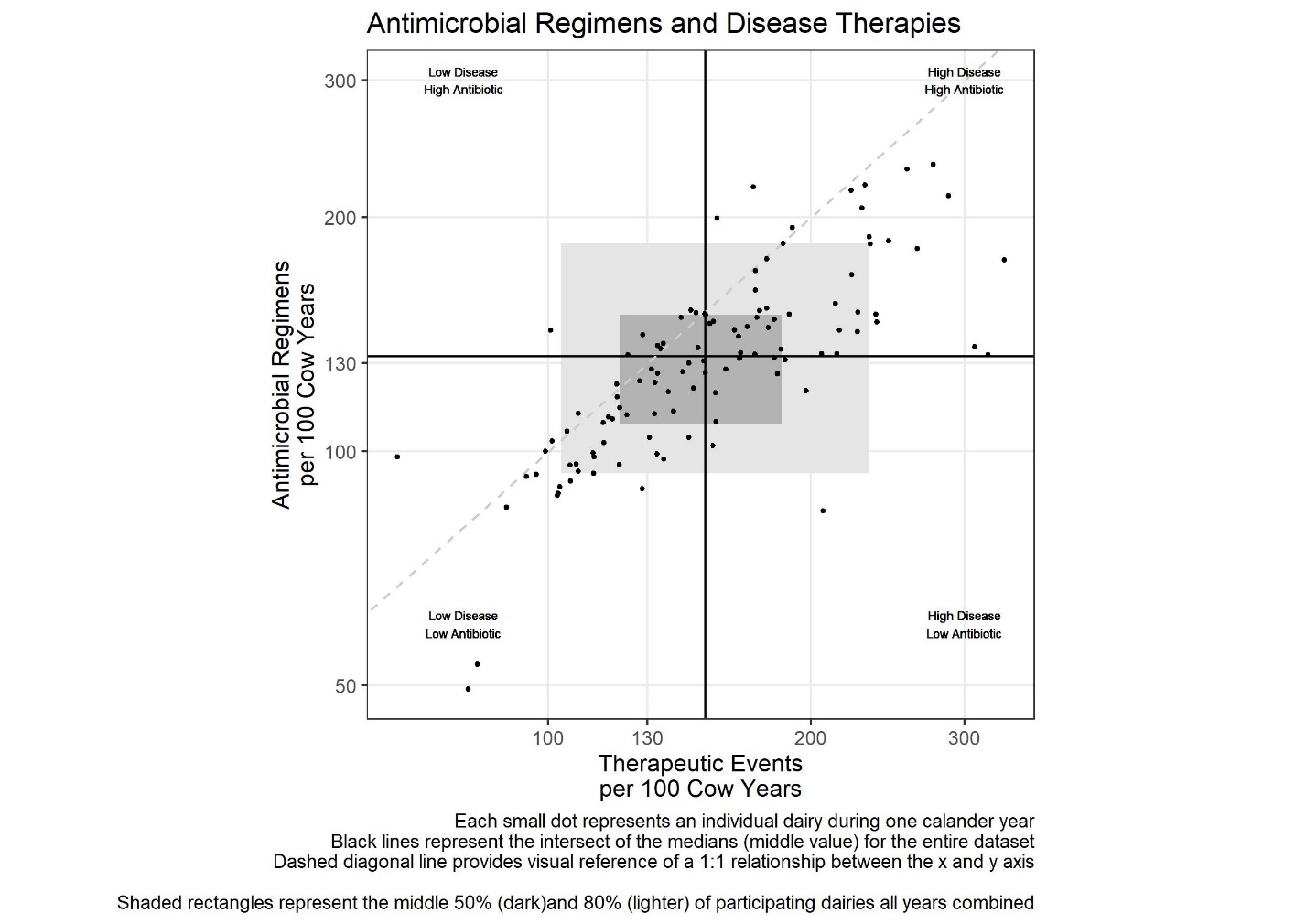


The dashed line is for visual reference only, and represents a 1:1 ratio between the x and y axis.

## 4.2 Scatter plots – Individual Farm Reports

For individual farm benchmark reports (below) colored points represent an individual dairy, and colors indicate the category of % Success for this farm, when compared to other farms in the study.

Very High >80^th^ percentile

High >60-80^th^ percentile

Average >40-60^th^ percentile

Low >20-40^th^ percentile

Very Low - ≤20^th^ percentile

**Figure S4.8**


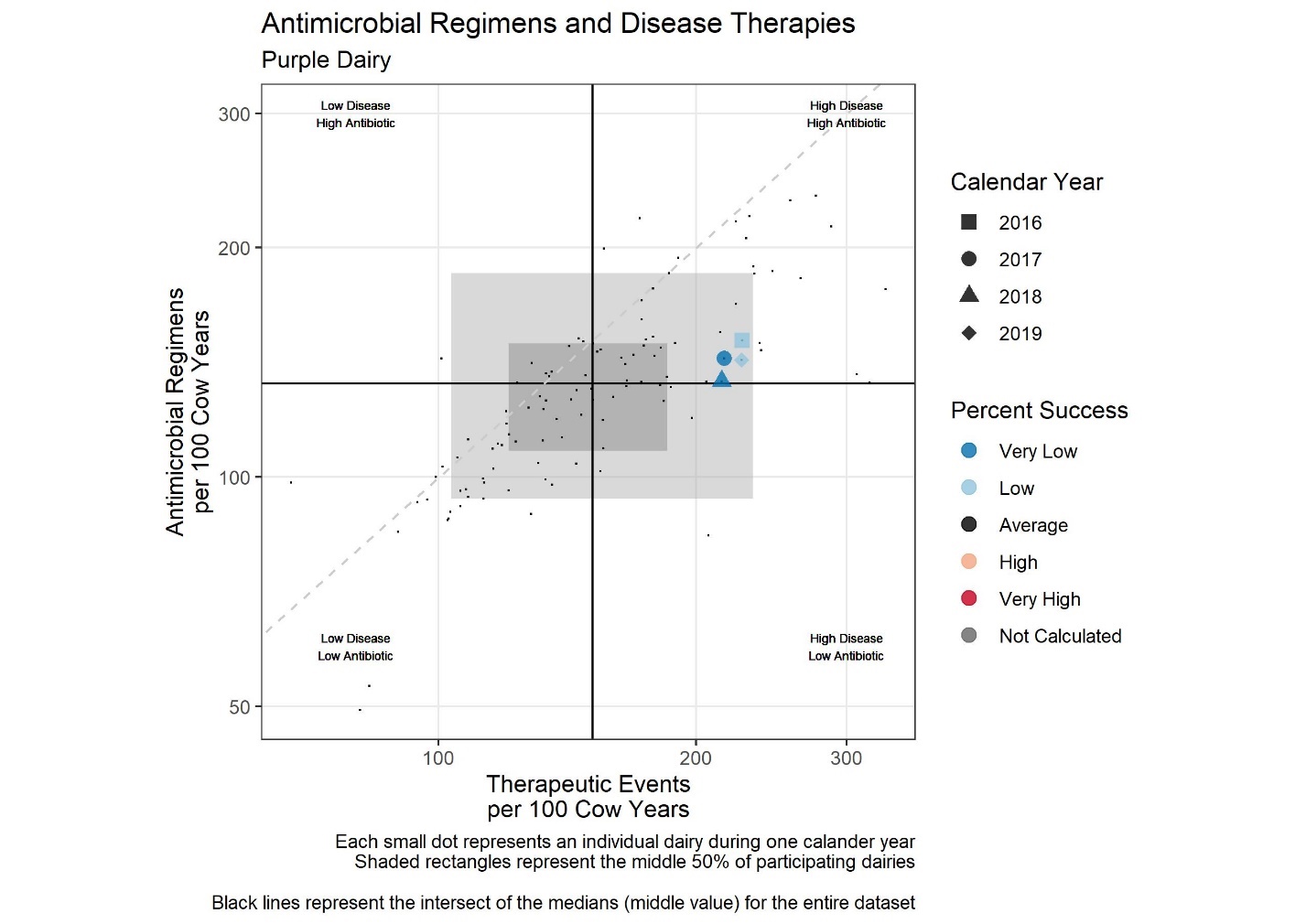


**Figure S4.9**


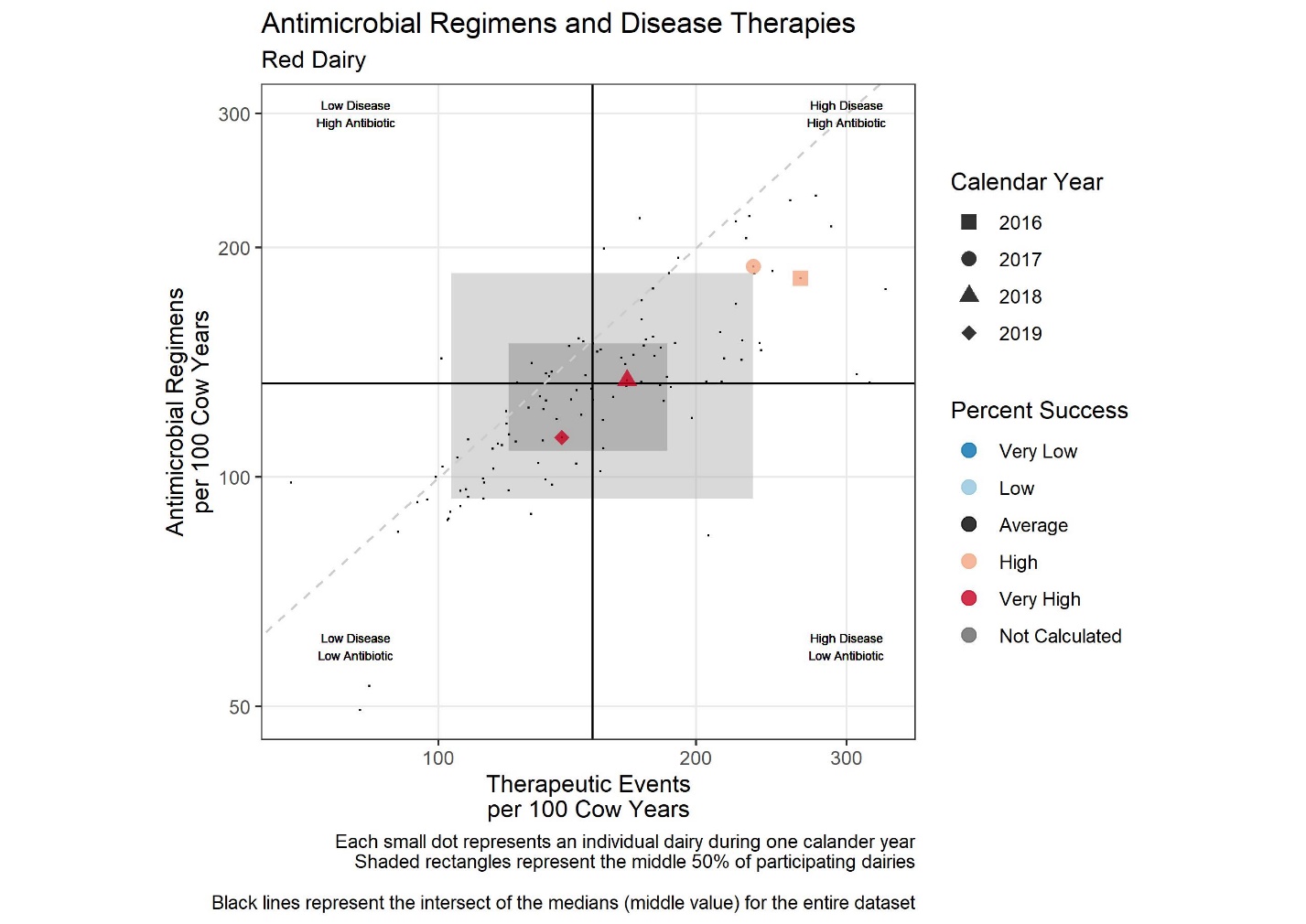


For individual farms each disease can be reported separately which facilitates interpretation of outcomes.

**Figure S4.10**


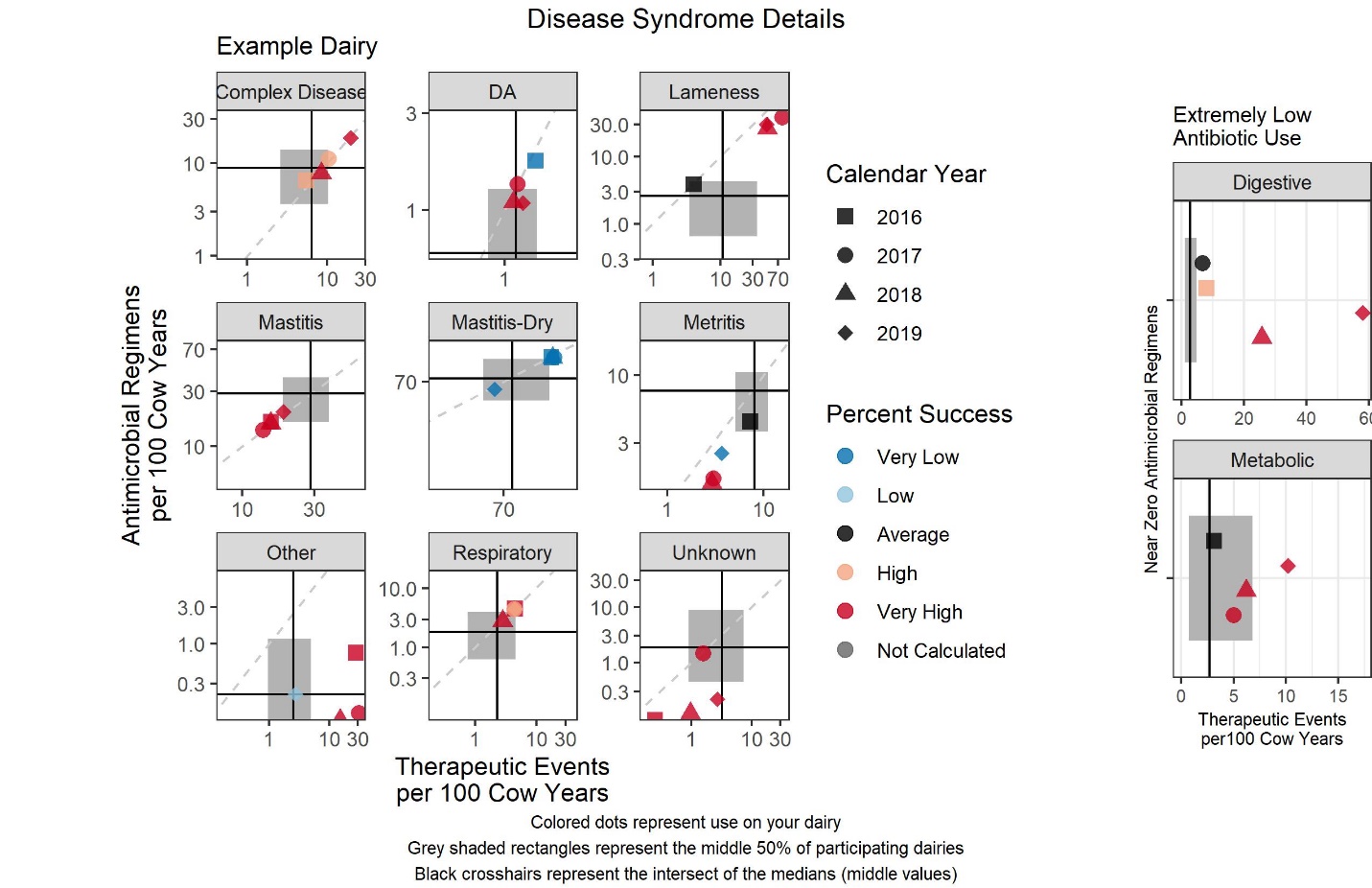


Antimicrobial use is extremely low for Digestive and Metabolic disease making the plot difficult to read, so only Therapeutic Events (Disease) are reported for these 2 disease syndromes. A farm’s antimicrobial use for these 2 disease syndromes can be evaluated by examining their RT-ratios in the tabular format (see supplemental section 4.4)

## 4.3 Scatter plots – Multiple farms

When multiple farms are reported (i.e. a report to a veterinary clinic), farms can be compared within the same plot by mapping color to farm name rather than to % Success.

**Figure S4.11 (same as main paper Figure 1)**

**
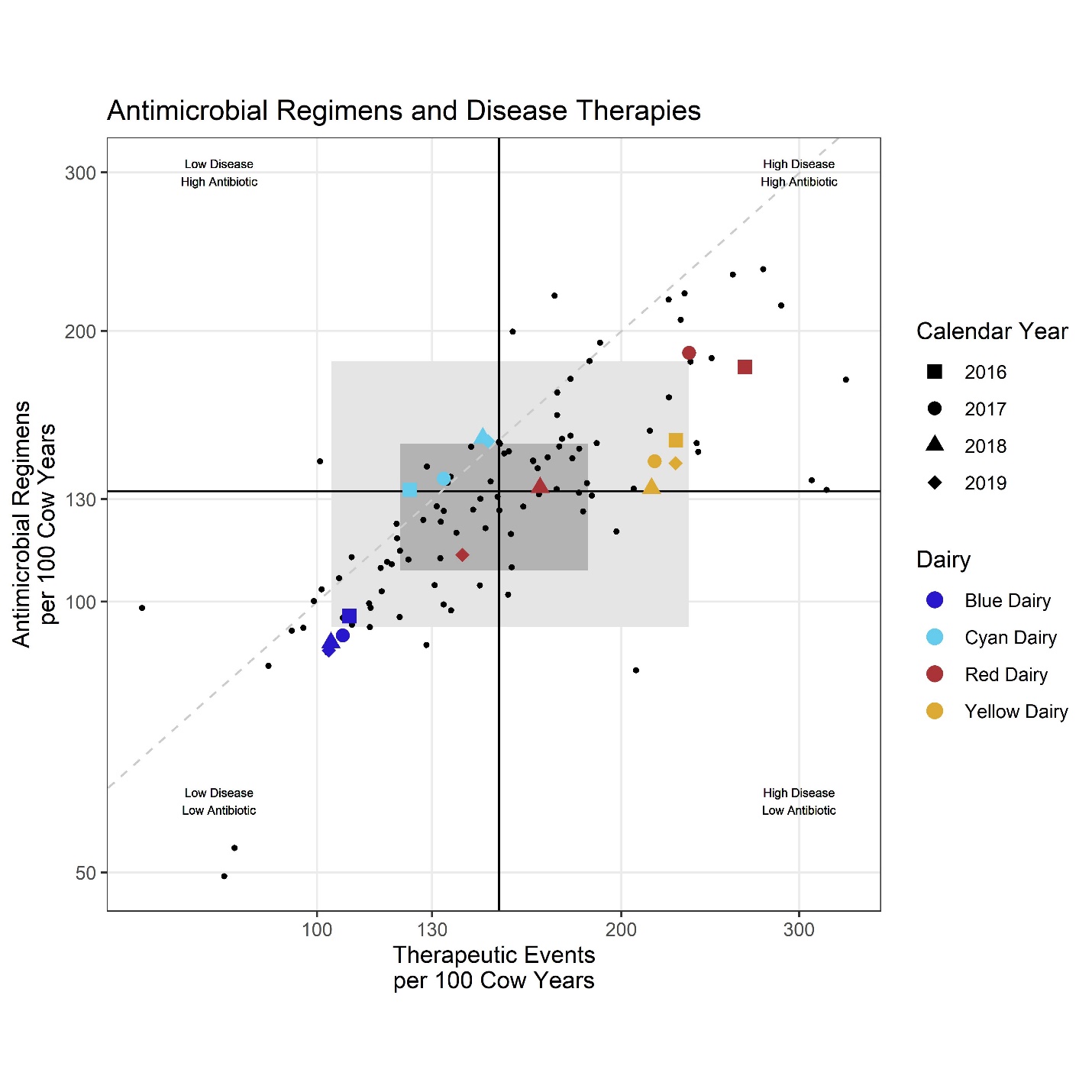
**

**Figure S4.12 (same as main paper Figure 2)**


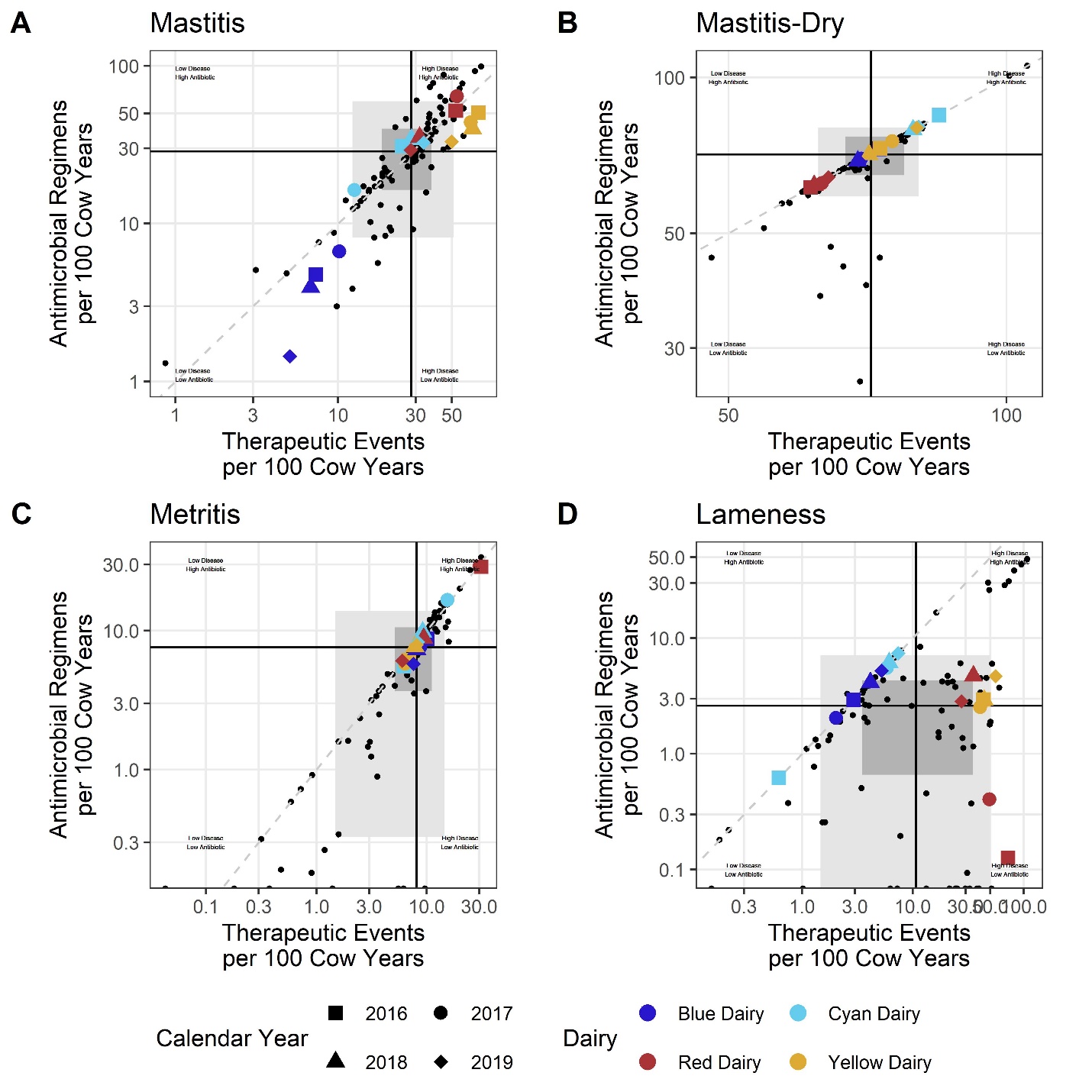


## 4.3 Scatter plots – Commodity Group or National Summaries

**Figure S4.13 (same as main paper Figure 3)**


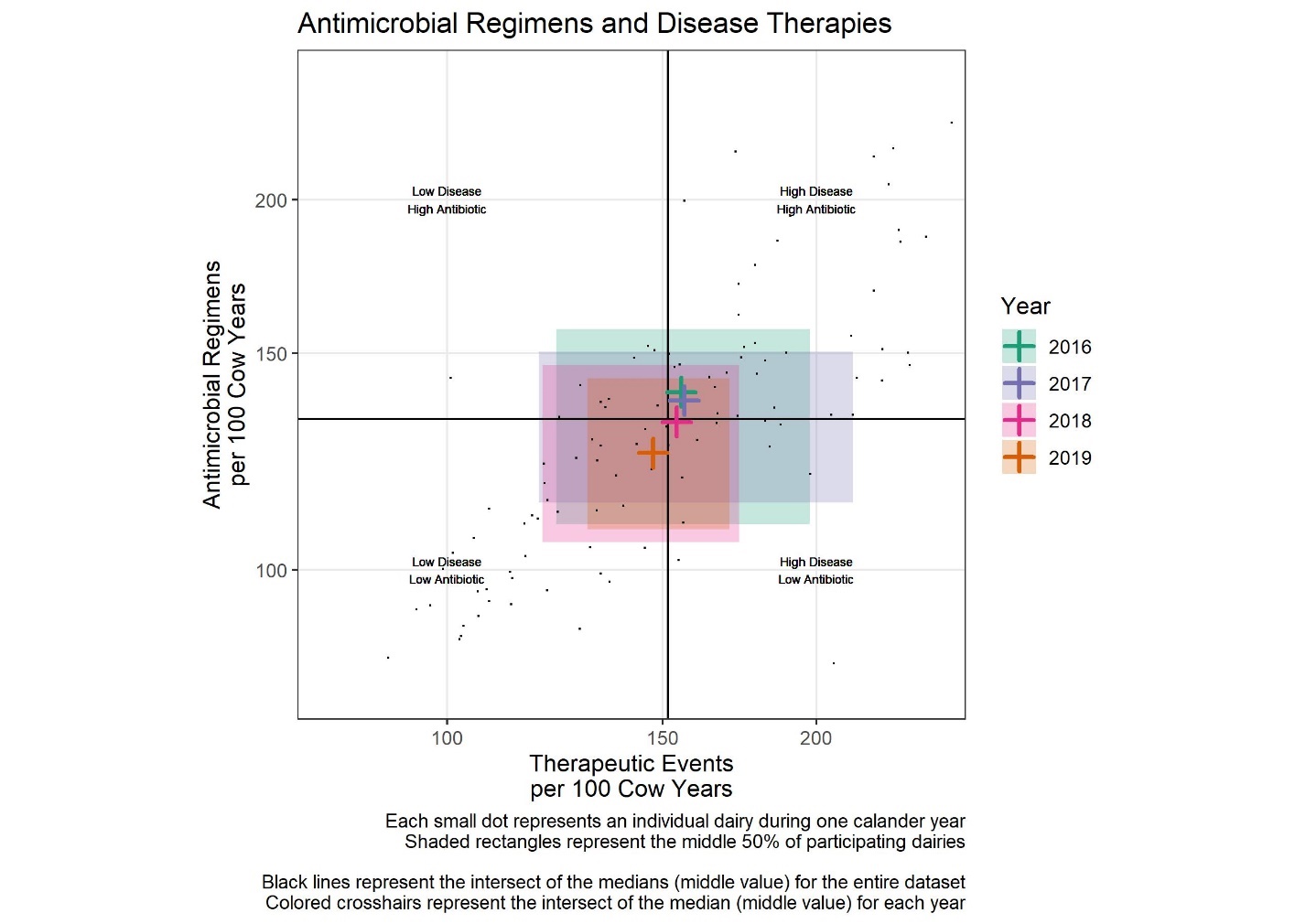


Data can be summarized over time across all dairies. Each colored shaded rectangle represents the middle 50% of values on each axis. The small colored cross hairs represent the medians for each axis within each year. Large black cross hairs represent the medians for each axis across all dairies for all years.

## 4.4 Tabular Output – Individual Farm

All 11 metrics calculated are presented in 3 sets of tables below: therapeutic metrics, therapeutic outcomes, and record details. Each table is subdivided by disease and calendar year. The numerical values within the tables are the value for the individual farm reported. The background color behind each value is mapped to the farm’s percentile rank for that value. Percentile rank is best interpreted as the farm’s “Benchmark” when compared to the other farms in the study.

The grey shaded values in the tabular formats indicate values for which no across farm ranking was performed. There are 2 reasons this occurred. First, no ranking was performed on the raw count of events (top row labeled “therapeutic event count”). Second, outcomes were not ranked if the total number of therapeutic events was less than 30 within any category as this percentage is not readily interpretable due to the large magnitude of effect that one individual cow might have.

Some farms have a small percentage of outcomes classified as “Unknown”. This occurs when there are data challenges surrounding cow identity, and the outcomes could not be calculated. This happens more frequently on farms with hand written records due to the increased likelihood that a cow number might be miss transcribed, or when cow numbers are re-used in a subsequent year.

**Figure S4.14**


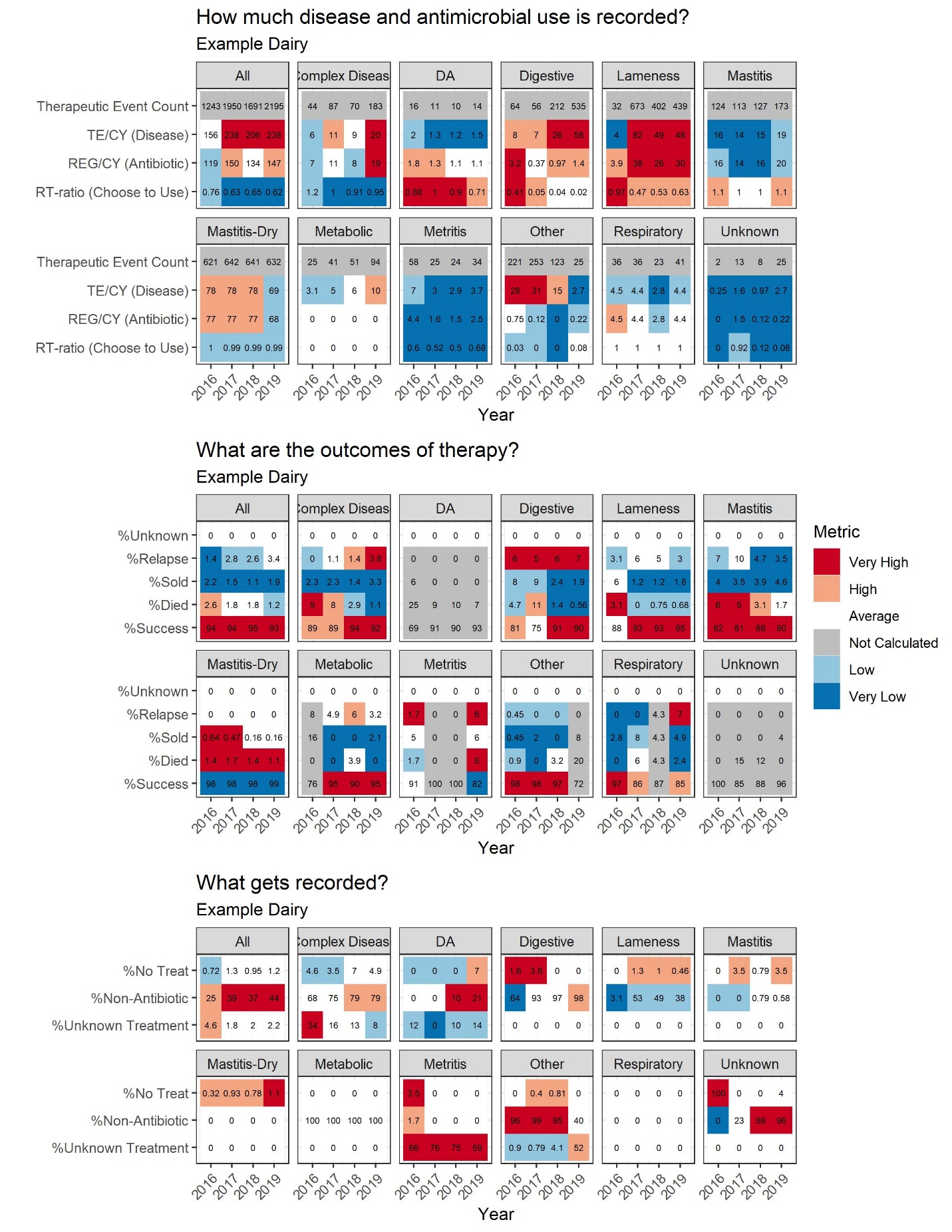


# 5. Example Farms - Original Benchmark Reports

The following are benchmark reports for the example farms: Red Dairy, Cyan Dairy, Blue Dairy, Yellow Dairy.

The reader is encouraged to view section 4 of this supplemental material labeled “Training figures for graphics” prior to viewing these benchmarks.


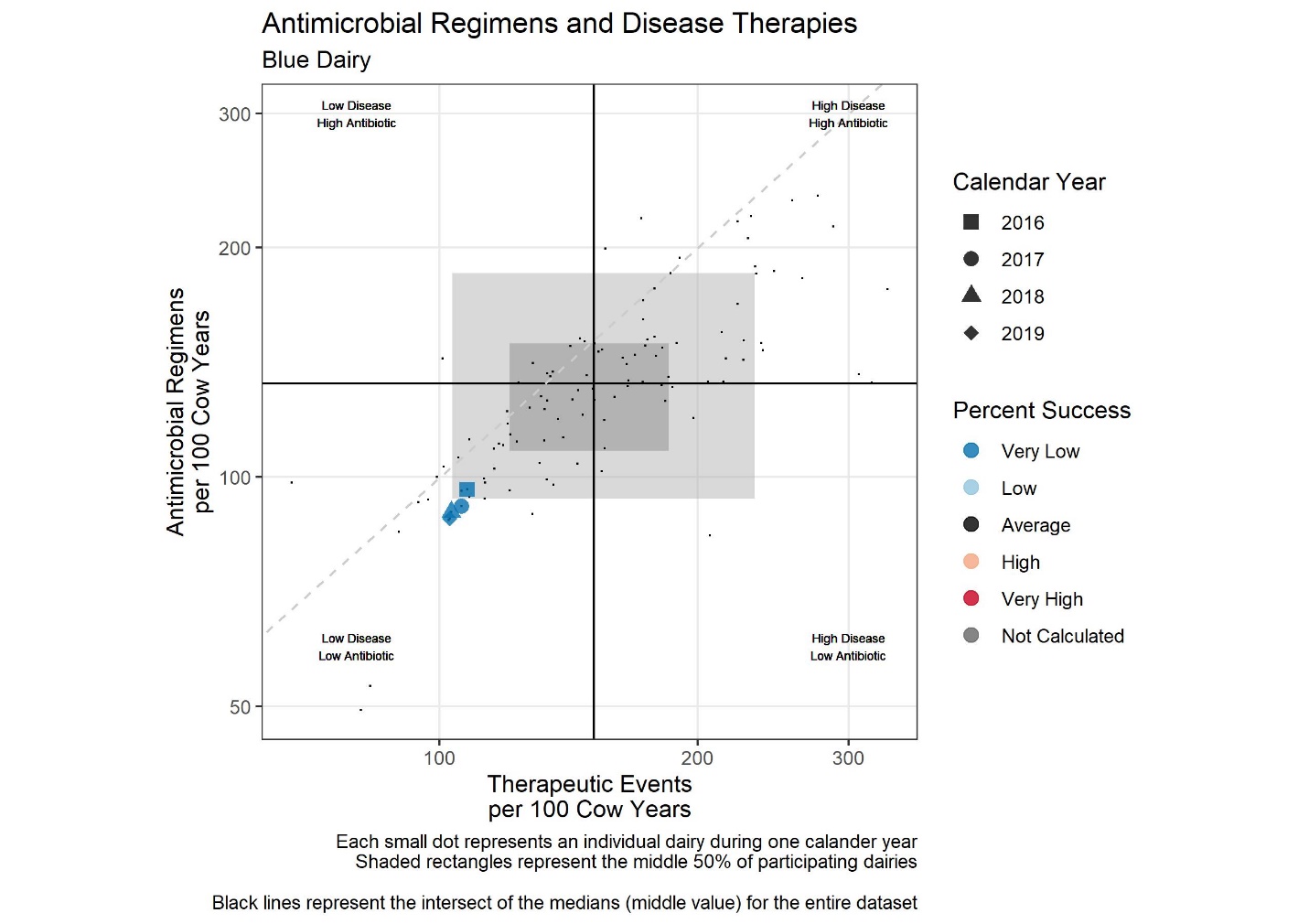

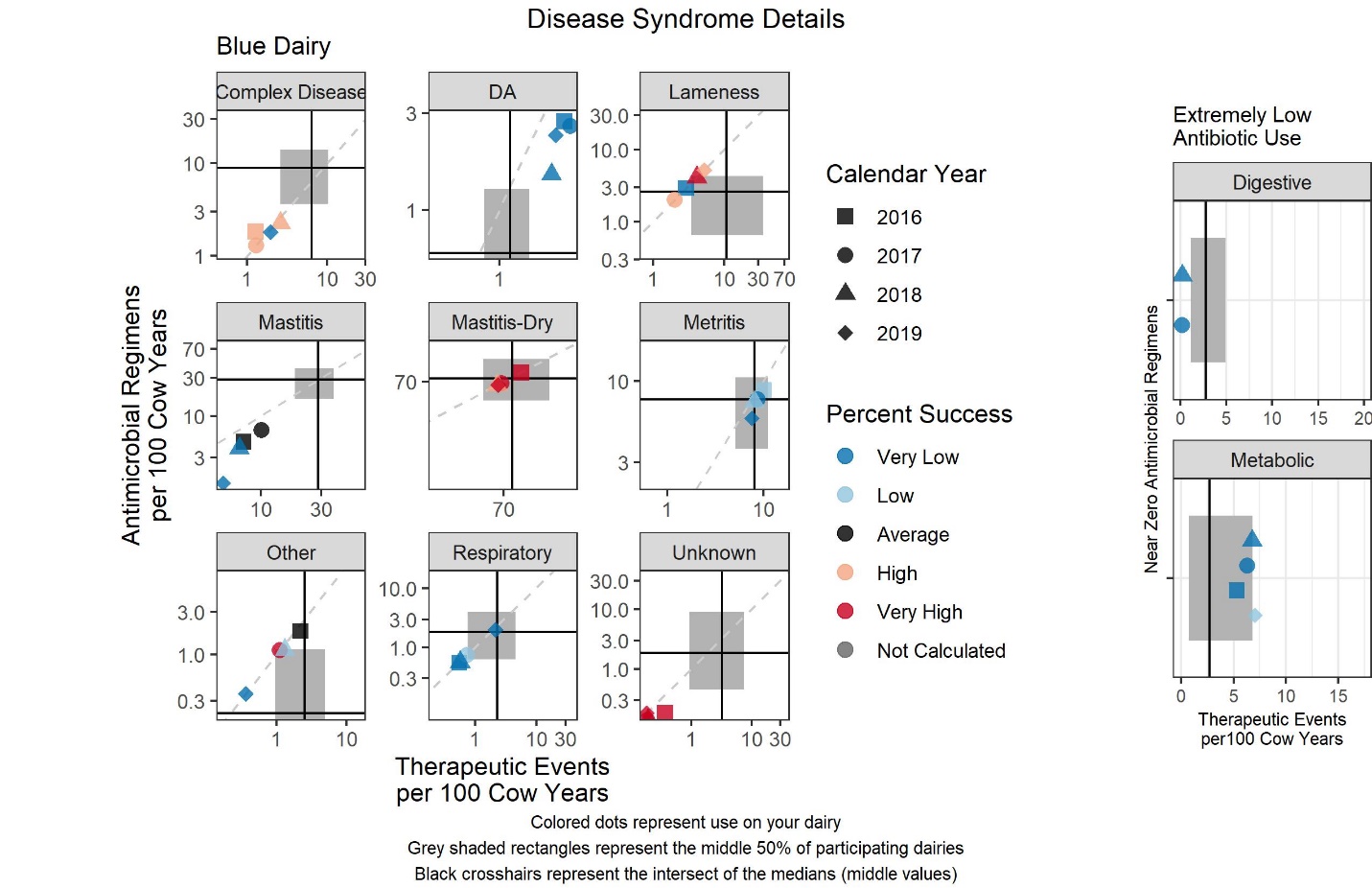

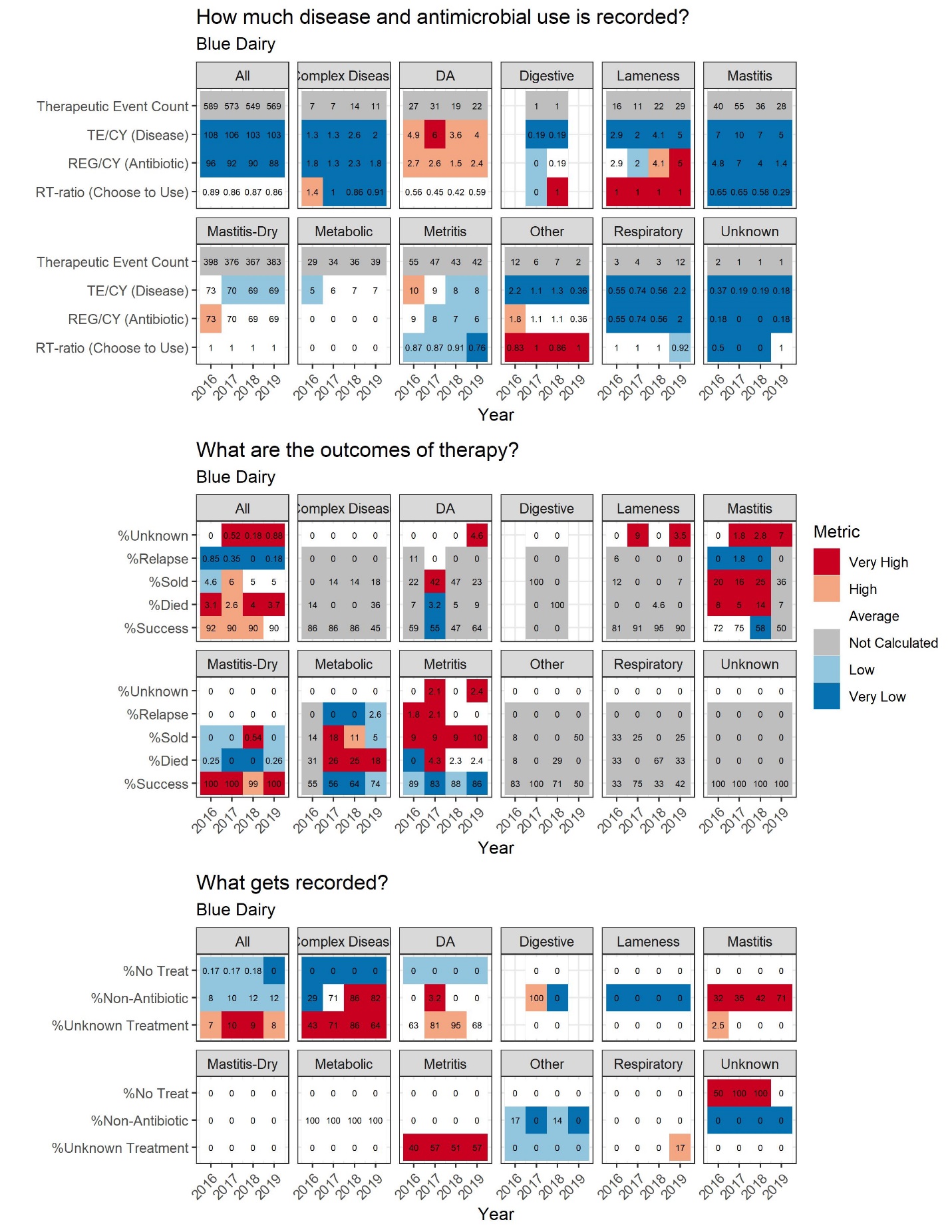


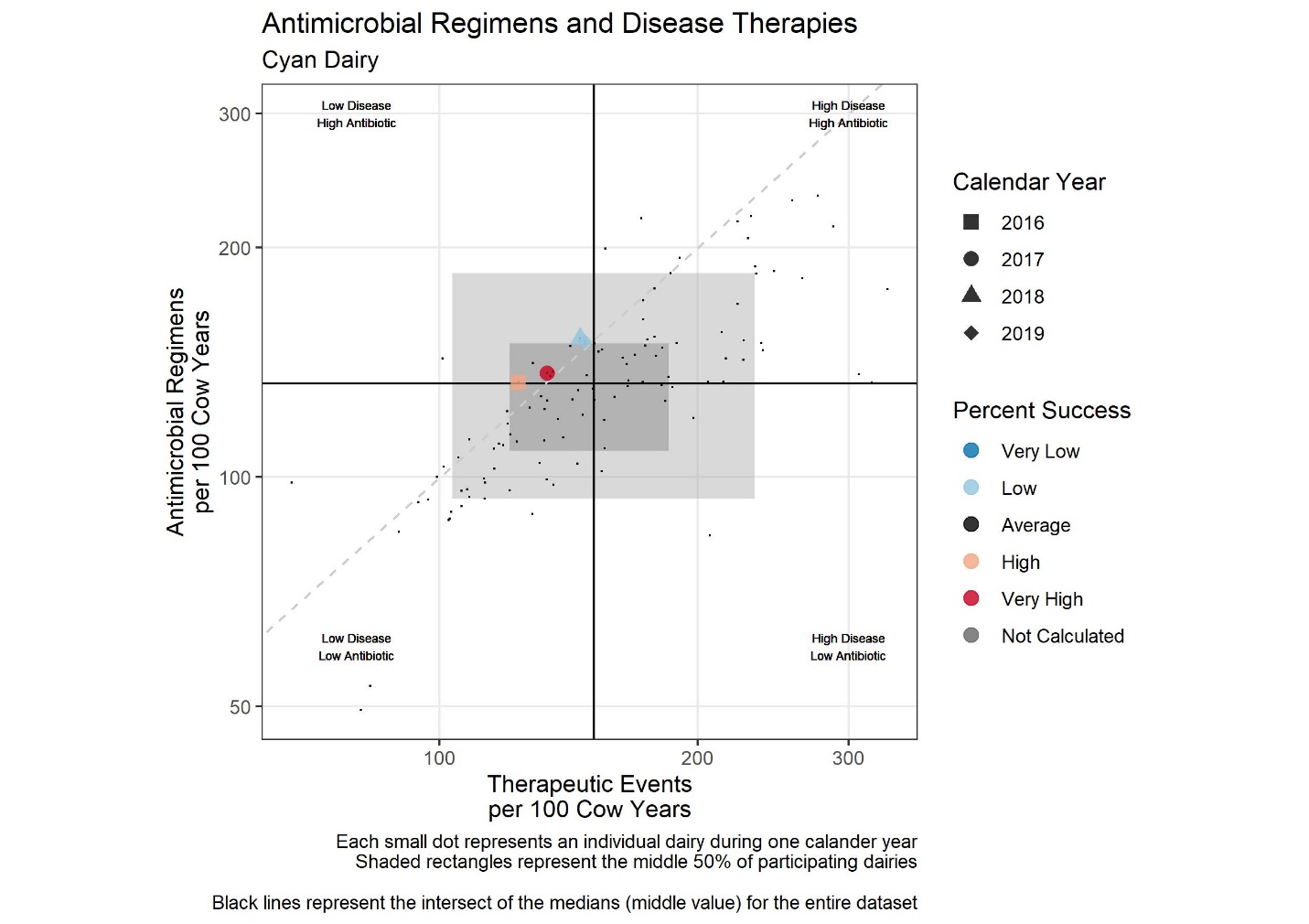

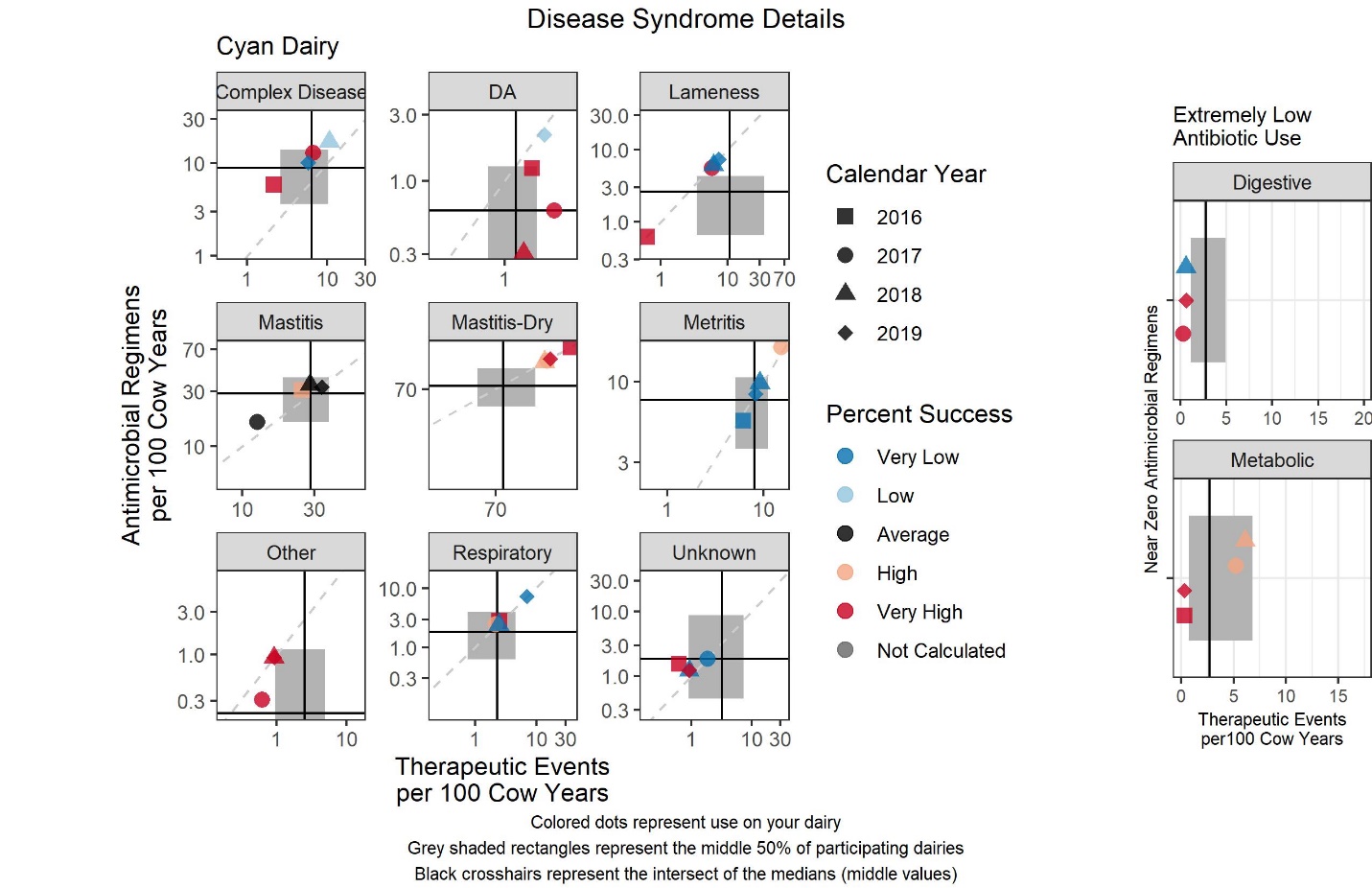

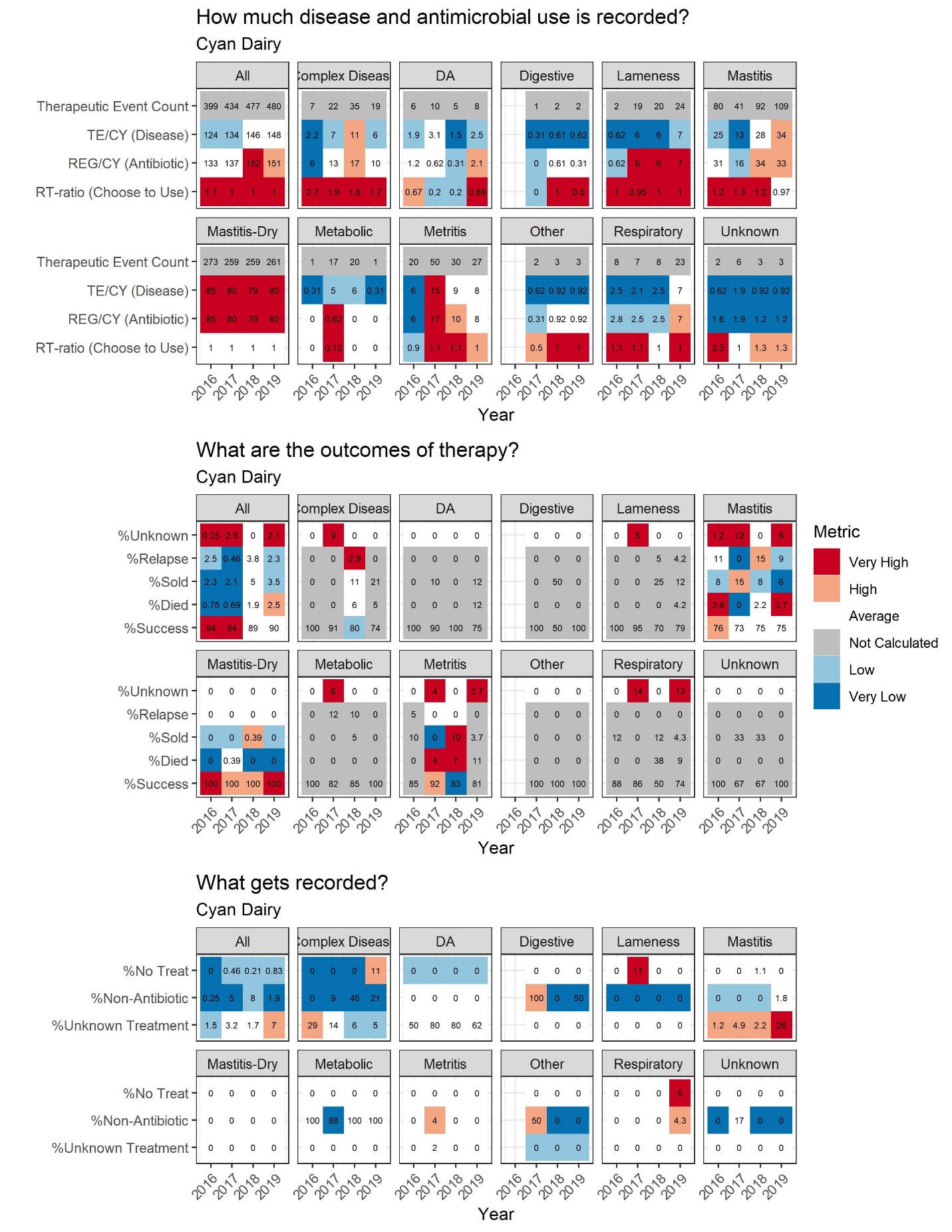


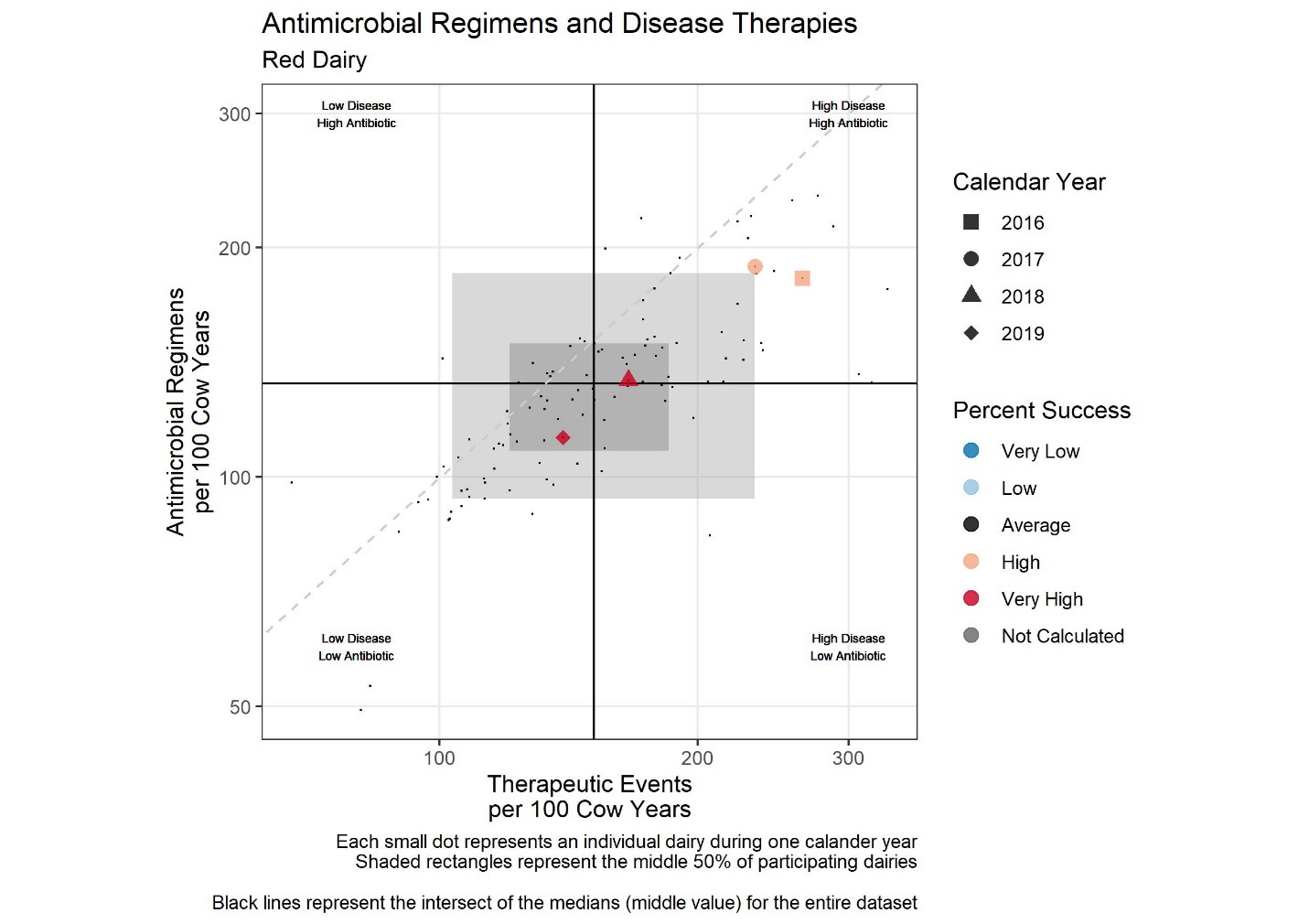

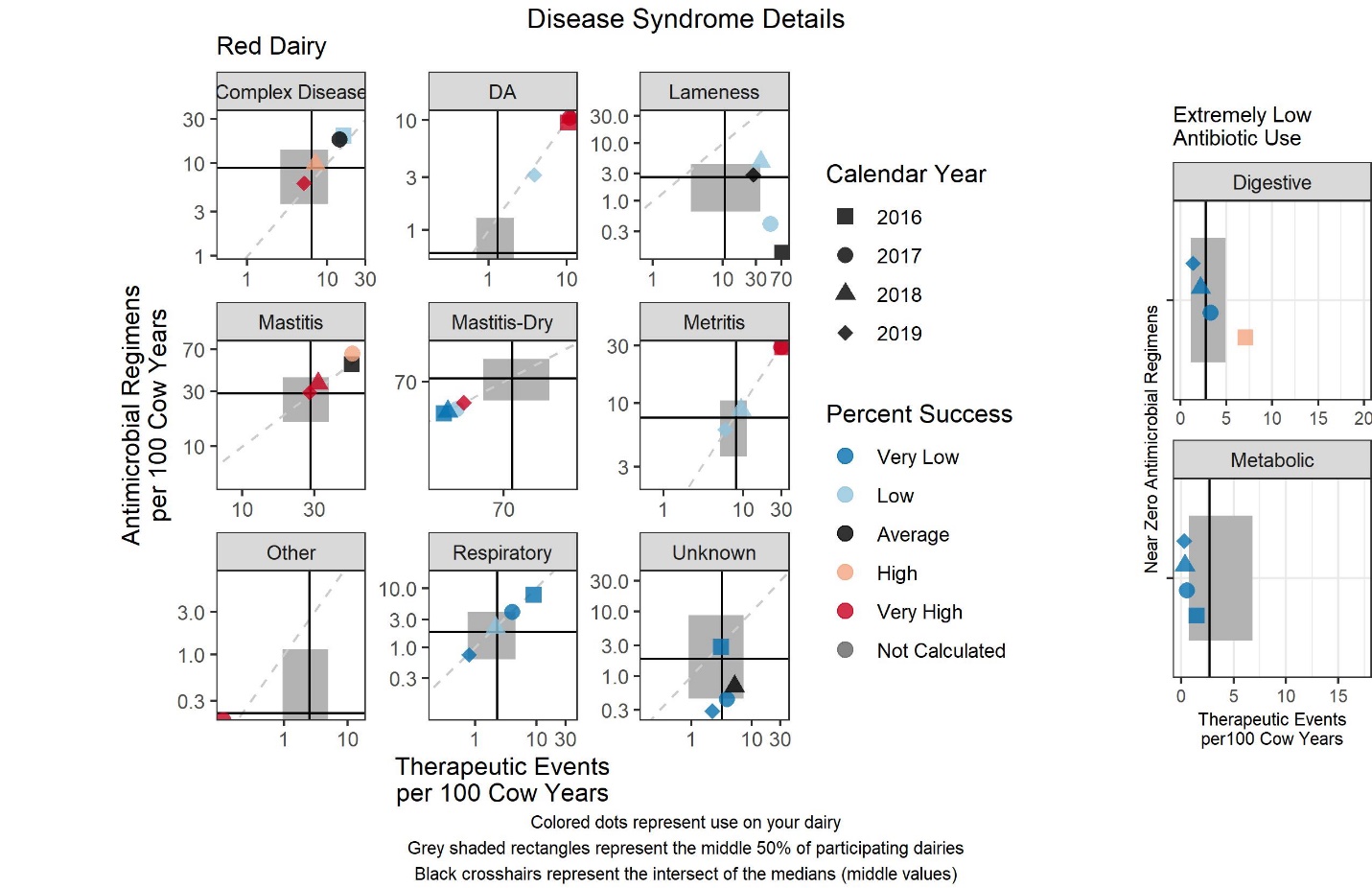

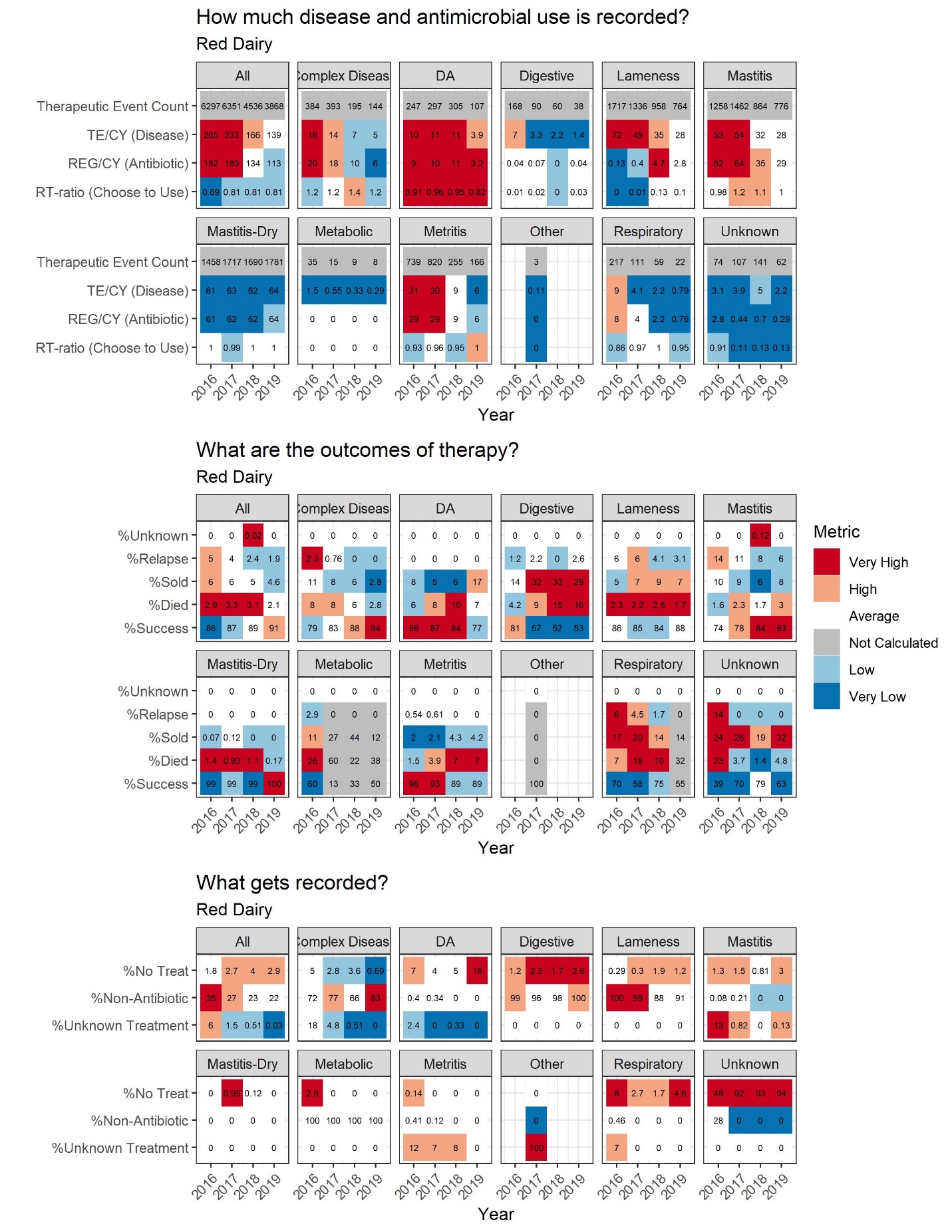


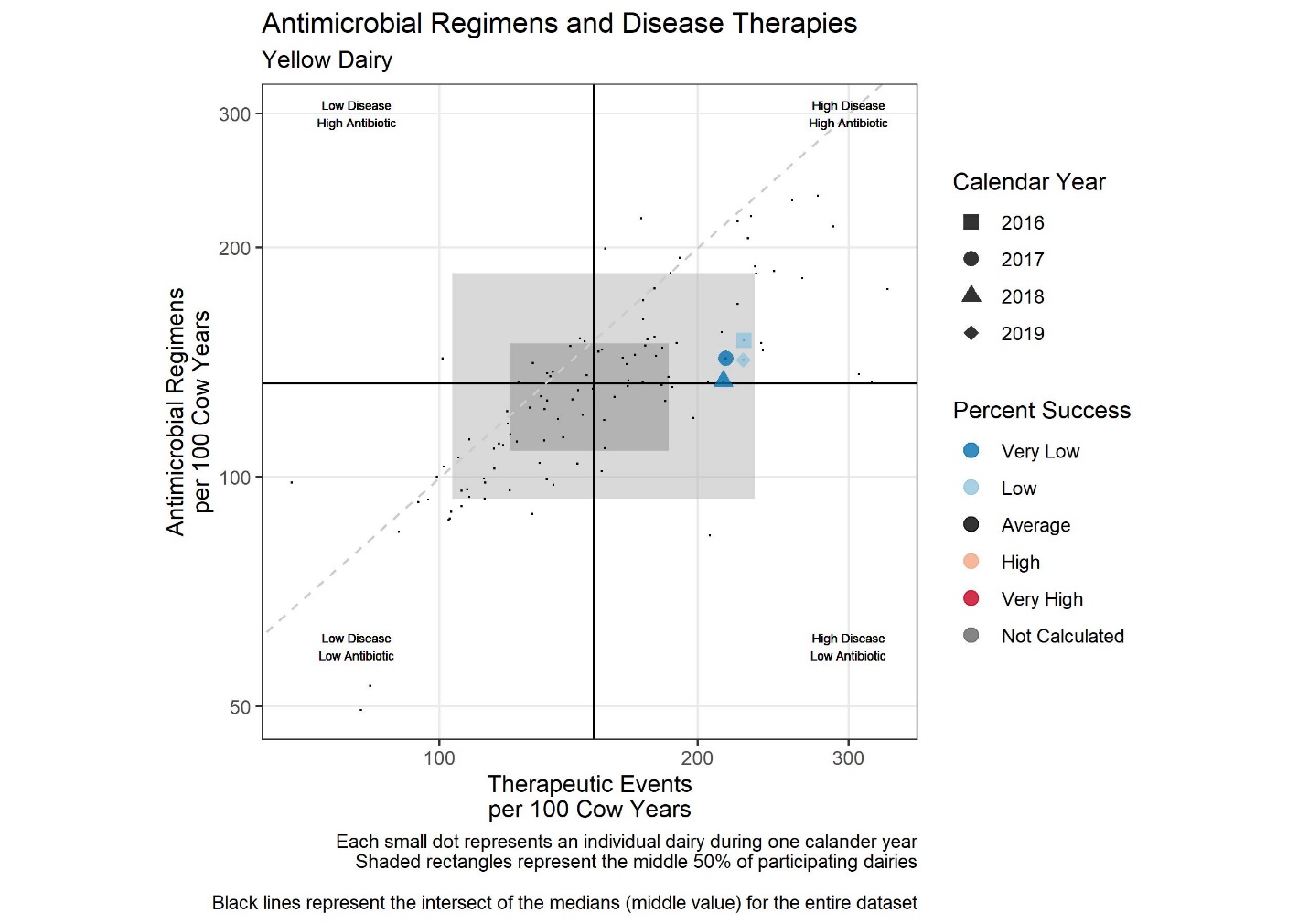

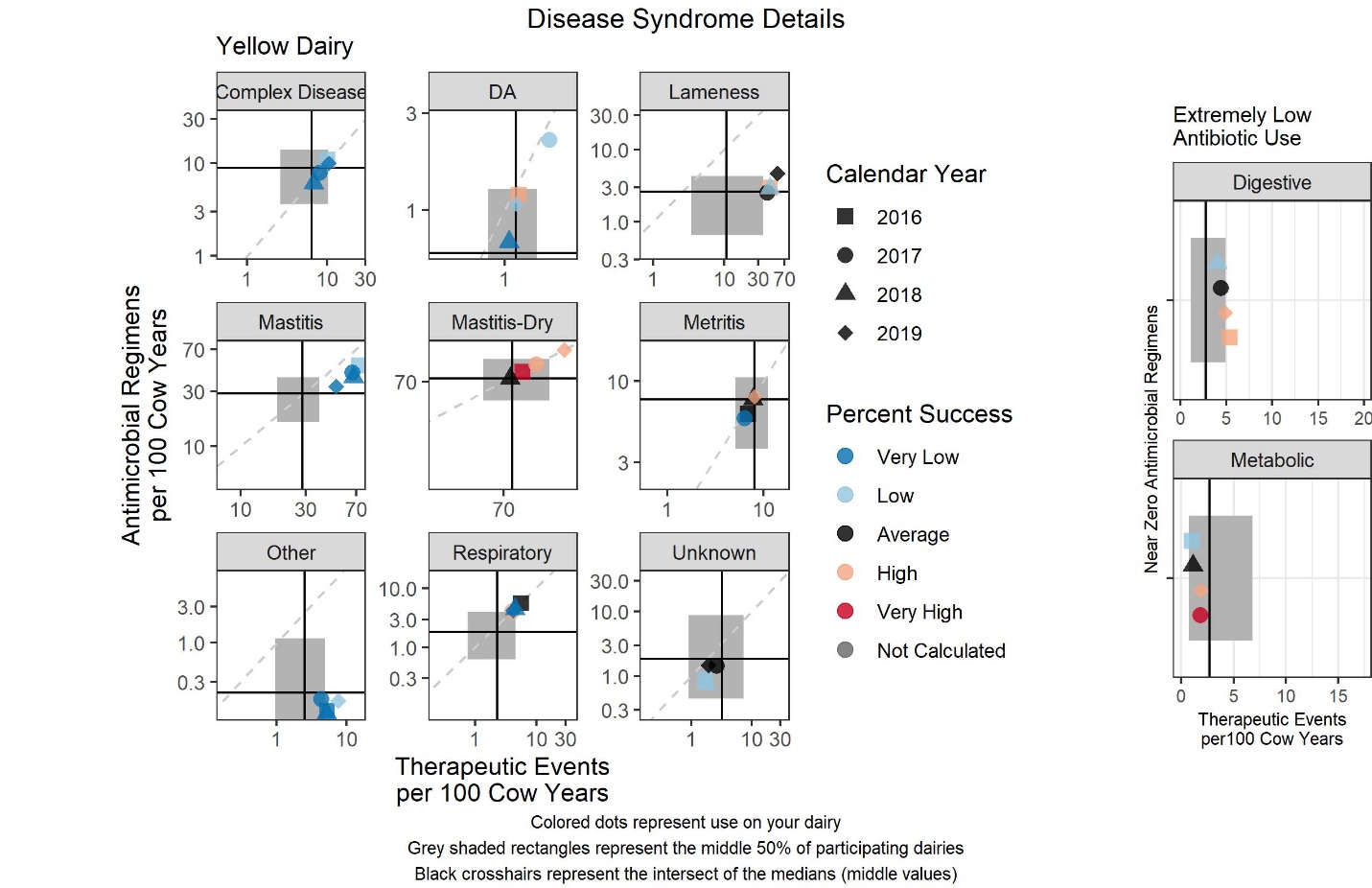

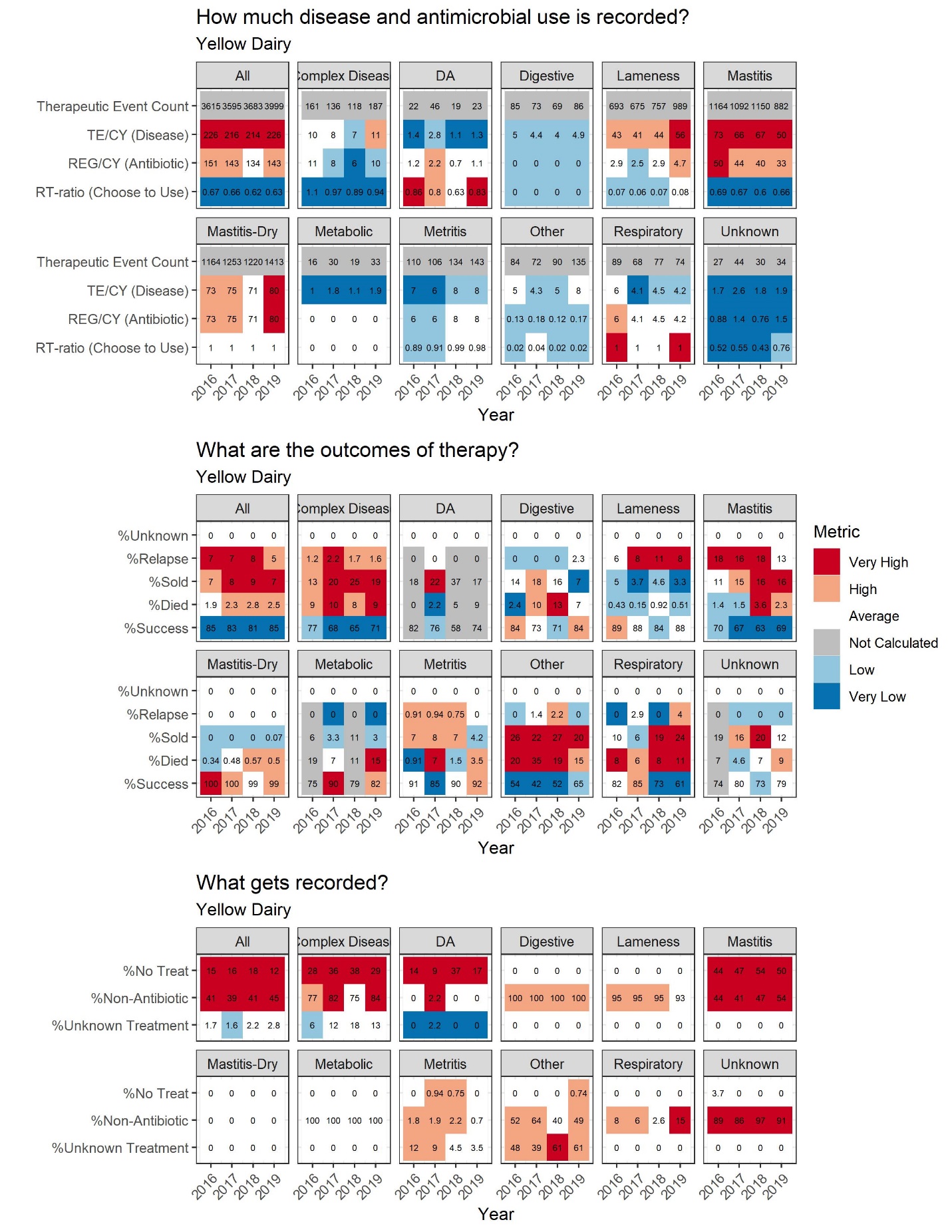


# 6. Summary of Study Antimicrobial Use

This section summarizes antimicrobial use across all 27 participating farms over 4 years by 3 metrics: Calendar days of administration per cow year (**CDoA / CY**), Grams per cow year (**Grams / CY**), and Regimens per cow year (**REG / CY**). This convenience sample should not be interpreted as representative of use across the entire US dairy industry. It is presented simply to demonstrate that the method for creating farm level benchmarks described as useful for actionable stewardship at the farm level, can also serve to summarize use across multiple farms. For a discussion of how these metrics relate to each other please refer to Schrag et al. DOI:[10.1111/zph.12772](https://doi.org/10.1111/zph.12772)

It should be noted that regimens per cow year and grams per cow year were published previously for years 2016 and 2017. However, there are several important differences between those reported metrics and the ones reported here.

- 2 dairies previously reported for years 2016 and 2017 failed to meet data quality standards for years 2018 and 2019 and are therefore excluded.
- The previous report calculated all metrics per cow year. This report calculates all metrics per 100 cow years.
- In the first report, systemic use for mastitis was distinguished from intramammary mastitis use and dry cow therapy. In this report, treatment for mastitis at the time of dry off is reported separately, but all other treatments for mastitis are combined in the mastitis category, regardless of whether they were systemic or intramammary.
- In the report of the first 2 years the method of defining regimens required significant manual input for each dairy. An R function was written to automate much of this process, and then applied to data for all 4 years reported here.
- In the first report a “study level” value was calculated. The value of this is that each cow is weighted the same regardless of dairy size. Total cows represented per year ranged from 31,458 to 34,241. In this small convenience sample of 27 dairies, one dairy contributed 6392-6719 (19.0-20.6 %) of the cows per year. It was decided that this individual farm has too much influence on a study level value of a non-random sample, and therefore study level is not reported.

Figures are presented below.

Tables representing the values in these figures can be found here:

<https://docs.google.com/spreadsheets/d/1YeIGDPngpneLBGm-SILc6HESASgmMITX/edit?usp=sharing&ouid=112677804074355500543&rtpof=true&sd=true>

File name: Benchmarking Supplemental Metric Summary Tables_037.xlsx

## 6.1 Use by Disease

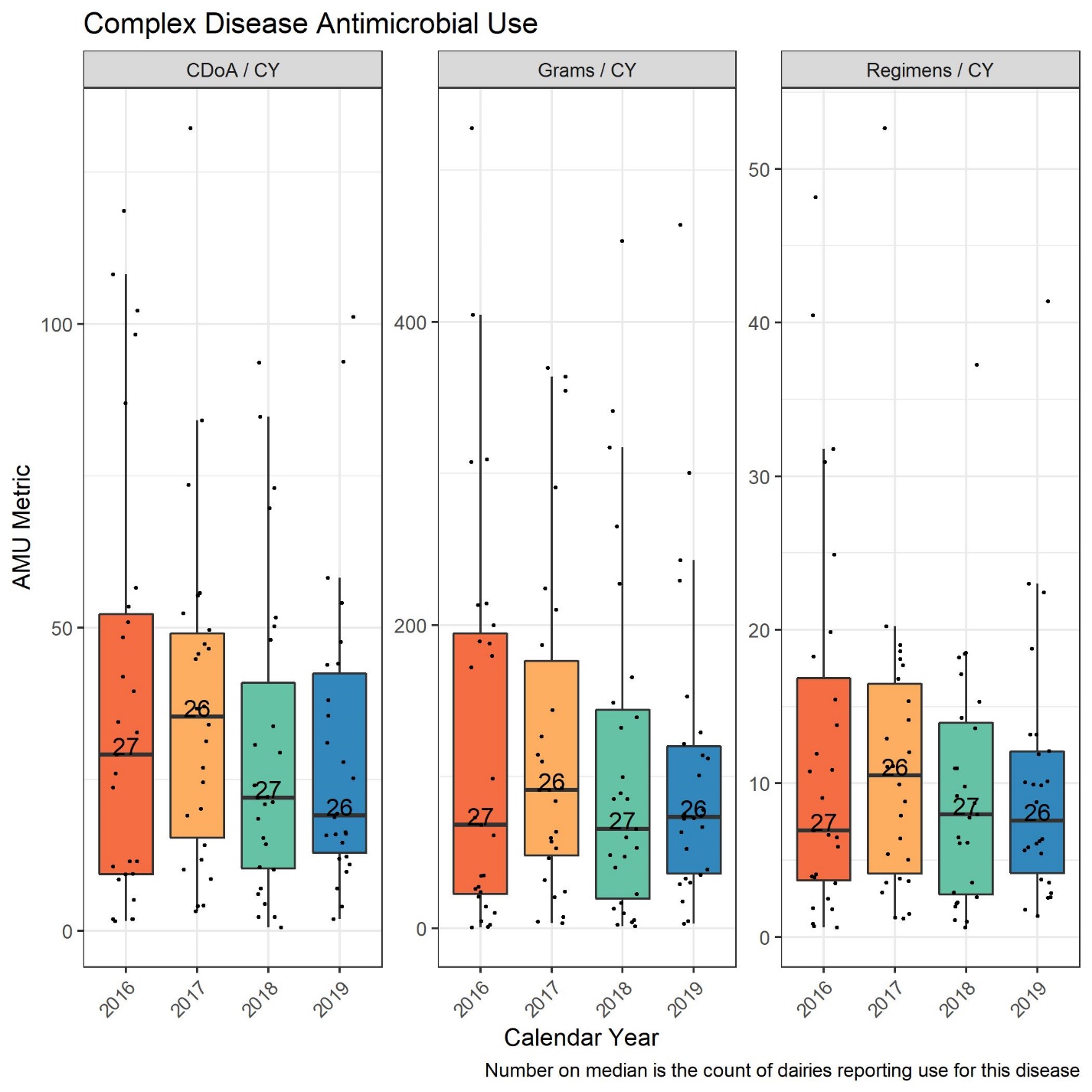

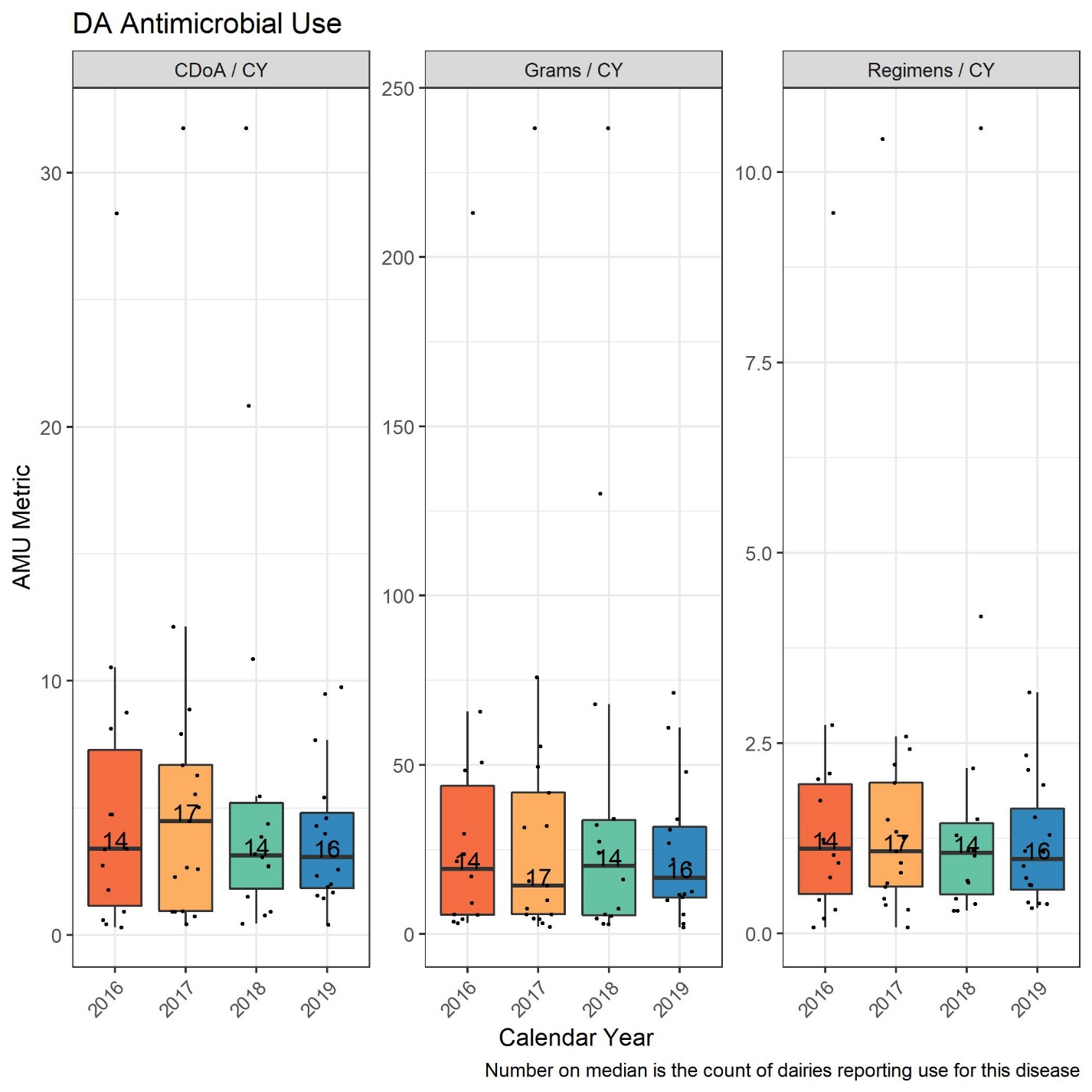

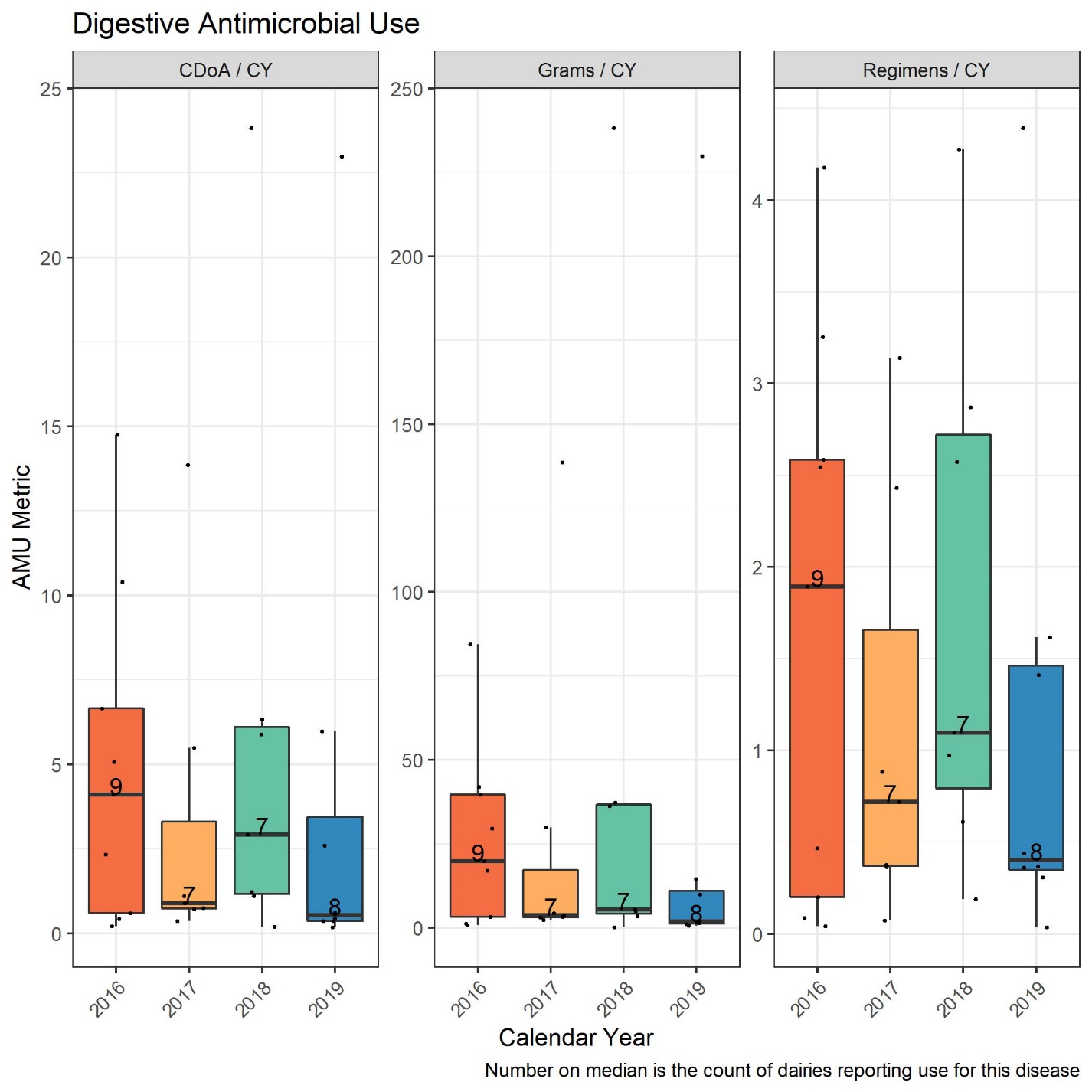

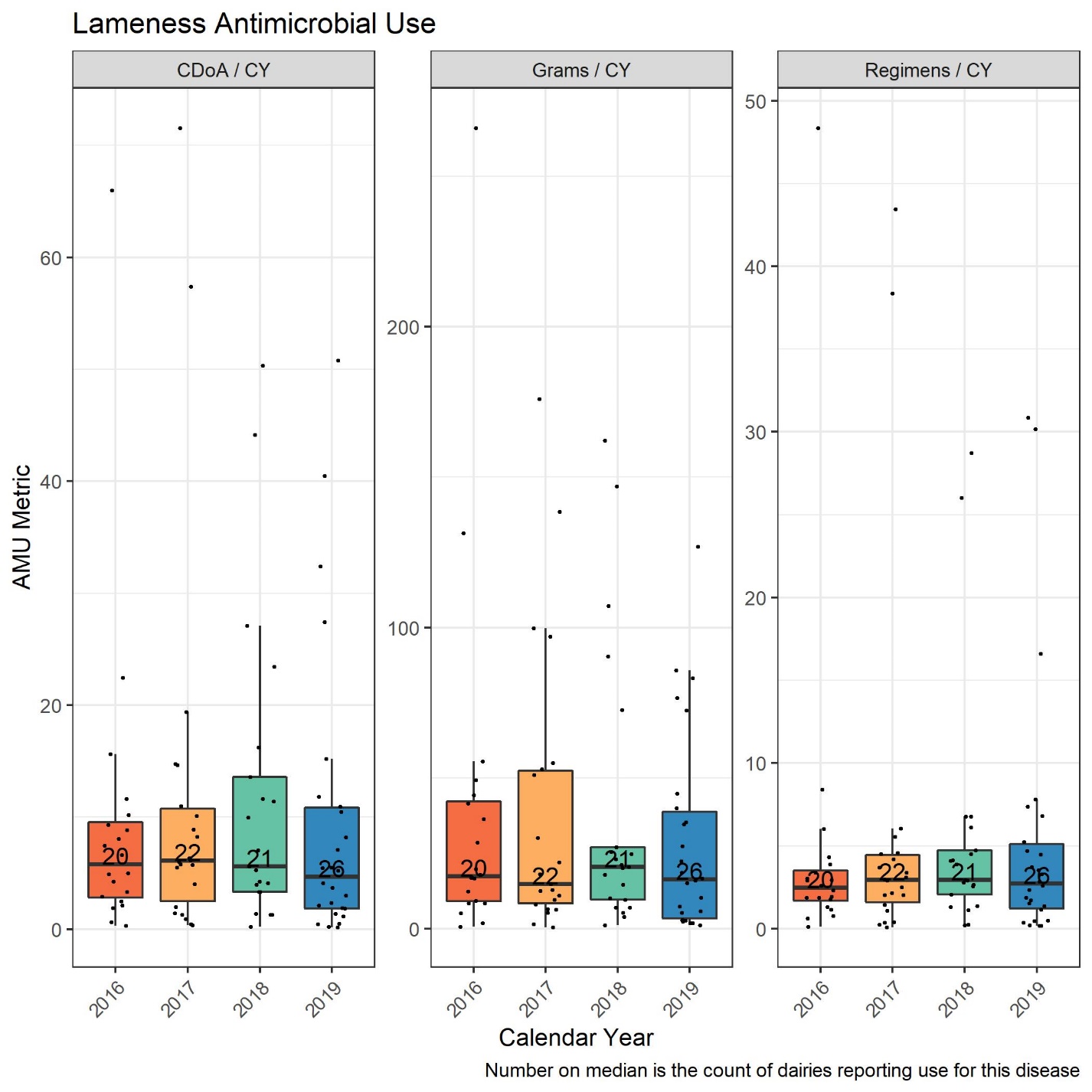

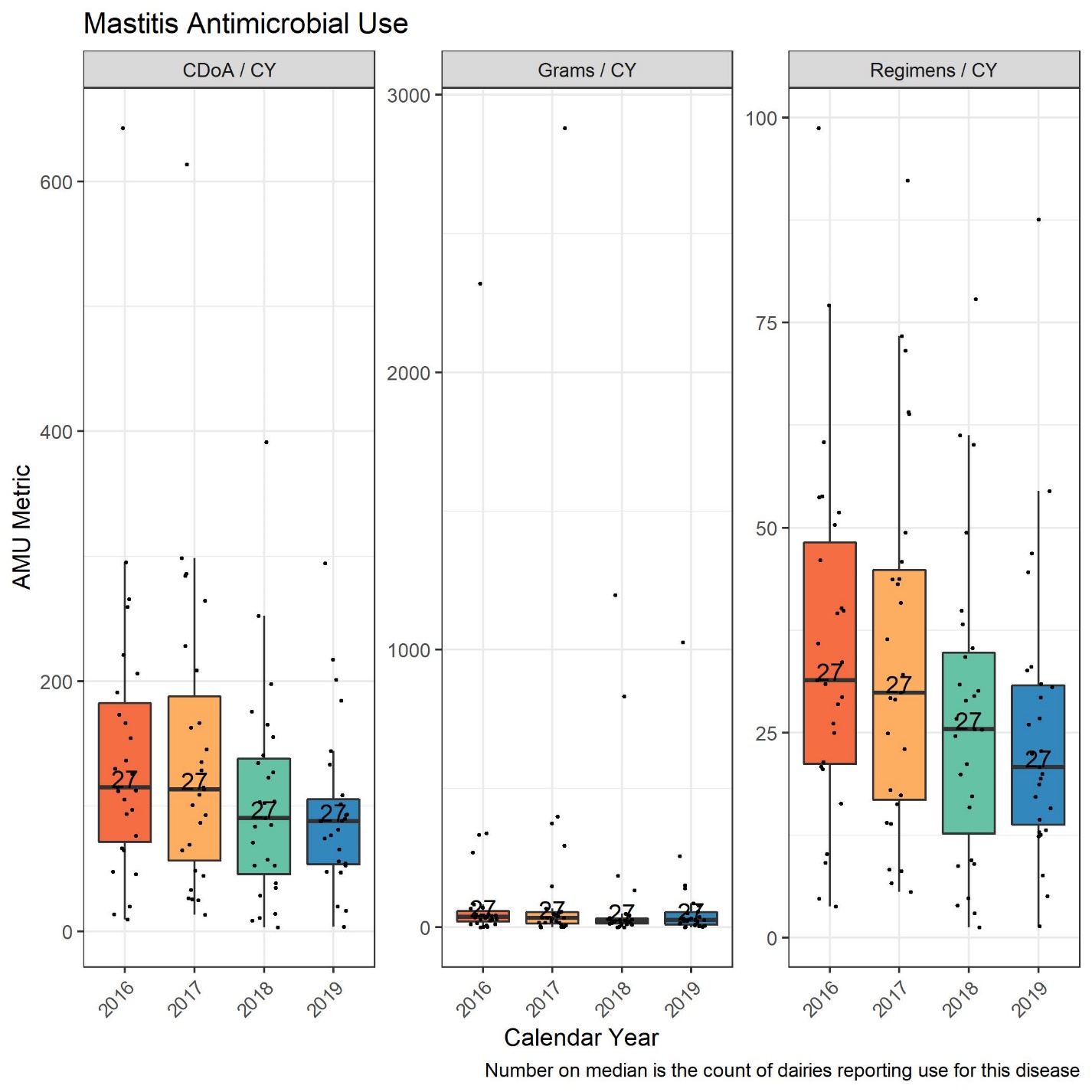

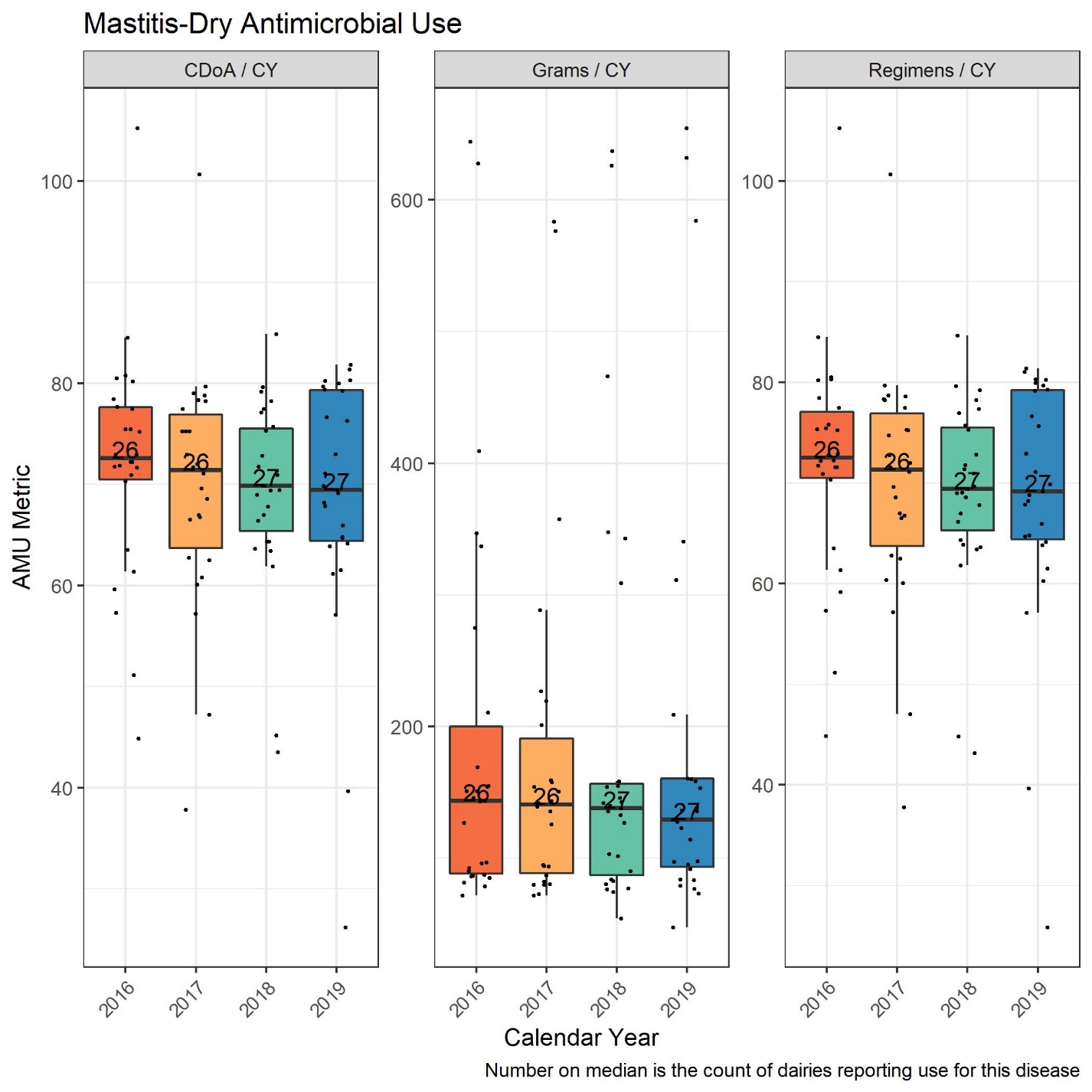

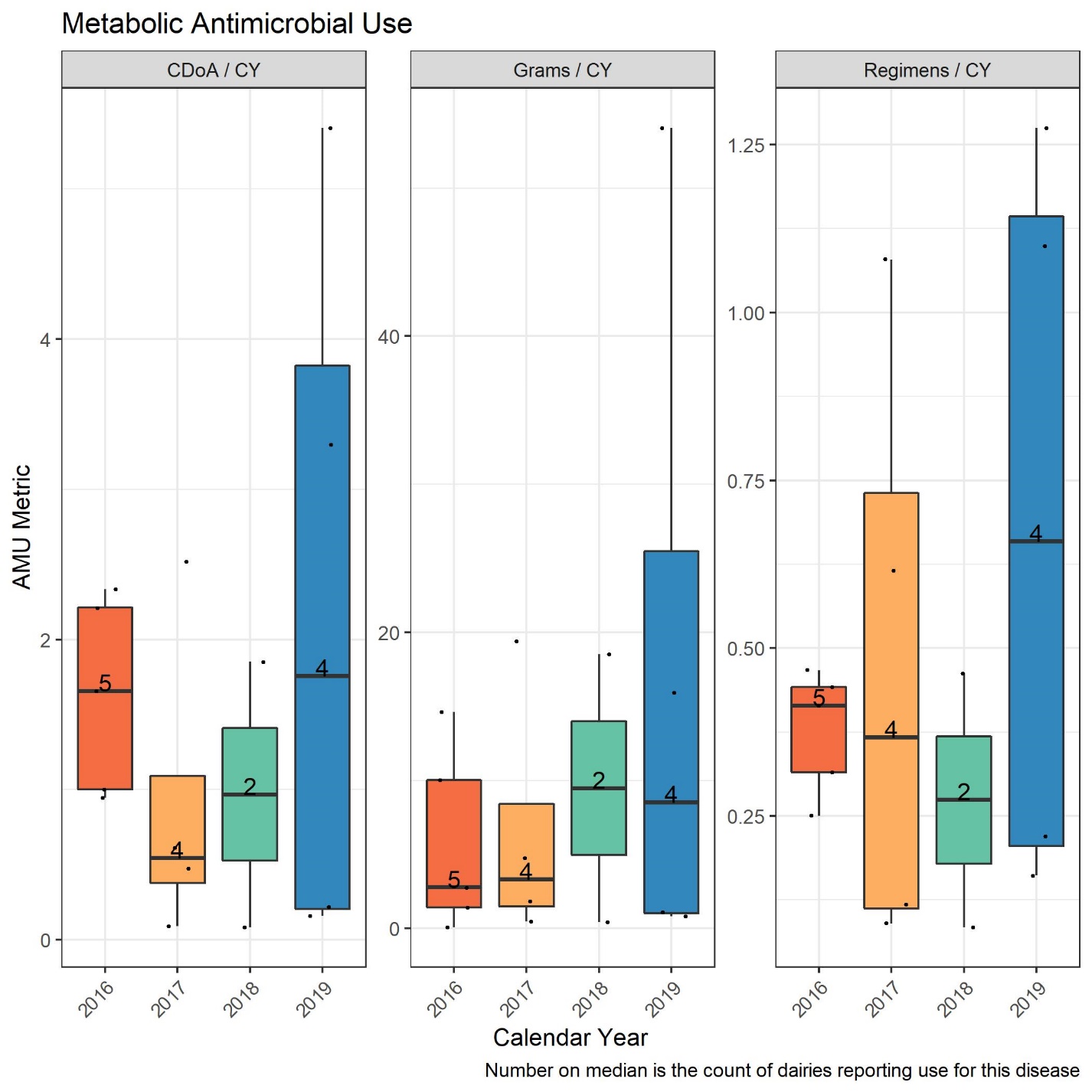

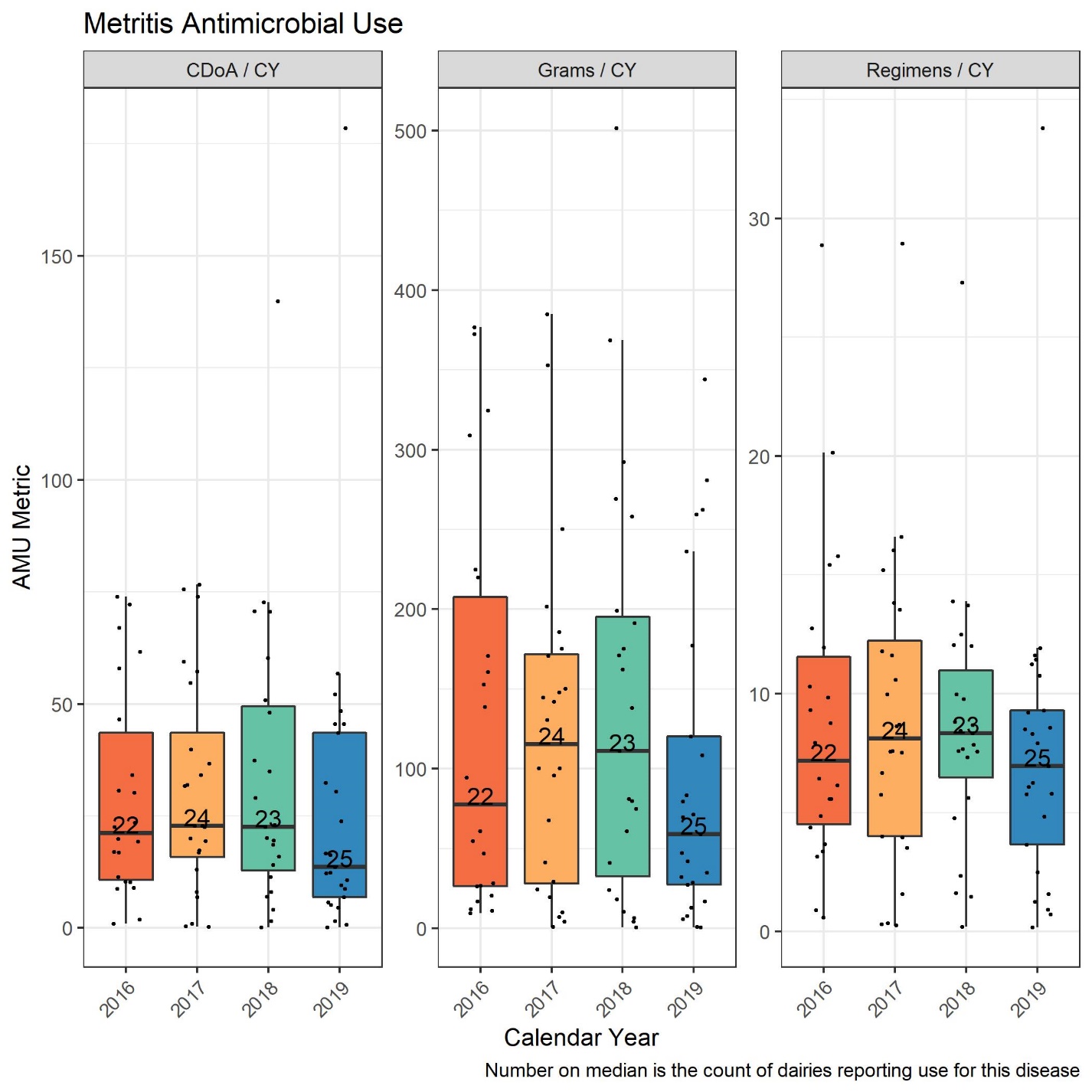

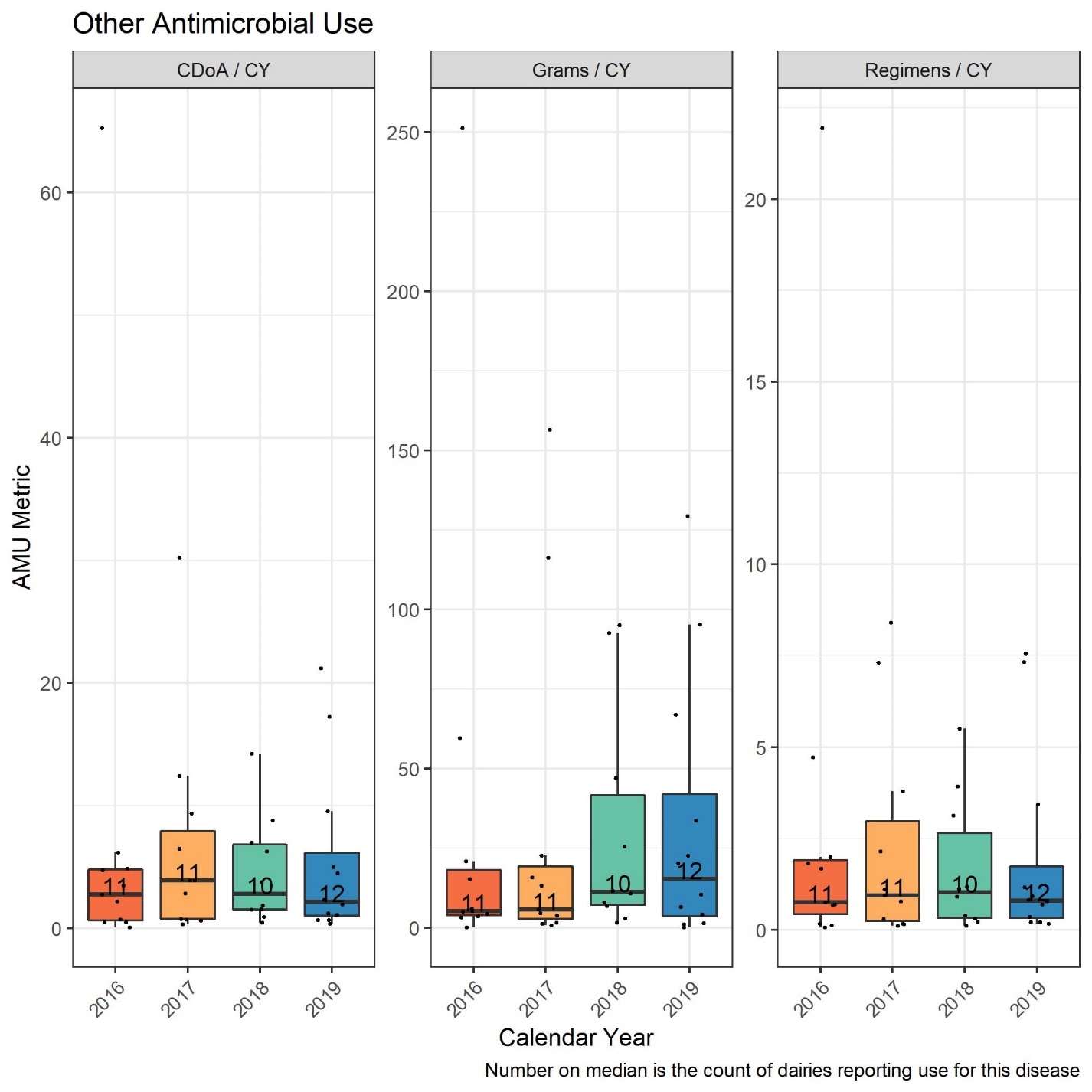

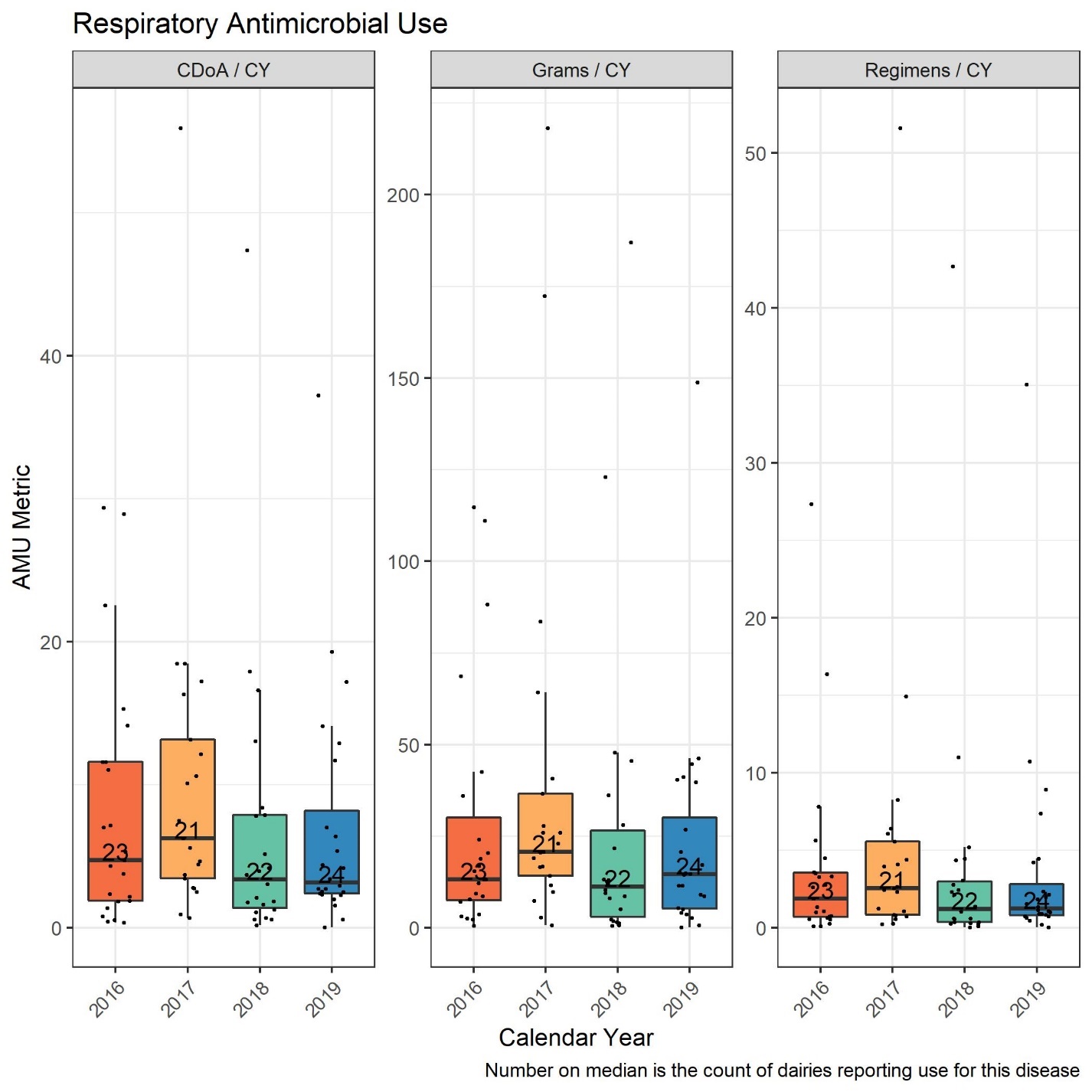

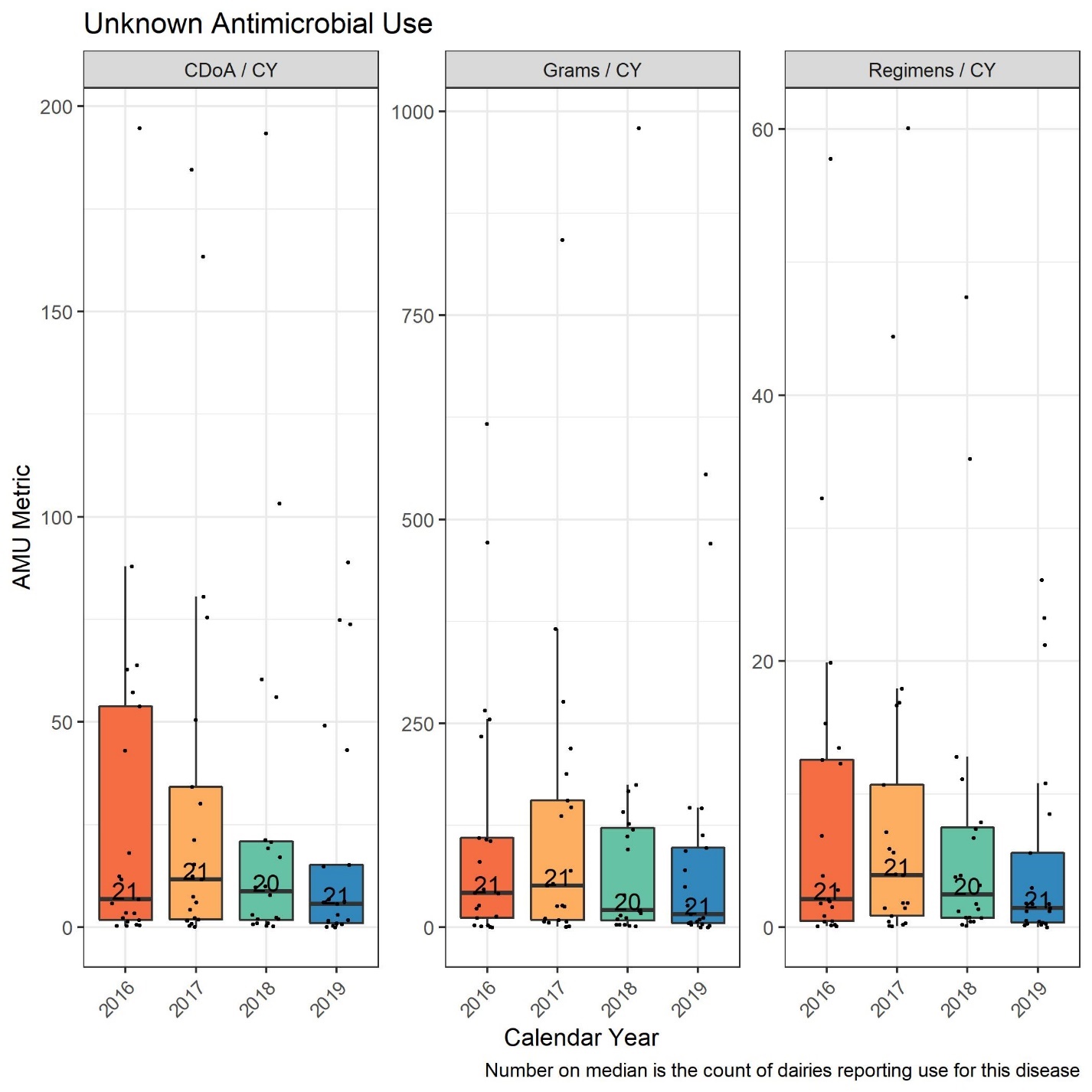


## 6.2 Use by Antimicrobial Class

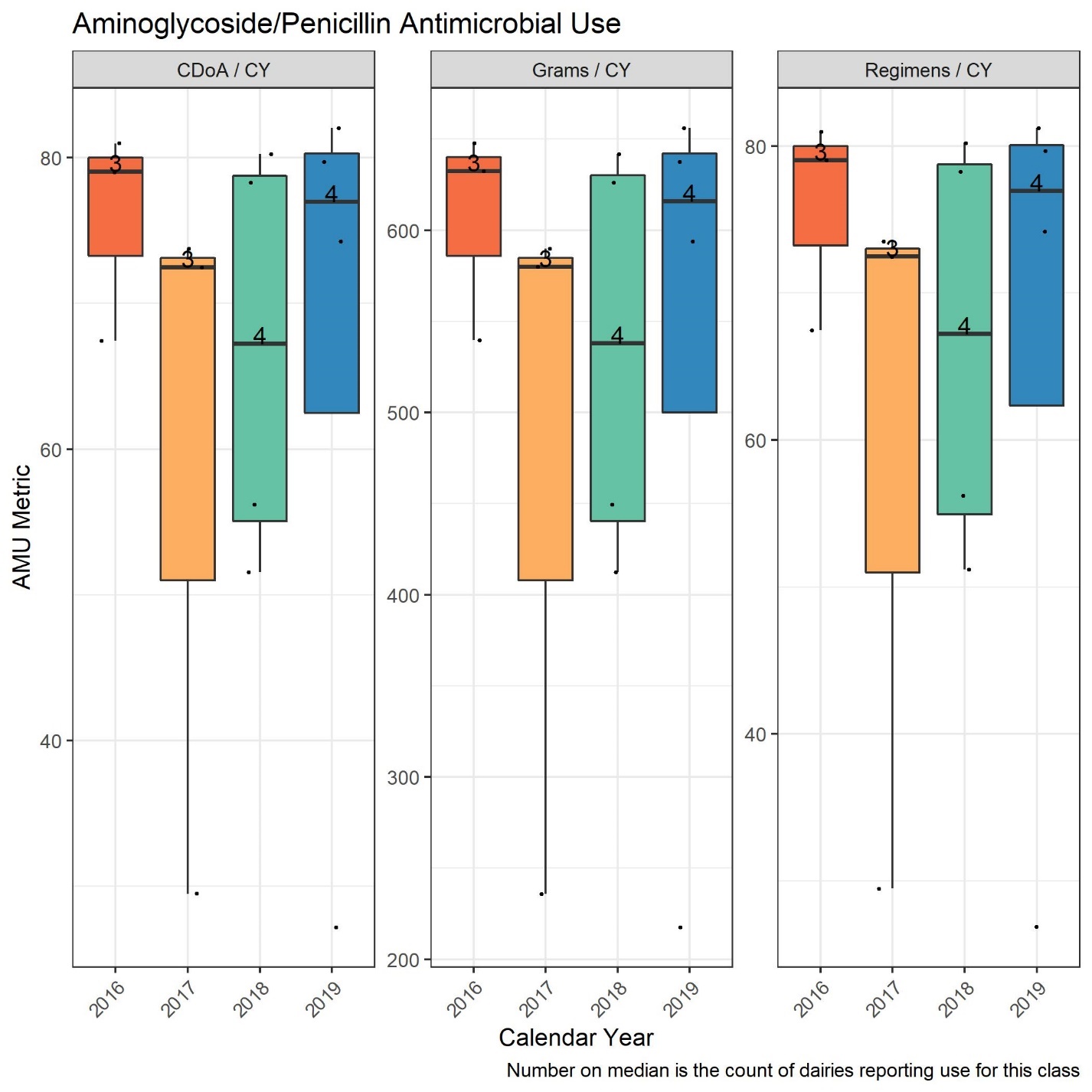

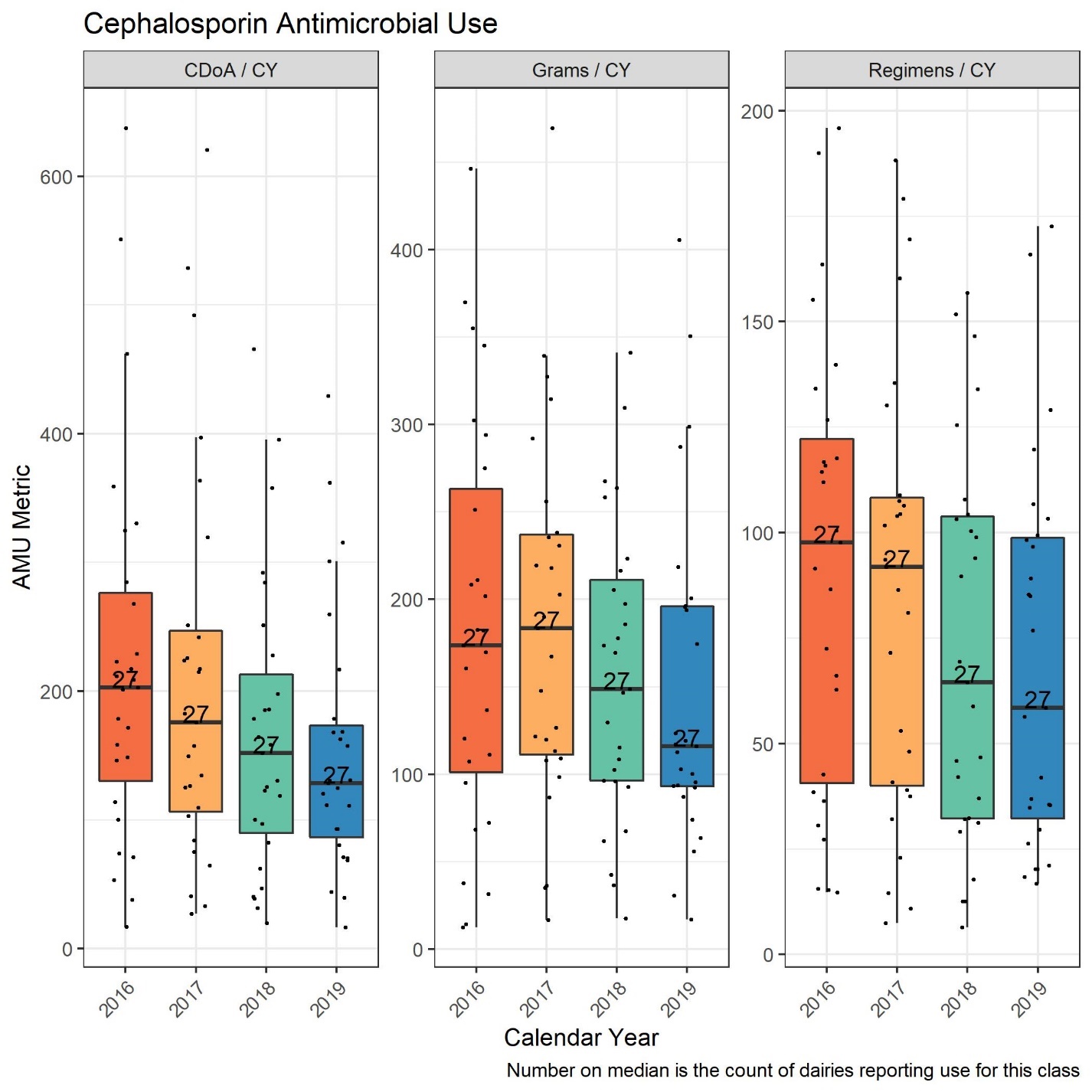

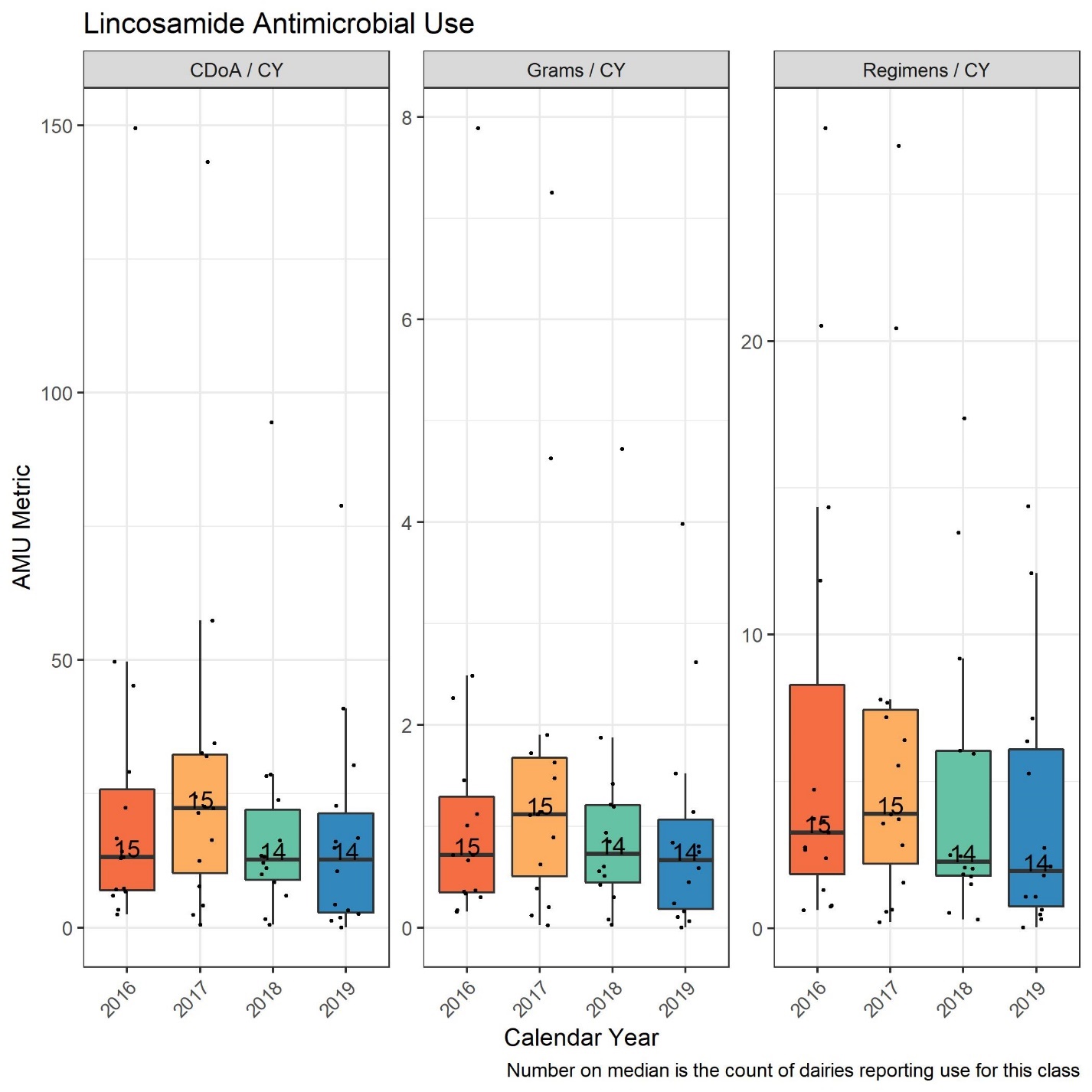

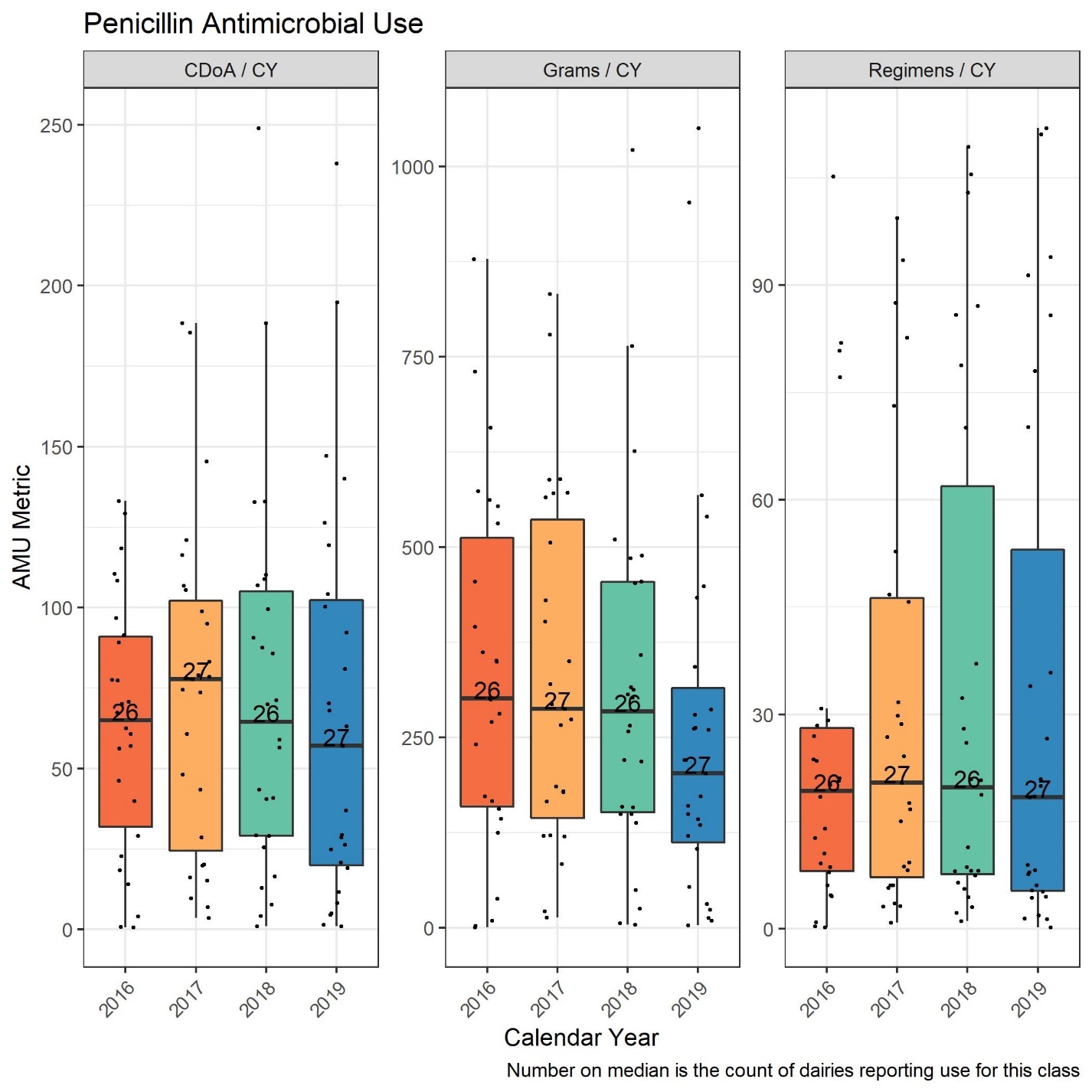

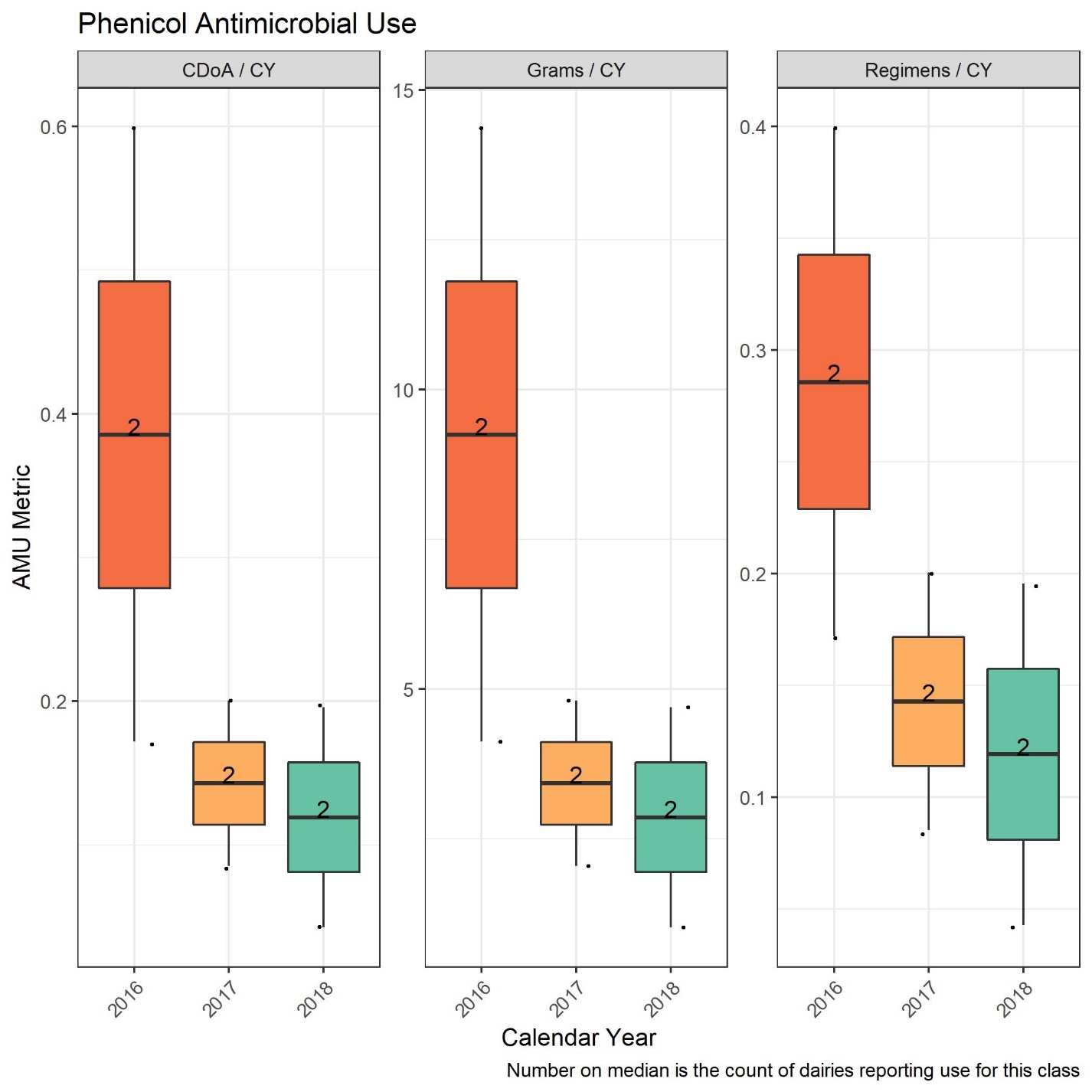

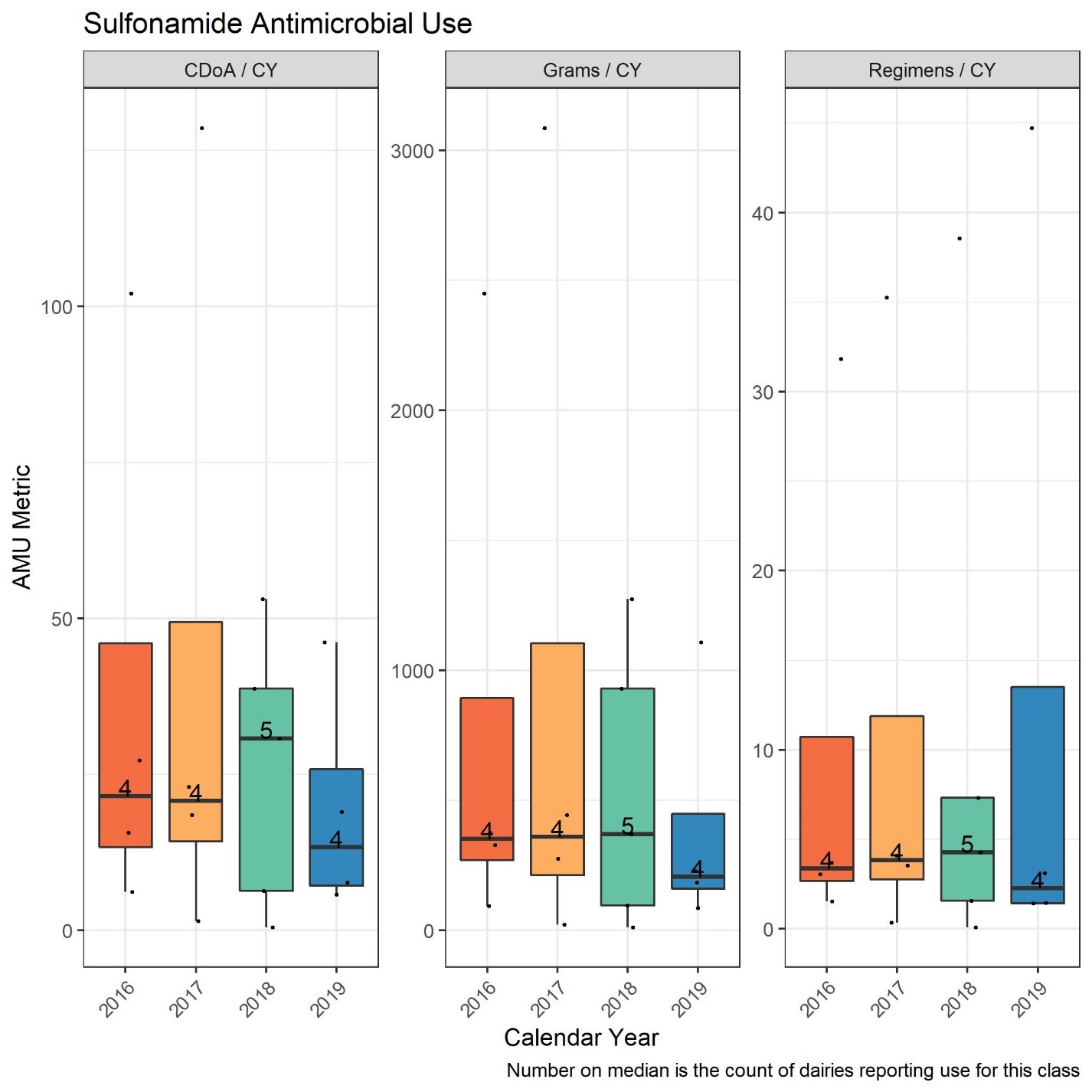

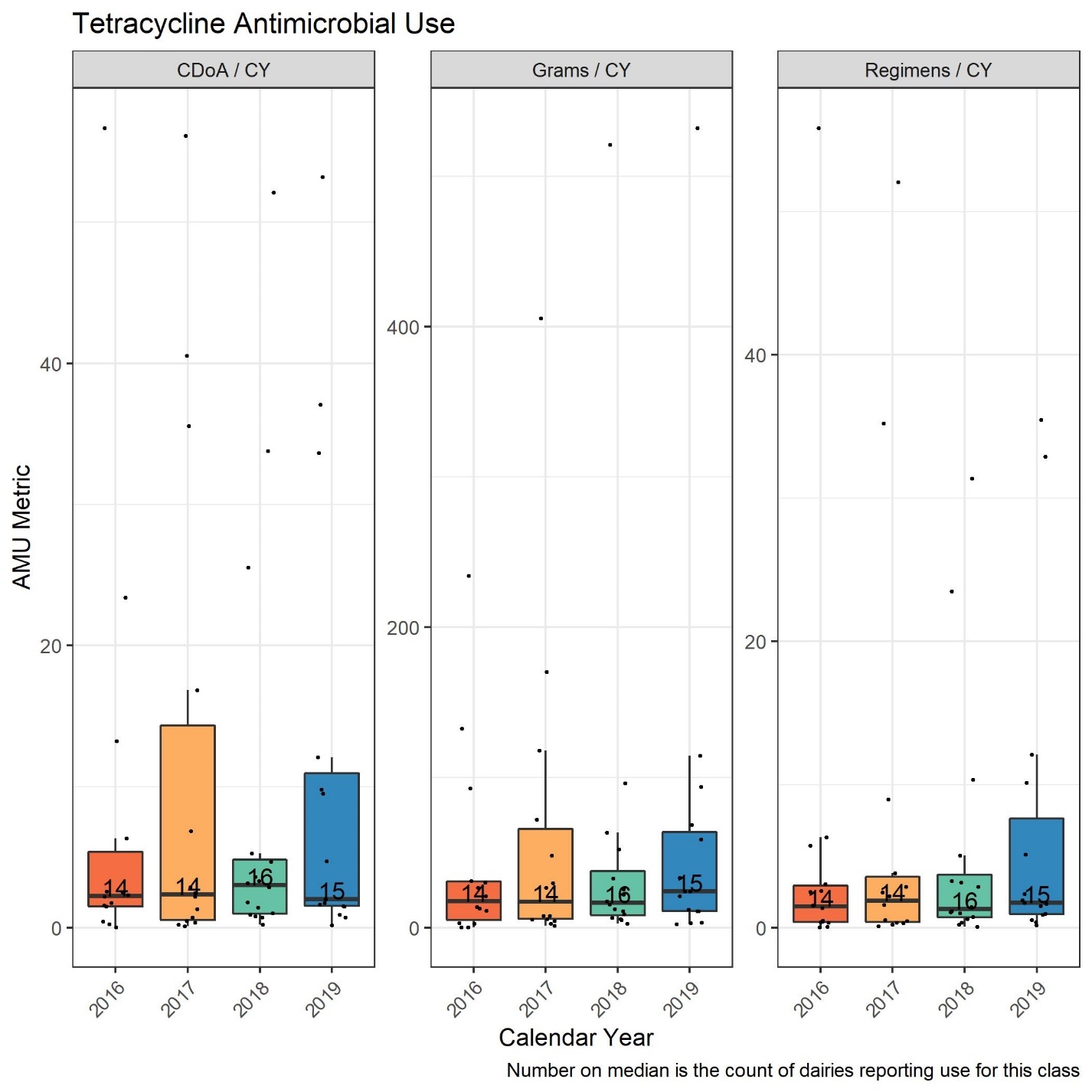

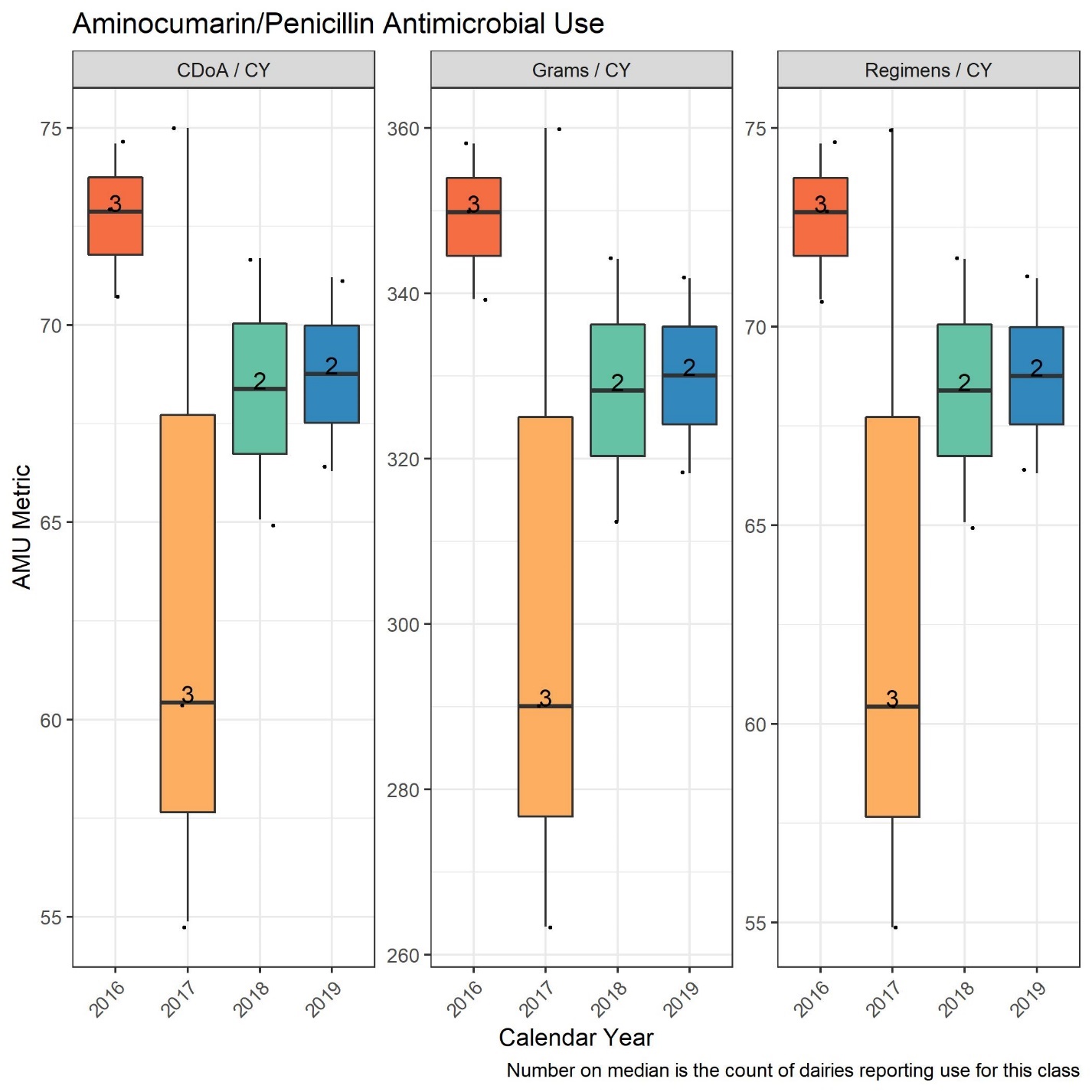


Schrag, N.F.D., Apley, M.D., Godden, S.M., Lubbers, B.V., and Singer, R.S. (2020). Antimicrobial use quantification in adult dairy cows - Part 1 - Standardized regimens as a method for describing antimicrobial use. *Zoonoses Public Health* 67 Suppl 1**,** 51-68. doi: 10.1111/zph.12766.
